# Supplementary material for: Genome-wide association meta-analyses and fine-mapping elucidate pathways influencing albuminuria
Source: Nat Commun. 2019 Sep 11;10:4130. doi: 10.1038/s41467-019-11576-0 (PMC6739370; doi:10.1038/s41467-019-11576-0)
Supplement: Supplementary file 2 — Supplementary Information [file 41467_2019_11576_MOESM2_ESM.pdf]

Genome-wide association meta-analyses and fine-mapping elucidate pathways influencing albuminuria

Teumer *et al.*

SUPPLEMENTARY INFORMATION

Table of Contents

Supplementary Note 1: Extended acknowledgements

Supplementary Figure 1: Workflow of the project

Supplementary Figure 2: Regional association plots for loci identified in trans-ethnic GWAS meta-analysis of UACR

Supplementary Figure 3: Expected number of discoveries at increasing GWAS discovery sample sizes.

Supplementary Figure 4: Regional association plots for loci identified in trans-ethnic GWAS meta-analysis of UACR among individuals with diabetes

Supplementary Figure 5: Manhattan plots for all secondary GWAS meta-analyses

Supplementary Figure 6: QQ plots plots for all GWAS meta-analyses

Supplementary Figure 7: Enrichment analysis results for physiological system where UACR-associated genes are highly expressed

Supplementary Figure 8: Co-localization of UACR-association signals with gene expression in *cis* across 47 human tissues

Supplementary Figure 9: The *Drosophila OAF* and *PRKCI* are both required for nephrocyte function and PRKCI-RNAi affects slit diaphragm formation

Supplementary Table 1: Association results of the UACR genetic risk score quartiles with microalbuminuria in the UKBB

Supplementary Table 2: Association results of the genetic risk score (GRS) analyses of UACR and it's risk factors

Supplementary Table 3: Evidence for additional candidate causal genes at UACR-associated variants

Supplementary Table 4: Results of the trans-eQTL analysis

Supplementary Table 5: Co-localization results of UACR association signals with those from associations with plasma protein levels

References

Supplementary Note 1: Extended acknowledgements

The Genotype-Tissue Expression (GTEx) Project[1] was supported by the Common Fund of the Office of the Director of the National Institutes of Health, and by NCI, NHGRI, NHLBI, NIDA, NIMH, and NINDS.

Variant annotation was supported by software resources provided via the Caché Campus program of the InterSystems GmbH to Alexander Teumer.

Data on coronary artery disease have been contributed by CARDIoGRAMplusC4D investigators and have been downloaded from www.CARDIOGRAMPLUSC4D.ORG.

The MEGASTROKE project received funding from sources specified at http://www.megastroke.org/acknowledgments.html. The complete list of all MEGASTROKE contributors is provided at the end of the Acknowledgements.

Study-specific acknowledgements and funding sources

AA-DHS NIH R01 DK071891 (Barry I. Freedman, PI); NIH R01 NS075107 (Barry I. Freedman, PI).

ADVANCE ADVANCE genomic sub-studies were supported by grants from the Ministry of Science and Innovation from the Quebec Government, from Genome Quebec, from the Consortium Québécois du Médicament, from the Canadian Institutes of Health Research and from Medpharmgene, OPTITHERA Inc and Les Laboratoires Servier.

AGES This study has been funded by NIA contract N01-AG-12100 and HHSN271201200022C with contributions from NEI, NIDCD and NHLBI, the NIA Intramural Research Program, Hjartavernd (the Icelandic Heart Association), and the Althingi (the Icelandic Parliament). The study is approved by the Icelandic National Bioethics Committee, VSN: 00-063. The researchers thank the participants for their willingness to participate in the study.

Amish We thank the Amish research volunteers for their long-standing partnership in research, and the research staff at the Amish Research Clinic for their work and dedication. The Amish contribution was supported by NIH grants R01 AG18728, R01 HL088119, U01 GM074518, U01 HL072515, U01 HL084756, and NIH K12RR023250, and P30 DK072488. Additional support was provided by the University of Maryland General Clinical Research Center, grant M01 RR 16500, the Baltimore Veterans Administration Medical Center Geriatrics Research, and the Paul Beeson Physician Faculty Scholars in Aging Program.

ARIC The Atherosclerosis Risk in Communities study has been funded in whole or in part with Federal funds from the National Heart, Lung, and Blood Institute, National Institutes of Health, Department of Health and Human Services (contract numbers HHSN268201700001I, HHSN268201700002I, HHSN268201700003I, HHSN268201700004I and HHSN268201700005I), R01HL087641, R01HL086694; National Human Genome Research Institute contract U01HG004402; and National Institutes of Health contract HHSN268200625226C. The authors thank the staff and participants of the ARIC study for their important contributions. Infrastructure was partly supported by Grant Number UL1RR025005, a component of the National Institutes of Health and NIH Roadmap for Medical Research. The work of Anna Köttgen was supported by a Heisenberg Professorship (KO 3598/3-1) as well as CRC 1140 Projektnummer 246781735 and CRC 992 of the German Research Foundation.

CHRIS Full acknowledgements for the CHRIS study are reported at http://translational-medicine.biomedcentral.com/articles/10.1186/s12967-015-0704-9#Declarations. The CHRIS study was funded by the Department of Innovation, Research, and University of the Autonomous Province of Bolzano-South Tyrol.

CHS Cardiovascular Health Study: This CHS research was supported by NHLBI contracts HHSN268201200036C, HHSN268200800007C, HHSN268201800001C, HHSN268200960009C, N01HC55222, N01HC85079, N01HC85080, N01HC85081, N01HC85082, N01HC85083, N01HC85086; and NHLBI grants U01HL080295, R01HL087652, R01HL105756, R01HL103612, R01HL120393, R01HL085251, and U01HL130114 with additional contribution from the National Institute of Neurological Disorders and Stroke (NINDS). Additional support was provided through R01AG023629 from the National Institute on Aging (NIA). A full list of principal CHS investigators and institutions can be found at CHSNHLBI.org. The provision of genotyping data was supported in part by the National Center for Advancing Translational Sciences, CTSI grant UL1TR001881, and the National Institute of Diabetes and Digestive and Kidney Disease Diabetes Research Center (DRC) grant DK063491 to the Southern California Diabetes Endocrinology Research Center. The content is solely the responsibility of the authors and does not necessarily represent the official views of the National Institutes of Health.

DECODE The study was funded by deCODE Genetics/Amgen inc. We thank the study subjects for their valuable participation and our colleagues, who contributed to data collection, sample handling, and genotyping.

DIACORE Cohort recruiting and management was funded by the KfH Stiftung Präventivmedizin e.V. (Carsten A. Böger). Genome-wide genotyping was funded by the Else Kröner-Fresenius-Stiftung (2012_A147), the KfH Stiftung Präventivmedizin and the University Hospital Regensburg. Data analysis was funded by the Else Kröner-Fresenius Stiftung (2012_A147) and by the DFG with grant number BO 3815/4-1. The work of Iris M. Heid and Carsten A. Böger was supported by DFG CRC 1350/C6 and DFG BO 3815/4-1.

ESTHER The ESTHER study was funded by the Saarland state Ministry for Social Affairs, Health, Women and Family Affairs (Saarbrücken, Germany), the Baden-Württemberg state Ministry of Science, Research and Arts (Stuttgart, Germany), the Federal Ministry of Education and Research (Berlin, Germany) and the Federal Ministry of Family Affairs, Senior Citizens, Women and Youth (Berlin, Germany).

FHS The Framingham Heart Study is supported by HHSN268201500001.

FINCAVAS The Finnish Cardiovascular Study (FINCAVAS) has been financially supported by the Competitive Research Funding of the Tampere University Hospital (Grant 9M048 and 9N035), the Finnish Cultural Foundation, the Finnish Foundation for Cardiovascular Research, the Emil Aaltonen Foundation, Finland, the Tampere Tuberculosis Foundation, and EU Horizon 2020 (grant 755320 for TAXINOMISIS). The authors thank the staff of the Department of Clinical Physiology for collecting the exercise test data.

GCKD The GCKD study was funded by the German Ministry of Research and Education (Bundesminsterium für Bildung und Forschung, BMBF) and by the Foundation KfH Stiftung Präventivmedizin. Unregistered grants to support the study were provided by Bayer, Fresenius Medical Care and Amgen. Genotyping was supported by Bayer Pharma AG. Uromodulin measurements in GCKD were supported by the Swiss National Centre of Competence in Research Kidney Control of Homeostasis program and the Swiss National Science Foundation grant 31003A_169850. The work of Matthias Wuttke was supported by DFG CRC 1140 and the Else Kroener Fresenius Forschungskolleg NAKSYS. The work of Yong Li was supported by DFG KO 3598/4-1.

The work of Anselm Hoppmann was partly supported by NIDDK R01 DK087635-09.

Generation R The Generation R Study is conducted by the Erasmus Medical Center in close collaboration with the School of Law and Faculty of Social Sciences of the Erasmus University Rotterdam, the Municipal Health Service Rotterdam area, Rotterdam, the Rotterdam Homecare Foundation, Rotterdam and the Stichting Trombosedienst & Artsenlaboratorium Rijnmond (STAR-MDC), Rotterdam. We acknowledge the contribution of children and parents, general practitioners, hospitals, midwives and pharmacies in Rotterdam. The generation and management of GWAS genotype data for the Generation R Study was done at the Genetic Laboratory of the Department of Internal Medicine, Erasmus MC, the Netherlands. We would like to thank Karol Estrada, Dr. Tobias A. Knoch, Anis Abuseiris, Luc V. de Zeeuw, and Rob de Graaf, for their help in creating GRIMP, BigGRID, MediGRID, and Services@MediGRID/D-Grid, [funded by the German Bundesministerium fuer Forschung und Technology; grants 01 AK 803 A-H, 01 IG 07015 G] for access to their grid computing resources. We thank Pascal Arp, Mila Jhamai, Marijn Verkerk, Manoushka Ganesh, Lizbeth Herrera and Marjolein Peters for their help in creating, managing and QC of the GWAS database. The general design of Generation R Study is made possible by financial support from the Erasmus MC, University Medical Center, Rotterdam, the Netherlands Organization for Health Research and Development (ZonMw) and the Ministry of Health, Welfare and Sport. Fernando Rivadeneira received additional funding from the Netherlands Organization for Health Research and Development (VIDI 016. 136. 367). This project received additional funding from the European Union's Horizon 2020 research and innovation programme under grant agreements No 633595 (DynaHEALTH) and No 733206 (LifeCycle).

JHS JHS acknowledgement. The Jackson Heart Study (JHS) is supported and conducted in collaboration with Jackson State University (HHSN268201800013I), Tougaloo College (HHSN268201800014I), the Mississippi State Department of Health (HHSN268201800015I) and the University of Mississippi Medical Center (HHSN268201800010I, HHSN268201800011I and HHSN268201800012I) contracts from the National Heart, Lung, and Blood Institute (NHLBI) and the National Institute for Minority Health and Health Disparities (NIMHD). The authors also wish to thank the staffs and participants of the JHS. James G. Wilson is supported by U54GM115428 from the National Institute of General Medical Sciences. Laura M. Raffield is supported by T32 HL129982.

JHS disclaimer. The views expressed in this manuscript are those of the authors and do not necessarily represent the views of the National Heart, Lung, and Blood Institute; the National Institutes of Health; or the U.S. Department of Health and Human Services.

KORA The KORA research platform (KORA, Cooperative Health Research in the Region of Augsburg) was initiated and financed by the Helmholtz Zentrum München - German Research Center for Environmental Health, which is funded by the German Federal Ministry of Education (BMBF) and Research and by the State of Bavaria. Furthermore, KORA research was supported within the Munich Center of Health Sciences (MC Health), Ludwig-Maximilians-Universität, as part of LMUinnovativ. Statistical KORA analyses were supported by DFG BO-3815/4-1 (to Carsten A. Böger) and BMBF 01ER1206 and 01ER1507 (to Iris M. Heid) and by University of Regensburg.

LIFE-Adult, LIFE-Child LIFE-Adult and LIFE-Child are funded by the Leipzig Research Center for Civilization Diseases (LIFE). LIFE is an organizational unit affiliated to the Medical Faculty of the University of Leipzig. LIFE is funded by means of the European Union, by the European Regional Development Fund (ERDF) and by funds of the Free State of

	Saxony within the framework of the excellence initiative.
**Lifelines**	The Lifelines Cohort Study, and generation and management of GWAS genotype data for the Lifelines Cohort Study is supported by the Netherlands Organization of Scientific Research NWO (grant 175.010.2007.006), the Economic Structure Enhancing Fund (FES) of the Dutch government, the Ministry of Economic Affairs, the Ministry of Education, Culture and Science, the Ministry for Health, Welfare and Sports, the Northern Netherlands Collaboration of Provinces (SNN), the Province of Groningen, University Medical Center Groningen, the University of Groningen, Dutch Kidney Foundation and Dutch Diabetes Research Foundation. The authors wish to acknowledge the services of the Lifelines Cohort Study, the contributing research centers delivering data to Lifelines, and all the study participants.
**Living-biobank**	The genotyping was funded by the Agency for Science, Technology and Research, Singapore (https://www.a-star.edu.sg/), and by Merck Sharp & Dohme Corp., Whitehouse Station, NJ USA (http://www.merck.com). The participants were from the MEC study (funded by the Biomedical Research Council (BMRC 03/1/27/18/216), National Medical Research Council (0838/2004), National Research Foundation (through BMRC 05/1/21/19/425 and 11/1/21/19/678), Ministry of Health, Singapore, National University of Singapore and National University Health System, Singapore), and The SH2012 study (funded by the Ministry of Health, Singapore, National University of Singapore and National University Health System, Singapore).
**MESA**	MESA and the MESA SHARe project are conducted and supported by the National Heart, Lung, and Blood Institute (NHLBI) in collaboration with MESA investigators. Support for MESA is provided by contracts HHSN268201500003I, N01-HC-95159, N01-HC-95160, N01-HC-95161, N01-HC-95162, N01-HC-95163, N01-HC-95164, N01-HC-95165, N01-HC-95166, N01-HC-95167, N01-HC-95168, N01-HC-95169, UL1-TR-000040, UL1-TR-001079, UL1-TR-001420, UL1-TR-001881, and DK063491. MESA Family is conducted and supported by the National Heart, Lung, and Blood Institute (NHLBI) in collaboration with MESA investigators. Support is provided by grants and contracts R01HL071051, R01HL071205, R01HL071250, R01HL071251, R01HL071258, R01HL071259, by the National Center for Research Resources, Grant UL1RR033176, and the National Center for Advancing Translational Sciences, Grant UL1TR001881. This publication was developed under a STAR research assistance agreement, No. RD831697 (MESA Air), awarded by the U.S Environmental protection Agency. It has not been formally reviewed by the EPA. The views expressed in this document are solely those of the authors and the EPA does not endorse any products or commercial services mentioned in this publication.
**MICROS**	We thank all study participants, all primary care practitioners, and the personnel of the Hospital of Silandro (Department of Laboratory Medicine) for their participation and collaboration in the research project. The study was supported by the Ministry of Health and Department of Educational Assistance, University and Research of the Autonomous Province of Bolzano, the South Tyrolean Sparkasse Foundation, and the European Union framework program 6 EUROSPAN project (contract no. LSHG-CT-2006-018947).
**MyCode (Geisinger)**	We would like to acknowledge the participants, staff, and our colleagues associated with the Geisinger MyCode Community Health Initiative. We also thank the staff of the PACDC of Geisinger for assistance with the phenotypic data, and the staff of the Biomedical & Translational Informatics and Kidney Health Research Institute.
**NEO**	The authors of the NEO study thank all individuals who participated in the Netherlands Epidemiology in Obesity study, all participating general practitioners

for inviting eligible participants and all research nurses for collection of the data. We thank the NEO study group, Pat van Beelen, Petra Noordijk and Ingeborg de Jonge for the coordination, lab and data management of the NEO study. The genotyping in the NEO study was supported by the Centre National de Génotypage (Paris, France), headed by Jean-Francois Deleuze. The NEO study is supported by the participating Departments, the Division and the Board of Directors of the Leiden University Medical Center, and by the Leiden University, Research Profile Area Vascular and Regenerative Medicine. Dennis Mook-Kanamori is supported by Dutch Science Organization (ZonMW-VENI Grant 916.14.023).

PIVUS The PIVUS study was supported by Wellcome Trust grants WT098017, WT064890, WT090532, Uppsala University, Uppsala University Hospital, the Swedish Research Council and the Swedish Heart-Lung Foundation. Cecilia M. Lindgren is supported by the Li Ka Shing Foundation, WT-SSI/John Fell funds and by the NIHR Biomedical Research Centre, Oxford, by Widenlife and NIH (5P50HD028138-27). Johan Ärnlöv was supported by The Swedish Research Council, Swedish Heart-Lung Foundation, Dalarna University and Uppsala University

POPGEN The PopGen 2.0 network was supported by a grant from the German Ministry for Education and Research (01EY1103). Sandra Freitag-Wolf was supported by German Research Foundation, Clusters of Excellence 306, Inflammation at Interfaces.

PREVEND The Prevention of Renal and Vascular Endstage Disease Study (PREVEND) genetics is supported by the Dutch Kidney Foundation (Grant E033), the EU project grant GENECURE (FP-6 LSHM CT 2006 037697), the National Institutes of Health (grant LM010098), the Netherlands organization for health research and development (NWO VENI grant 916.761.70), and the Dutch Inter University Cardiology Institute Netherlands (ICIN). Niek Verweij was supported by NWO VENI grant 016.186.125.

RS The Rotterdam Study (RS) has been approved by the Medical Ethics Committee of the Erasmus MC (registration number MEC 02.1015) and by the Dutch Ministry of Health, Welfare and Sport (Population Screening Act WBO, license number 1071272-159521-PG). The RS has been entered into the Netherlands National Trial Register (NTR; www.trialregister.nl) and into the WHO International Clinical Trials Registry Platform (ICTRP; www.who.int/ictrp/network/primary/en/) under shared catalogue number NTR6831. All participants provided written informed consent to participate in the study and to have their information obtained from treating physicians. The generation and management of GWAS genotype data for the RS (RS I, RS II, RS III) was executed by the Human Genotyping Facility of the Genetic Laboratory of the Department of Internal Medicine, Erasmus MC, Rotterdam, The Netherlands. The GWAS datasets are supported by the Netherlands Organisation of Scientific Research NWO Investments (nr. 175.010.2005.011, 911-03-012), the Genetic Laboratory of the Department of Internal Medicine, Erasmus MC, the Research Institute for Diseases in the Elderly (014-93-015; RIDE2), the Netherlands Genomics Initiative (NGI)/Netherlands Organisation for Scientific Research (NWO) Netherlands Consortium for Healthy Aging (NCHA), project nr. 050-060-810. We thank Pascal Arp, Mila Jhamai, Marijn Verkerk, Lizbeth Herrera and Marjolein Peters, MSc, and Carolina Medina-Gomez, MSc, for their help in creating the GWAS database, and Karol Estrada, PhD, Yurii Aulchenko, PhD, and Carolina Medina-Gomez, MSc, for the creation and analysis of imputed data.
The RS is funded by Erasmus Medical Center and Erasmus University, Rotterdam, Netherlands Organization for the Health Research and Development (ZonMw), the Research Institute for Diseases in the Elderly (RIDE), the Ministry of Education, Culture and Science, the Ministry for Health, Welfare and Sports, the European Commission (DG XII), and the Municipality of Rotterdam. The authors are grateful to the study participants, the staff from the RS and the participating general

practitioners and pharmacists.

SCES The Singapore Chinese Eye Study (SCES) was supported by grants from the National Medical Research Council (STaR/0003/2008), the Singapore Bio Imaging Consortium (C-011/2006) and the Biomedical Research Council (08/1/35/19/550). The Genome Institute of Singapore provided services for genotyping.

SHIP/SHIP-Trend SHIP is part of the Community Medicine Research net of the University of Greifswald, Germany, which is funded by the Federal Ministry of Education and Research (grants no. 01ZZ9603, 01ZZ0103, and 01ZZ0403), the Ministry of Cultural Affairs as well as the Social Ministry of the Federal State of Mecklenburg-West Pomerania, and the network 'Greifswald Approach to Individualized Medicine (GANI_MED)' funded by the Federal Ministry of Education and Research (grant 03IS2061A). Genome-wide data have been supported by the Federal Ministry of Education and Research (grant no. 03ZIK012) and a joint grant from Siemens Healthineers, Erlangen, Germany and the Federal State of Mecklenburg- West Pomerania. The University of Greifswald is a member of the Caché Campus program of the InterSystems GmbH.

SiMES The Singapore Malay Eye Study (SiMES) was funded by the National Medical Research Council (NMRC 0796/2003 and NMRC/STaR/0003/2008) and Biomedical Research Council (BMRC, 09/1/35/19/616). The Genome Institute of Singapore provided services for genotyping.

SINDI The Singapore Indian Eye Study (SINDI) was funded by grants from the Biomedical Research Council of Singapore (BMRC 09/1/35/19/616 and 08/1/35/19/550), and the National Medical Research Council of Singapore (NMRC/STaR/0003/2008). The Genome Institute of Singapore provided services for genotyping.

SKIPOGH The SKIPOGH study was supported by a Swiss national science foundation grant (FN33CM30-124087 and FN33CM30-140331), by the Swiss National Centres of Competence in Research Kidney and TransCure.

Sorbs This work was supported by grants from the German Research Foundation (SFB-1052 ''Obesity mechanisms'' A01, B03, SPP 1629 TO 718/2- 1), from the German Diabetes Association, from the DHFD (Diabetes Hilfs- und Forschungsfonds Deutschland) and from IFB Adiposity Diseases (AD2-060E, AD2-06E95, AD2-06E99). IFB Adiposity Diseases is supported by the Federal Ministry of Education and Research (BMBF), Germany, FKZ: 01EO1501.

SP2 The Singapore Prospective Study Program (SP2) were supported by the individual research grant and clinician scientist award schemes from the National Medical Research Council (NMRC) and the Biomedical Research Council (BMRC) of Singapore. The Genome Institute of Singapore provided services for genotyping.

ULSAM The ULSAM study was supported by Wellcome Trust grants WT098017, WT064890, WT090532, Uppsala University, Uppsala University Hospital, the Swedish Research Council and the Swedish Heart-Lung Foundation. Johan Ärnlöv was supported by The Swedish Research Council, Swedish Heart-Lung Foundation, Dalarna University and Uppsala University.

Vanderbilt Otis Wilson & Adriana Hung were also supported by the Vanderbilt Center for Kidney Disease and Division of Nephrology & Hypertension. Ayush Giri is supported by the Building Interdisciplinary Research Careers in Women's Health career development program's 2K12HD043483-17 (PI: Katherine E Hartmann).

Veterans Affairs This work was supported by the Department Veterans Affairs MVP grant BX003360 (Hung). Ayush Giri, and Adriana M. Hung were supported by BX003360 and CX000982. This research is based on data from the Million Veteran Program, Office of Research and Development, Veterans Health Administration. Phecodes creation

	was supported using resources and facilities of the VA Informatics and Computing Infrastructure (VINCI), VA HSR RES 13-457.
**Functional studies**	T.H. was supported by a grant from the Deutsche Forschungsgemeinschaft (DFG) (HE 7456/3-1).

LifeLines Cohort Study: group author genetics acknowledgement

Behrooz Z Alizadeh (1), H Marike Boezen (1), Lude Franke (2), Pim van der Harst (3), Gerjan Navis (4), Marianne G. Rots (5), Morris Swertz (2), Bruce HR Wolffenbuttel (6), Cisca Wijmenga (2)

(1) Department of Epidemiology, University of Groningen, University Medical Center Groningen, The Netherlands
(2) Department of Genetics, University of Groningen, University Medical Center Groningen, The Netherlands
(3) Department of Cardiology, University of Groningen, University Medical Center Groningen, The Netherlands
(4) Department of Internal Medicine, Division of Nephrology, University of Groningen, University Medical Center Groningen, The Netherlands
(5) Department of Pathology and Medical Biology, University of Groningen, University Medical Center Groningen, The Netherlands
(6) Department of Endocrinology, University of Groningen, University Medical Center Groningen, The Netherlands

Million Veteran Program: consortium acknowledgement

MVP Executive Committee
- Co-Chair: J. Michael Gaziano, M.D., M.P.H.
- Co-Chair: Rachel Ramoni, D.M.D., Sc.D.
- Jim Breeling, M.D. (ex-officio)
- Kyong-Mi Chang, M.D.
- Grant Huang, Ph.D.
- Sumitra Muralidhar, Ph.D.
- Christopher J. O'Donnell, M.D., M.P.H.
- Philip S. Tsao, Ph.D.

MVP Program Office
- Sumitra Muralidhar, Ph.D.
- Jennifer Moser, Ph.D.

MVP Recruitment/Enrollment
- Recruitment/Enrollment Director/Deputy Director, Boston – Stacey B. Whitbourne, Ph.D.; Jessica V. Brewer, M.P.H.
- MVP Coordinating Centers
    - Clinical Epidemiology Research Center (CERC), West Haven – John Concato, M.D., M.P.H.
    - Cooperative Studies Program Clinical Research Pharmacy Coordinating Center, Albuquerque - Stuart Warren, J.D., Pharm D.; Dean P. Argyres, M.S.
    - Genomics Coordinating Center, Palo Alto – Philip S. Tsao, Ph.D.
    - Massachusetts Veterans Epidemiology Research Information Center (MAVERIC), Boston - J. Michael Gaziano, M.D., M.P.H.
    - MVP Information Center, Canandaigua – Brady Stephens, M.S.
- Core Biorepository, Boston – Mary T. Brophy M.D., M.P.H.; Donald E. Humphries, Ph.D.
- MVP Informatics, Boston – Nhan Do, M.D.; Shahpoor Shayan

- Data Operations/Analytics, Boston – Xuan-Mai T. Nguyen, Ph.D.

MVP Science

- Genomics - Christopher J. O'Donnell, M.D., M.P.H.; Saiju Pyarajan Ph.D.; Philip S. Tsao, Ph.D.
- Phenomics - Kelly Cho, M.P.H, Ph.D.
- Data and Computational Sciences – Saiju Pyarajan, Ph.D.
- Statistical Genetics – Elizabeth Hauser, Ph.D.; Yan Sun, Ph.D.; Hongyu Zhao, Ph.D.

MVP Local Site Investigators

- Atlanta VA Medical Center (Peter Wilson)
- Bay Pines VA Healthcare System (Rachel McArdle)
- Birmingham VA Medical Center (Louis Dellitalia)
- Cincinnati VA Medical Center (John Harley)
- Clement J. Zablocki VA Medical Center (Jeffrey Whittle)
- Durham VA Medical Center (Jean Beckham)
- Edith Nourse Rogers Memorial Veterans Hospital (John Wells)
- Edward Hines, Jr. VA Medical Center (Salvador Gutierrez)
- Fayetteville VA Medical Center (Gretchen Gibson)
- VA Health Care Upstate New York (Laurence Kaminsky)
- New Mexico VA Health Care System (Gerardo Villareal)
- VA Boston Healthcare System (Scott Kinlay)
- VA Western New York Healthcare System (Junzhe Xu)
- Ralph H. Johnson VA Medical Center (Mark Hamner)
- Wm. Jennings Bryan Dorn VA Medical Center (Kathlyn Sue Haddock)
- VA North Texas Health Care System (Sujata Bhushan)
- Hampton VA Medical Center (Pran Iruvanti)
- Hunter Holmes McGuire VA Medical Center (Michael Godschalk)
- Iowa City VA Health Care System (Zuhair Ballas)
- Jack C. Montgomery VA Medical Center (Malcolm Buford)
- James A. Haley Veterans' Hospital (Stephen Mastorides)
- Louisville VA Medical Center (Jon Klein)
- Manchester VA Medical Center (Nora Ratcliffe)
- Miami VA Health Care System (Hermes Florez)
- Michael E. DeBakey VA Medical Center (Alan Swann)
- Minneapolis VA Health Care System (Maureen Murdoch)
- N. FL/S. GA Veterans Health System (Peruvemba Sriram)
- Northport VA Medical Center (Shing Shing Yeh)
- Overton Brooks VA Medical Center (Ronald Washburn)
- Philadelphia VA Medical Center (Darshana Jhala)
- Phoenix VA Health Care System (Samuel Aguayo)
- Portland VA Medical Center (David Cohen)
- Providence VA Medical Center (Satish Sharma)
- Richard Roudebush VA Medical Center (John Callaghan)
- Salem VA Medical Center (Kris Ann Oursler)
- San Francisco VA Health Care System (Mary Whooley)
- South Texas Veterans Health Care System (Sunil Ahuja)
- Southeast Louisiana Veterans Health Care System (Amparo Gutierrez)
- Current as of 19-JUL-2018
- Southern Arizona VA Health Care System (Ronald Schifman)
- Sioux Falls VA Health Care System (Jennifer Greco)
- St. Louis VA Health Care System (Michael Rauchman)
- Syracuse VA Medical Center (Richard Servatius)
- VA Eastern Kansas Health Care System (Mary Oehlert)

- VA Greater Los Angeles Health Care System (Agnes Wallbom)
- VA Loma Linda Healthcare System (Ronald Fernando)
- VA Long Beach Healthcare System (Timothy Morgan)
- VA Maine Healthcare System (Todd Stapley)
- VA New York Harbor Healthcare System (Scott Sherman)
- VA Pacific Islands Health Care System (Gwenevere Anderson)
- VA Palo Alto Health Care System (Philip Tsao)
- VA Pittsburgh Health Care System (Elif Sonel)
- VA Puget Sound Health Care System (Edward Boyko)
- VA Salt Lake City Health Care System (Laurence Meyer)
- VA San Diego Healthcare System (Samir Gupta)
- VA Southern Nevada Healthcare System (Joseph Fayad)
- VA Tennessee Valley Healthcare System (Adriana Hung)
- Washington DC VA Medical Center (Jack Lichy)
- W.G. (Bill) Hefner VA Medical Center (Robin Hurley)
- White River Junction VA Medical Center (Brooks Robey)
- William S. Middleton Memorial Veterans Hospital (Robert Striker)

MEGASTROKE CONSORTIUM

Rainer Malik [1], Ganesh Chauhan [2], Matthew Traylor [3], Muralidharan Sargurupremraj [4,5], Yukinori Okada [6,7,8], Aniket Mishra [4,5], Loes Rutten-Jacobs [3], Anne-Katrin Giese [9], Sander W van der Laan [10], Solveig Gretarsdottir [11], Christopher D Anderson [12,13,14,14], Michael Chong [15], Hieab HH Adams [16,17], Tetsuro Ago [18], Peter Almgren [19], Philippe Amouyel [20,21], Hakan Ay [22,13], Traci M Bartz [23], Oscar R Benavente [24], Steve Bevan [25], Giorgio B Boncoraglio [26], Robert D Brown, Jr. [27], Adam S Butterworth [28,29], Caty Carrera [30,31], Cara L Carty [32,33], Daniel I Chasman [34,35], Wei-Min Chen [36], John W Cole [37], Adolfo Correa [38], Ioana Cotlarciuc [39], Carlos Cruchaga [40,41], John Danesh [28,42,43,44], Paul IW de Bakker [45,46], Anita L DeStefano [47,48], Marcel den Hoed [49], Qing Duan [50], Stefan T Engelter [51,52], Guido J Falcone [53,54], Rebecca F Gottesman [55], Raji P Grewal [56], Vilmundur Gudnason [57,58], Stefan Gustafsson [59], Jeffrey Haessler [60], Tamara B Harris [61], Ahamad Hassan [62], Aki S Havulinna [63,64], Susan R Heckbert [65], Elizabeth G Holliday [66,67], George Howard [68], Fang-Chi Hsu [69], Hyacinth I Hyacinth [70], M Arfan Ikram [16], Erik Ingelsson [71,72], Marguerite R Irvin [73], Xueqiu Jian [74], Jordi Jiménez-Conde [75], Julie A Johnson [76,77], J Wouter Jukema [78], Masahiro Kanai [6,7,79], Keith L Keene [80,81], Brett M Kissela [82], Dawn O Kleindorfer [82], Charles Kooperberg [60], Michiaki Kubo [83], Leslie A Lange [84], Carl D Langefeld [85], Claudia Langenberg [86], Lenore J Launer [87], Jin-Moo Lee [88], Robin Lemmens [89,90], Didier Leys [91], Cathryn M Lewis [92,93], Wei-Yu Lin [28,94], Arne G Lindgren [95,96], Erik Lorentzen [97], Patrik K Magnusson [98], Jane Maguire [99], Ani Manichaikul [36], Patrick F McArdle [100], James F Meschia [101], Braxton D Mitchell [100,102], Thomas H Mosley [103,104], Michael A Nalls [105,106], Toshiharu Ninomiya [107], Martin J O'Donnell [15,108], Bruce M Psaty [109,110,111,112], Sara L Pulit [113,45], Kristiina Rannikmäe [114,115], Alexander P Reiner [65,116], Kathryn M Rexrode [117], Kenneth Rice [118], Stephen S Rich [36], Paul M Ridker [34,35], Natalia S Rost [9,13], Peter M Rothwell [119], Jerome I Rotter [120,121], Tatjana Rundek [122], Ralph L Sacco [122], Saori Sakaue [7,123], Michele M Sale [124], Veikko Salomaa [63], Bishwa R Sapkota [125], Reinhold Schmidt [126], Carsten O Schmidt [127], Ulf Schminke [128], Pankaj Sharma [39], Agnieszka Slowik [129], Cathie LM Sudlow [114,115], Christian Tanislav [130], Turgut Tatlisumak [131,132], Kent D Taylor [120,121], Vincent NS Thijs [133,134], Gudmar Thorleifsson [11], Unnur Thorsteinsdottir [11], Steffen Tiedt [1], Stella Trompet [135], Christophe Tzourio [5,136,137], Cornelia M van Duijn [138,139], Matthew Walters [140], Nicholas J Wareham [86], Sylvia Wassertheil-Smoller [141], James G Wilson [142], Kerri L Wiggins [109], Qiong Yang [47], Salim Yusuf [15], Najaf Amin [16], Hugo S Aparicio [185,48], Donna K Arnett [186], John Attia [187], Alexa S Beiser [47,48], Claudine Berr [188], Julie E Buring [34,35], Mariana Bustamante [189], Valeria Caso [190], Yu-Ching Cheng [191], Seung Hoan Choi [192,48], Ayesha Chowhan [185,48], Natalia Cullell [31], Jean-François Dartigues [193,194], Hossein Delavaran [95,96], Pilar Delgado [195], Marcus Dörr [196,197], Gunnar Engström [19], Ian Ford [198], Wander S Gurpreet [199], Anders Hamsten [200,201], Laura Heitsch [202], Atsushi Hozawa [203], Laura Ibanez [204], Andreea Ilinca [95,96], Martin Ingelsson [205], Motoki Iwasaki [206], Rebecca D Jackson [207], Katarina Jood [208], Pekka Jousilahti [63], Sara Kaffashian [4,5], Lalit Kalra [209], Masahiro Kamouchi [210], Takanari Kitazono [211], Olafur Kjartansson [212], Manja

Kloss [213], Peter J Koudstaal [214], Jerzy Krupinski [215], Daniel L Labovitz [216], Cathy C Laurie [118], Christopher R Levi [217], Linxin Li [218], Lars Lind [219], Cecilia M Lindgren [220,221], Vasileios Lioutas [222,48], Yong Mei Liu [223], Oscar L Lopez [224], Hirata Makoto [225], Nicolas Martinez-Majander [172], Koichi Matsuda [225], Naoko Minegishi [203], Joan Montaner [226], Andrew P Morris [227,228], Elena Muiño [31], Martina Müller-Nurasyid [229,230,231], Bo Norrving [95,96], Soichi Ogishima [203], Eugenio A Parati [232], Leema Reddy Peddareddygari [56], Nancy L Pedersen [98,233], Joanna Pera [129], Markus Perola [63,234], Alessandro Pezzini [235], Silvana Pileggi [236], Raquel Rabionet [237], Iolanda Riba-Llena [30], Marta Ribasés [238], Jose R Romero [185,48], Jaume Roquer [239,240], Anthony G Rudd [241,242], Antti-Pekka Sarin [243,244], Ralhan Sarju [199], Chloe Sarnowski [47,48], Makoto Sasaki [245], Claudia L Satizabal [185,48], Mamoru Satoh [245], Naveed Sattar [246], Norie Sawada [206], Gerli Sibolt [172], Ásgeir Sigurdsson [247], Albert Smith [248], Kenji Sobue [245], Carolina Soriano-Tárraga [240], Tara Stanne [249], O Colin Stine [250], David J Stott [251], Konstantin Strauch [229,252], Takako Takai [203], Hideo Tanaka [253,254], Kozo Tanno [245], Alexander Teumer [255], Liisa Tomppo [172], Nuria P Torres-Aguila [31], Emmanuel Touze [256,257], Shoichiro Tsugane [206], Andre G Uitterlinden [258], Einar M Valdimarsson [259], Sven J van der Lee [16], Henry Völzke [255], Kenji Wakai [253], David Weir [260], Stephen R Williams [261], Charles DA Wolfe [241,242], Quenna Wong [118], Huichun Xu [191], Taiki Yamaji [206], Dharambir K Sanghera [125,169,170], Olle Melander [19], Christina Jern [171], Daniel Strbian [172,173], Israel Fernandez-Cadenas [31,30], W T Longstreth, Jr [174,65], Arndt Rolfs [175], Jun Hata [107], Daniel Woo [82], Jonathan Rosand [12,13,14], Guillaume Pare [15], Jemma C Hopewell [176], Danish Saleheen [177], Kari Stefansson [11,178], Bradford B Worrall [179], Steven J Kittner [37], Sudha Seshadri [180,48], Myriam Fornage [74,181], Hugh S Markus [3], Joanna MM Howson [28], Yoichiro Kamatani [6,182], Stephanie Debette [4,5], Martin Dichgans [1,183,184]

1 Institute for Stroke and Dementia Research (ISD), University Hospital, LMU Munich, Munich, Germany
2 Centre for Brain Research, Indian Institute of Science, Bangalore, India
3 Stroke Research Group, Division of Clinical Neurosciences, University of Cambridge, UK
4 INSERM U1219 Bordeaux Population Health Research Center, Bordeaux, France
5 University of Bordeaux, Bordeaux, France
6 Laboratory for Statistical Analysis, RIKEN Center for Integrative Medical Sciences, Yokohama, Japan
7 Department of Statistical Genetics, Osaka University Graduate School of Medicine, Osaka, Japan
8 Laboratory of Statistical Immunology, Immunology Frontier Research Center (WPI-IFReC), Osaka University, Suita, Japan.
9 Department of Neurology, Massachusetts General Hospital, Harvard Medical School, Boston, MA, USA
10 Laboratory of Experimental Cardiology, Division of Heart and Lungs, University Medical Center Utrecht, University of Utrecht, Utrecht,Netherlands
11 deCODE genetics/AMGEN inc, Reykjavik, Iceland
12 Center for Genomic Medicine, Massachusetts General Hospital (MGH), Boston, MA, USA
13 J. Philip Kistler Stroke Research Center, Department of Neurology, MGH, Boston, MA, USA
14 Program in Medical and Population Genetics, Broad Institute, Cambridge, MA, USA
15 Population Health Research Institute, McMaster University, Hamilton, Canada
16 Department of Epidemiology, Erasmus University Medical Center, Rotterdam, Netherlands
17 Department of Radiology and Nuclear Medicine, Erasmus University Medical Center, Rotterdam, Netherlands
18 Department of Medicine and Clinical Science, Graduate School of Medical Sciences, Kyushu University, Fukuoka, Japan
19 Department of Clinical Sciences, Lund University, Malmö, Sweden
20 Univ. Lille, Inserm, Institut Pasteur de Lille, LabEx DISTALZ-UMR1167, Risk factors and molecular determinants of aging-related diseases, F-59000 Lille, France
21 Centre Hosp. Univ Lille, Epidemiology and Public Health Department, F-59000 Lille, France
22 AA Martinos Center for Biomedical Imaging, Department of Radiology, Massachusetts General Hospital, Harvard Medical School, Boston, MA, USA
23 Cardiovascular Health Research Unit, Departments of Biostatistics and Medicine, University of Washington, Seattle, WA, USA
24 Division of Neurology, Faculty of Medicine, Brain Research Center, University of British Columbia, Vancouver, Canada
25 School of Life Science, University of Lincoln, Lincoln, UK
26 Department of Cerebrovascular Diseases, Fondazione IRCCS Istituto Neurologico "Carlo Besta", Milano, Italy
27 Department of Neurology, Mayo Clinic Rochester, Rochester, MN, USA
28 MRC/BHF Cardiovascular Epidemiology Unit, Department of Public Health and Primary Care, University of Cambridge, Cambridge, UK
29 The National Institute for Health Research Blood and Transplant Research Unit in Donor Health and Genomics, University of Cambridge, UK
30 Neurovascular Research Laboratory, Vall d'Hebron Institut of Research, Neurology and Medicine Departments-Universitat Autònoma de Barcelona, Vall d'Hebrón Hospital, Barcelona, Spain
31 Stroke Pharmacogenomics and Genetics, Fundacio Docència i Recerca MutuaTerrassa, Terrassa, Spain
32 Children's Research Institute, Children's National Medical Center, Washington, DC, USA
33 Center for Translational Science, George Washington University, Washington, DC, USA
34 Division of Preventive Medicine, Brigham and Women's Hospital, Boston, MA, USA
35 Harvard Medical School, Boston, MA, USA
36 Center for Public Health Genomics, Department of Public Health Sciences, University of Virginia, Charlottesville, VA, USA
37 Department of Neurology, University of Maryland School of Medicine and Baltimore VAMC, Baltimore, MD, USA
38 Departments of Medicine, Pediatrics and Population Health Science, University of Mississippi Medical Center, Jackson, MS, USA
39 Institute of Cardiovascular Research, Royal Holloway University of London, UK & Ashford and St Peters Hospital, Surrey UK
40 Department of Psychiatry,The Hope Center Program on Protein Aggregation and Neurodegeneration (HPAN),Washington University, School of Medicine, St. Louis, MO, USA
41 Department of Developmental Biology, Washington University School of Medicine, St. Louis, MO, USA
42 NIHR Blood and Transplant Research Unit in Donor Health and Genomics, Department of Public Health and Primary Care, University of Cambridge, Cambridge, UK
43 Wellcome Trust Sanger Institute, Wellcome Trust Genome Campus, Hinxton, Cambridge, UK
44 British Heart Foundation, Cambridge Centre of Excellence, Department of Medicine, University of Cambridge, Cambridge, UK
45 Department of Medical Genetics, University Medical Center Utrecht, Utrecht, Netherlands
46 Department of Epidemiology, Julius Center for Health Sciences and Primary Care, University Medical Center Utrecht, Utrecht, Netherlands
47 Boston University School of Public Health, Boston, MA, USA

48 Framingham Heart Study, Framingham, MA, USA
49 Department of Immunology, Genetics and Pathology and Science for Life Laboratory, Uppsala University, Uppsala, Sweden
50 Department of Genetics, University of North Carolina, Chapel Hill, NC, USA
51 Department of Neurology and Stroke Center, Basel University Hospital, Switzerland
52 Neurorehabilitation Unit, University and University Center for Medicine of Aging and Rehabilitation Basel, Felix Platter Hospital, Basel, Switzerland
53 Department of Neurology, Yale University School of Medicine, New Haven, CT, USA
54 Program in Medical and Population Genetics, The Broad Institute of Harvard and MIT, Cambridge, MA, USA
55 Department of Neurology, Johns Hopkins University School of Medicine, Baltimore, MD, USA
56 Neuroscience Institute, SF Medical Center, Trenton, NJ, USA
57 Icelandic Heart Association Research Institute, Kopavogur, Iceland
58 University of Iceland, Faculty of Medicine, Reykjavik, Iceland
59 Department of Medical Sciences, Molecular Epidemiology and Science for Life Laboratory, Uppsala University, Uppsala, Sweden
60 Division of Public Health Sciences, Fred Hutchinson Cancer Research Center, Seattle, WA, USA
61 Laboratory of Epidemiology and Population Science, National Institute on Aging, National Institutes of Health, Bethesda, MD, USA
62 Department of Neurology, Leeds General Infirmary, Leeds Teaching Hospitals NHS Trust, Leeds, UK
63 National Institute for Health and Welfare, Helsinki, Finland
64 FIMM - Institute for Molecular Medicine Finland, Helsinki, Finland
65 Department of Epidemiology, University of Washington, Seattle, WA, USA
66 Public Health Stream, Hunter Medical Research Institute, New Lambton, Australia
67 Faculty of Health and Medicine, University of Newcastle, Newcastle, Australia
68 School of Public Health, University of Alabama at Birmingham, Birmingham, AL, USA
69 Department of Biostatistical Sciences, Wake Forest School of Medicine, Winston-Salem, NC, USA
70 Aflac Cancer and Blood Disorder Center, Department of Pediatrics, Emory University School of Medicine, Atlanta, GA, USA
71 Department of Medicine, Division of Cardiovascular Medicine, Stanford University School of Medicine, CA, USA
72 Department of Medical Sciences, Molecular Epidemiology and Science for Life Laboratory, Uppsala University, Uppsala, Sweden
73 Epidemiology, School of Public Health, University of Alabama at Birmingham, USA
74 Brown Foundation Institute of Molecular Medicine, University of Texas Health Science Center at Houston, Houston, TX, USA
75 Neurovascular Research Group (NEUVAS), Neurology Department, Institut Hospital del Mar d'Investigació Mèdica, Universitat Autònoma de Barcelona, Barcelona, Spain
76 Department of Pharmacotherapy and Translational Research and Center for Pharmacogenomics, University of Florida, College of Pharmacy, Gainesville, FL, USA
77 Division of Cardiovascular Medicine, College of Medicine, University of Florida, Gainesville, FL, USA
78 Department of Cardiology, Leiden University Medical Center, Leiden, the Netherlands
79 Program in Bioinformatics and Integrative Genomics, Harvard Medical School, Boston, MA, USA
80 Department of Biology, East Carolina University, Greenville, NC, USA
81 Center for Health Disparities, East Carolina University, Greenville, NC, USA
82 University of Cincinnati College of Medicine, Cincinnati, OH, USA
83 RIKEN Center for Integrative Medical Sciences, Yokohama, Japan
84 Department of Medicine, University of Colorado Denver, Anschutz Medical Campus, Aurora, CO, USA
85 Center for Public Health Genomics and Department of Biostatistical Sciences, Wake Forest School of Medicine, Winston-Salem, NC, USA
86 MRC Epidemiology Unit, University of Cambridge School of Clinical Medicine, Institute of Metabolic Science, Cambridge Biomedical Campus, Cambridge, UK
87 Intramural Research Program, National Institute on Aging, National Institutes of Health, Bethesda, MD, USA
88 Department of Neurology, Radiology, and Biomedical Engineering, Washington University School of Medicine, St. Louis, MO, USA
89 KU Leuven – University of Leuven, Department of Neurosciences, Experimental Neurology, Leuven, Belgium
90 VIB Center for Brain & Disease Research, University Hospitals Leuven, Department of Neurology, Leuven, Belgium
91 Univ.-Lille, INSERM U 1171. CHU Lille. Lille, France
92 Department of Medical and Molecular Genetics, King's College London, London, UK
93 SGDP Centre, Institute of Psychiatry, Psychology & Neuroscience, King's College London, London, UK
94 Northern Institute for Cancer Research, Paul O'Gorman Building, Newcastle University, Newcastle, UK
95 Department of Clinical Sciences Lund, Neurology, Lund University, Lund, Sweden
96 Department of Neurology and Rehabilitation Medicine, Skåne University Hospital, Lund, Sweden
97 Bioinformatics Core Facility, University of Gothenburg, Gothenburg, Sweden
98 Department of Medical Epidemiology and Biostatistics, Karolinska Institutet, Stockholm, Sweden
99 University of Technology Sydney, Faculty of Health, Ultimo, Australia
100 Department of Medicine, University of Maryland School of Medicine, MD, USA
101 Department of Neurology, Mayo Clinic, Jacksonville, FL, USA
102 Geriatrics Research and Education Clinical Center, Baltimore Veterans Administration Medical Center, Baltimore, MD, USA
103 Division of Geriatrics, School of Medicine, University of Mississippi Medical Center, Jackson, MS, USA
104 Memory Impairment and Neurodegenerative Dementia Center, University of Mississippi Medical Center, Jackson, MS, USA
105 Laboratory of Neurogenetics, National Institute on Aging, National institutes of Health, Bethesda, MD, USA
106 Data Tecnica International, Glen Echo MD, USA
107 Department of Epidemiology and Public Health, Graduate School of Medical Sciences, Kyushu University, Fukuoka, Japan
108 Clinical Research Facility, Department of Medicine, NUI Galway, Galway, Ireland
109 Cardiovascular Health Research Unit, Department of Medicine, University of Washington, Seattle, WA, USA
110 Department of Epidemiology, University of Washington, Seattle, WA
111 Department of Health Services, University of Washington, Seattle, WA, USA
112 Kaiser Permanente Washington Health Research Institute, Seattle, WA, USA
113 Brain Center Rudolf Magnus, Department of Neurology, University Medical Center Utrecht, Utrecht, The Netherlands
114 Usher Institute of Population Health Sciences and Informatics, University of Edinburgh, Edinburgh, UK
115 Centre for Clinical Brain Sciences, University of Edinburgh, Edinburgh, UK
116 Fred Hutchinson Cancer Research Center, University of Washington, Seattle, WA, USA
117 Department of Medicine, Brigham and Women's Hospital, Boston, MA, USA
118 Department of Biostatistics, University of Washington, Seattle, WA, USA
119 Nuffield Department of Clinical Neurosciences, University of Oxford, UK
120 Institute for Translational Genomics and Population Sciences, Los Angeles Biomedical Research Institute at Harbor-UCLA Medical Center, Torrance, CA, USA
121 Division of Genomic Outcomes, Department of Pediatrics, Harbor-UCLA Medical Center, Torrance, CA, USA
122 Department of Neurology, Miller School of Medicine, University of Miami, Miami, FL, USA
123 Department of Allergy and Rheumatology, Graduate School of Medicine, the University of Tokyo, Tokyo, Japan
124 Center for Public Health Genomics, University of Virginia, Charlottesville, VA, USA
125 Department of Pediatrics, College of Medicine, University of Oklahoma Health Sciences Center, Oklahoma City, OK, USA
126 Department of Neurology, Medical University of Graz, Graz, Austria
127 University Medicine Greifswald, Institute for Community Medicine, SHIP-KEF, Greifswald, Germany
128 University Medicine Greifswald, Department of Neurology, Greifswald, Germany
129 Department of Neurology, Jagiellonian University, Krakow, Poland

130 Department of Neurology, Justus Liebig University, Giessen, Germany
131 Department of Clinical Neurosciences/Neurology, Institute of Neuroscience and Physiology, Sahlgrenska Academy at University of Gothenburg, Gothenburg, Sweden
132 Sahlgrenska University Hospital, Gothenburg, Sweden
133 Stroke Division, Florey Institute of Neuroscience and Mental Health, University of Melbourne, Heidelberg, Australia
134 Austin Health, Department of Neurology, Heidelberg, Australia
135 Department of Internal Medicine, Section Gerontology and Geriatrics, Leiden University Medical Center, Leiden, the Netherlands
136 INSERM U1219, Bordeaux, France
137 Department of Public Health, Bordeaux University Hospital, Bordeaux, France
138 Genetic Epidemiology Unit, Department of Epidemiology, Erasmus University Medical Center Rotterdam, Netherlands
139 Center for Medical Systems Biology, Leiden, Netherlands
140 School of Medicine, Dentistry and Nursing at the University of Glasgow, Glasgow, UK
141 Department of Epidemiology and Population Health, Albert Einstein College of Medicine, NY, USA
142 Department of Physiology and Biophysics, University of Mississippi Medical Center, Jackson, MS, USA
143 A full list of members and affiliations appears in the Supplementary Note
144 Department of Human Genetics, McGill University, Montreal, Canada
145 Department of Pathophysiology, Institute of Biomedicine and Translation Medicine, University of Tartu, Tartu, Estonia
146 Department of Cardiac Surgery, Tartu University Hospital, Tartu, Estonia
147 Clinical Gene Networks AB,Stockholm, Sweden
148 Department of Genetics and Genomic Sciences, The Icahn Institute for Genomics and Multiscale Biology Icahn School of Medicine at Mount Sinai, New York, NY , USA
149 Department of Pathophysiology, Institute of Biomedicine and Translation Medicine, University of Tartu, Biomeedikum, Tartu, Estonia
150 Integrated Cardio Metabolic Centre, Department of Medicine, Karolinska Institutet, Karolinska Universitetssjukhuset, Huddinge, Sweden.
151 Clinical Gene Networks AB, Stockholm, Sweden
152 Sorbonne Universités, UPMC Univ. Paris 06, INSERM, UMR_S 1166, Team Genomics & Pathophysiology of Cardiovascular Diseases, Paris, France
153 ICAN Institute for Cardiometabolism and Nutrition, Paris, France
154 Department of Biomedical Engineering, University of Virginia, Charlottesville, VA, USA
155 Group Health Research Institute, Group Health Cooperative, Seattle, WA, USA
156 Seattle Epidemiologic Research and Information Center, VA Office of Research and Development, Seattle, WA, USA
157 Cardiovascular Research Center, Massachusetts General Hospital, Boston, MA, USA
158 Department of Medical Research, Bærum Hospital, Vestre Viken Hospital Trust, Gjettum, Norway
159 Saw Swee Hock School of Public Health, National University of Singapore and National University Health System, Singapore
160 National Heart and Lung Institute, Imperial College London, London, UK
161 Department of Gene Diagnostics and Therapeutics, Research Institute, National Center for Global Health and Medicine, Tokyo, Japan
162 Department of Epidemiology, Tulane University School of Public Health and Tropical Medicine, New Orleans, LA, USA
163 Department of Cardiology,University Medical Center Groningen, University of Groningen, Netherlands
164 MRC-PHE Centre for Environment and Health, School of Public Health, Department of Epidemiology and Biostatistics, Imperial College London, London, UK
165 Department of Epidemiology and Biostatistics, Imperial College London, London, UK
166 Department of Cardiology, Ealing Hospital NHS Trust, Southall, UK
167 National Heart, Lung and Blood Research Institute, Division of Intramural Research, Population Sciences Branch, Framingham, MA, USA
168 A full list of members and affiliations appears at the end of the manuscript
169 Department of Phamaceutical Sciences, Collge of Pharmacy, University of Oklahoma Health Sciences Center, Oklahoma City, OK, USA
170 Oklahoma Center for Neuroscience, Oklahoma City, OK, USA
171 Department of Pathology and Genetics, Institute of Biomedicine, The Sahlgrenska Academy at University of Gothenburg, Gothenburg, Sweden
172 Department of Neurology, Helsinki University Hospital, Helsinki, Finland
173 Clinical Neurosciences, Neurology, University of Helsinki, Helsinki, Finland
174 Department of Neurology, University of Washington, Seattle, WA, USA
175 Albrecht Kossel Institute, University Clinic of Rostock, Rostock, Germany
176 Clinical Trial Service Unit and Epidemiological Studies Unit, Nuffield Department of Population Health, University of Oxford, Oxford, UK
177 Department of Genetics, Perelman School of Medicine, University of Pennsylvania, PA, USA
178 Faculty of Medicine, University of Iceland, Reykjavik, Iceland
179 Departments of Neurology and Public Health Sciences, University of Virginia School of Medicine, Charlottesville, VA, USA
180 Department of Neurology, Boston University School of Medicine, Boston, MA, USA
181 Human Genetics Center, University of Texas Health Science Center at Houston, Houston, TX, USA
182 Center for Genomic Medicine, Kyoto University Graduate School of Medicine, Kyoto, Japan
183 Munich Cluster for Systems Neurology (SyNergy), Munich, Germany
184 German Center for Neurodegenerative Diseases (DZNE), Munich, Germany
185 Boston University School of Medicine, Boston, MA, USA
186 University of Kentucky College of Public Health, Lexington, KY, USA
187 University of Newcastle and Hunter Medical Research Institute, New Lambton, Australia
188 Univ. Montpellier, Inserm, U1061, Montpellier, France
189 Centre for Research in Environmental Epidemiology, Barcelona, Spain
190 Department of Neurology, Università degli Studi di Perugia, Umbria, Italy
191 Department of Medicine, University of Maryland School of Medicine, Baltimore, MD, USA
192 Broad Institute, Cambridge, MA, USA
193 Univ. Bordeaux, Inserm, Bordeaux Population Health Research Center, UMR 1219, Bordeaux, France
194 Bordeaux University Hospital, Department of Neurology, Memory Clinic, Bordeaux, France
195 Neurovascular Research Laboratory. Vall d'Hebron Institut of Research, Neurology and Medicine Departments-Universitat Autònoma de Barcelona. Vall d'Hebrón Hospital, Barcelona, Spain
196 University Medicine Greifswald, Department of Internal Medicine B, Greifswald, Germany
197 DZHK, Greifswald, Germany
198 Robertson Center for Biostatistics, University of Glasgow, Glasgow, UK
199 Hero DMC Heart Institute, Dayanand Medical College & Hospital, Ludhiana, India
200 Atherosclerosis Research Unit, Department of Medicine Solna, Karolinska Institutet, Stockholm, Sweden
201 Karolinska Institutet, Stockholm, Sweden
202 Division of Emergency Medicine, and Department of Neurology, Washington University School of Medicine, St. Louis, MO, USA
203 Tohoku Medical Megabank Organization, Sendai, Japan
204 Department of Psychiatry, Washington University School of Medicine, St. Louis, MO, USA
205 Department of Public Health and Caring Sciences / Geriatrics, Uppsala University, Uppsala, Sweden
206 Epidemiology and Prevention Group, Center for Public Health Sciences, National Cancer Center, Tokyo, Japan

207 Department of Internal Medicine and the Center for Clinical and Translational Science, The Ohio State University, Columbus, OH, USA
208 Institute of Neuroscience and Physiology, the Sahlgrenska Academy at University of Gothenburg, Goteborg, Sweden
209 Department of Basic and Clinical Neurosciences, King's College London, London, UK
210 Department of Health Care Administration and Management, Graduate School of Medical Sciences, Kyushu University, Japan
211 Department of Medicine and Clinical Science, Graduate School of Medical Sciences, Kyushu University, Japan
212 Landspitali National University Hospital, Departments of Neurology & Radiology, Reykjavik, Iceland
213 Department of Neurology, Heidelberg University Hospital, Germany
214 Department of Neurology, Erasmus University Medical Center
215 Hospital Universitari Mutua Terrassa, Terrassa (Barcelona), Spain
216 Albert Einstein College of Medicine, Montefiore Medical Center, New York, NY, USA
217 John Hunter Hospital, Hunter Medical Research Institute and University of Newcastle, Newcastle, NSW, Australia
218 Centre for Prevention of Stroke and Dementia, Nuffield Department of Clinical Neurosciences, University of Oxford, UK
219 Department of Medical Sciences, Uppsala University, Uppsala, Sweden
220 Genetic and Genomic Epidemiology Unit, Wellcome Trust Centre for Human Genetics, University of Oxford, Oxford, UK
221 The Wellcome Trust Centre for Human Genetics, Oxford, UK
222 Beth Israel Deaconess Medical Center, Boston, MA, USA
223 Wake Forest School of Medicine, Wake Forest, NC, USA
224 Department of Neurology, University of Pittsburgh, Pittsburgh, PA, USA
225 BioBank Japan, Laboratory of Clinical Sequencing, Department of Computational biology and medical Sciences, Graduate school of Frontier Sciences, The University of Tokyo, Tokyo, Japan
226 Neurovascular Research Laboratory, Vall d'Hebron Institut of Research, Neurology and Medicine Departments-Universitat Autònoma de Barcelona. Vall d'Hebrón Hospital, Barcelona, Spain
227 Department of Biostatistics, University of Liverpool, Liverpool, UK
228 Wellcome Trust Centre for Human Genetics, University of Oxford, Oxford, UK
229 Institute of Genetic Epidemiology, Helmholtz Zentrum München - German Research Center for Environmental Health, Neuherberg, Germany
230 Department of Medicine I, Ludwig-Maximilians-Universität, Munich, Germany
231 DZHK (German Centre for Cardiovascular Research), partner site Munich Heart Alliance, Munich, Germany
232 Department of Cerebrovascular Diseases, Fondazione IRCCS Istituto Neurologico "Carlo Besta", Milano, Italy
233 Karolinska Institutet, MEB, Stockholm, Sweden
234 University of Tartu, Estonian Genome Center, Tartu, Estonia, Tartu, Estonia
235 Department of Clinical and Experimental Sciences, Neurology Clinic, University of Brescia, Italy
236 Translational Genomics Unit, Department of Oncology, IRCCS Istituto di Ricerche Farmacologiche Mario Negri, Milano, Italy
237 Department of Genetics, Microbiology and Statistics, University of Barcelona, Barcelona, Spain
238 Psychiatric Genetics Unit, Group of Psychiatry, Mental Health and Addictions, Vall d'Hebron Research Institute (VHIR), Universitat Autònoma de Barcelona, Biomedical Network Research Centre on Mental Health (CIBERSAM), Barcelona, Spain
239 Department of Neurology, IMIM-Hospital del Mar, and Universitat Autònoma de Barcelona, Spain
240 IMIM (Hospital del Mar Medical Research Institute), Barcelona, Spain
241 National Institute for Health Research Comprehensive Biomedical Research Centre, Guy's & St. Thomas' NHS Foundation Trust and King's College London, London, UK
242 Division of Health and Social Care Research, King's College London, London, UK
243 FIMM-Institute for Molecular Medicine Finland, Helsinki, Finland
244 THL-National Institute for Health and Welfare, Helsinki, Finland
245 Iwate Tohoku Medical Megabank Organization, Iwate Medical University, Iwate, Japan
246 BHF Glasgow Cardiovascular Research Centre, Faculty of Medicine, Glasgow, UK
247 deCODE Genetics/Amgen, Inc., Reykjavik, Iceland
248 Icelandic Heart Association, Reykjavik, Iceland
249 Institute of Biomedicine, the Sahlgrenska Academy at University of Gothenburg, Goteborg, Sweden
250 Department of Epidemiology, University of Maryland School of Medicine, Baltimore, MD, USA
251 Institute of Cardiovascular and Medical Sciences, Faculty of Medicine, University of Glasgow, Glasgow, UK
252 Chair of Genetic Epidemiology, IBE, Faculty of Medicine, LMU Munich, Germany
253 Division of Epidemiology and Prevention, Aichi Cancer Center Research Institute, Nagoya, Japan
254 Department of Epidemiology, Nagoya University Graduate School of Medicine, Nagoya, Japan
255 University Medicine Greifswald, Institute for Community Medicine, SHIP-KEF, Greifswald, Germany
256 Department of Neurology, Caen University Hospital, Caen, France
257 University of Caen Normandy, Caen, France
258 Department of Internal Medicine, Erasmus University Medical Center, Rotterdam, Netherlands
259 Landspitali University Hospital, Reykjavik, Iceland
260 Survey Research Center, University of Michigan, Ann Arbor, MI, USA
261 University of Virginia Department of Neurology, Charlottesville, VA, USA

Supplementary Figure 1: Workflow of the project

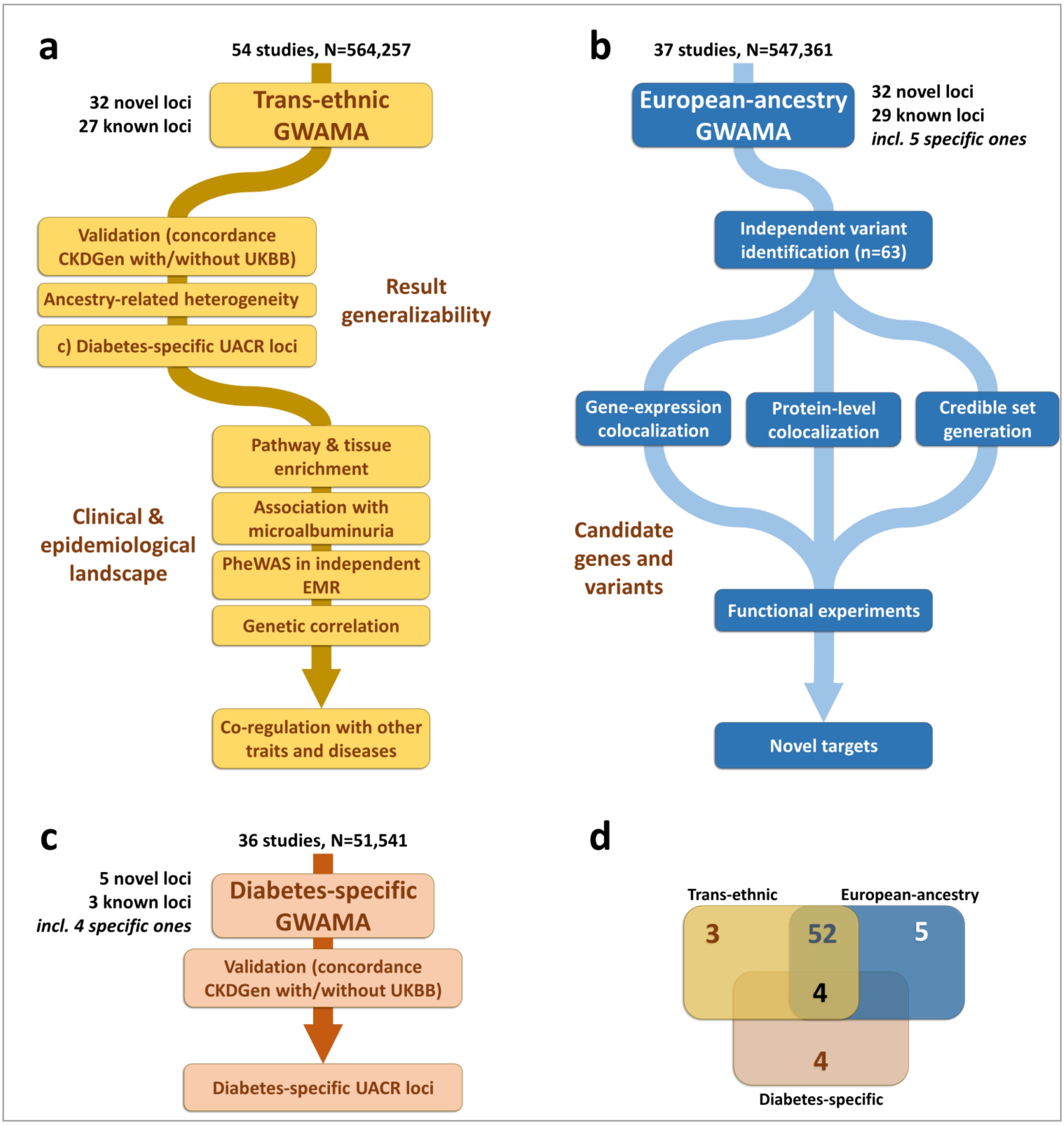

Overview of the analysis workflow for trans-ethnic (a), European ancestry-specific (b), and diabetes-specific (c) genome-wide association meta-analyses (GWAMA). The Venn diagram in panel (d) shows the number of overlapping loci between the different GWAMA.

Supplementary Figure 2: Regional association plots for loci identified in trans-ethnic GWAS meta-analysis of UACR

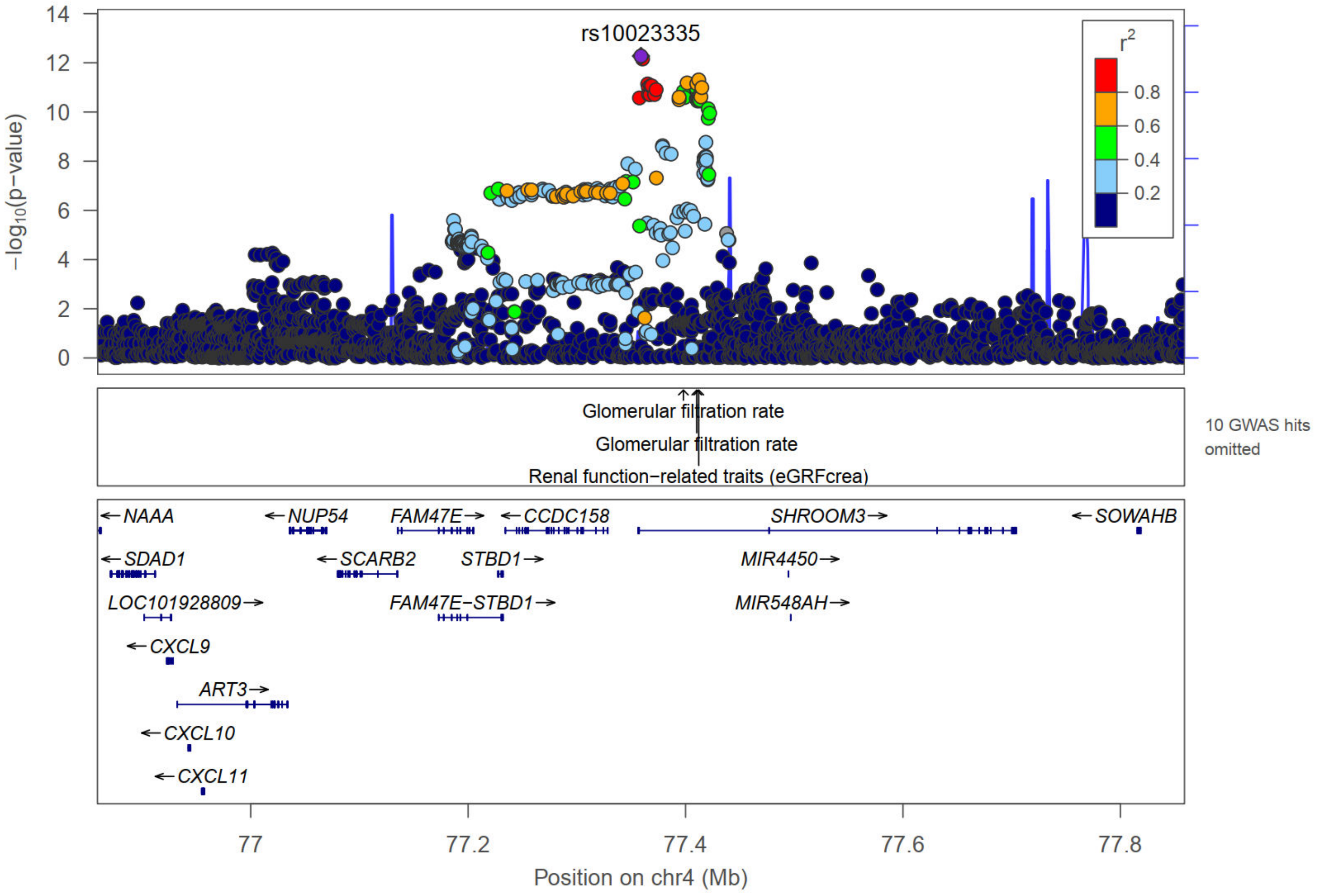

rs10023335
−log10(p−value)
r2
0.8
0.6
0.4
0.2
Glomerular filtration rate
Glomerular filtration rate
Renal function−related traits (eGRFcrea)
10 GWAS hits omitted
NAAA
NUP54
FAM47E
CCDC158
SHROOM3
SOWAHB
SDAD1
SCARB2
STBD1
MIR4450
LOC101928809
FAM47E−STBD1
MIR548AH
CXCL9
ART3
CXCL10
CXCL11
Position on chr4 (Mb)

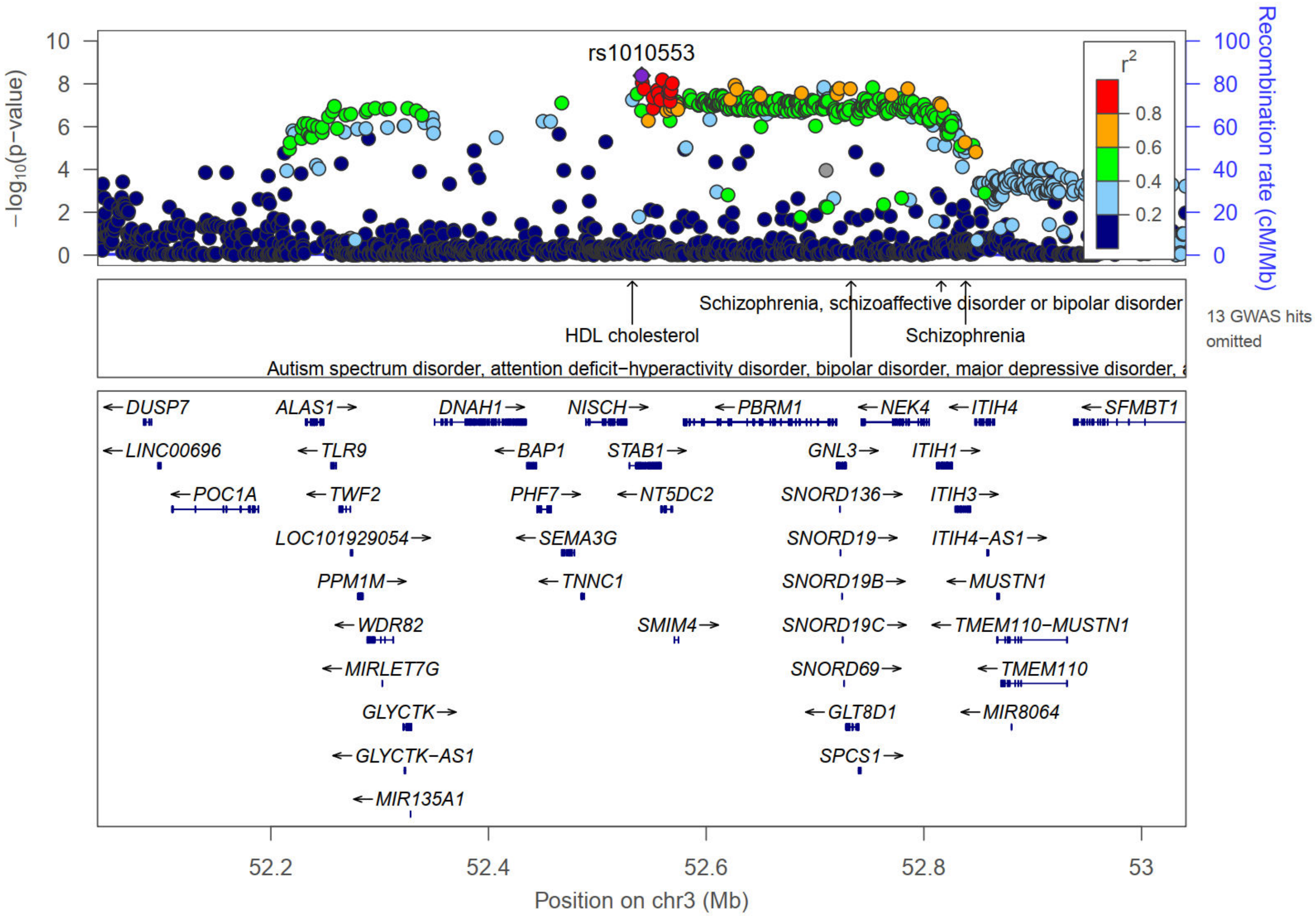

rs1010553
−log10(p−value)
Recombination rate (cM/Mb)
r2
0.8
0.6
0.4
0.2
10
8
6
4
2
0
100
80
60
40
20
HDL cholesterol
Schizophrenia, schizoaffective disorder or bipolar disorder
Schizophrenia
Autism spectrum disorder, attention deficit−hyperactivity disorder, bipolar disorder, major depressive disorder,
13 GWAS hits omitted
DUSP7
LINC00696
POC1A
ALAS1
TLR9
TWF2
LOC101929054
PPM1M
WDR82
MIRLET7G
GLYCTK
GLYCTK−AS1
MIR135A1
DNAH1
BAP1
PHF7
SEMA3G
TNNC1
NISCH
STAB1
NT5DC2
SMIM4
PBRM1
GNL3
SNORD136
SNORD19
SNORD19B
SNORD19C
SNORD69
GLT8D1
SPCS1
NEK4
ITIH1
ITIH3
ITIH4−AS1
ITIH4
MUSTN1
TMEM110−MUSTN1
TMEM110
MIR8064
SFMBT1
52.2
52.4
52.6
52.8
53
Position on chr3 (Mb)

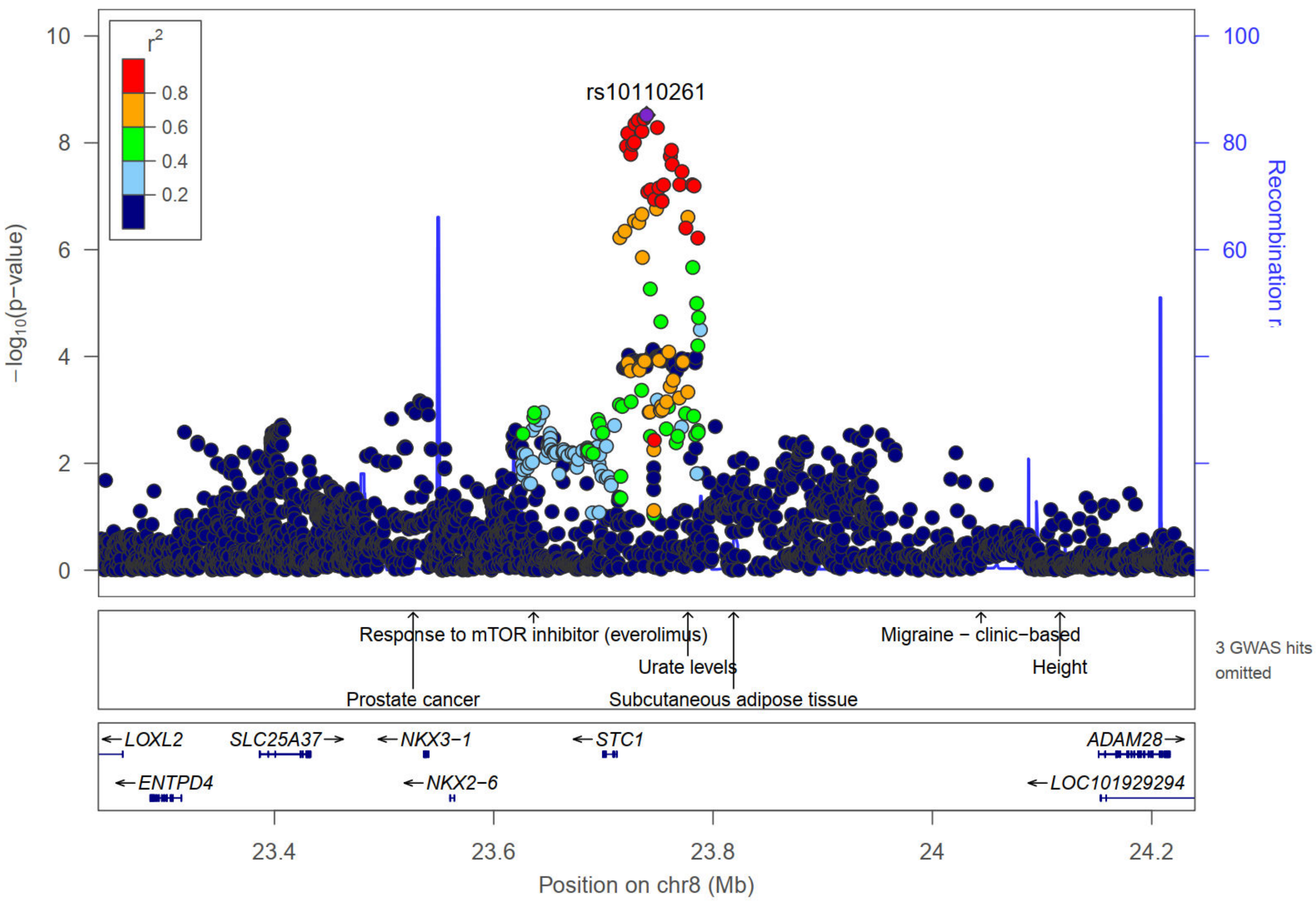

rs10110261
r2
0.8
0.6
0.4
0.2
−log10(p−value)
Recombination r.
Response to mTOR inhibitor (everolimus)
Prostate cancer
Urate levels
Subcutaneous adipose tissue
Migraine − clinic−based
Height
3 GWAS hits omitted
LOXL2
SLC25A37
NKX3−1
STC1
ADAM28
ENTPD4
NKX2−6
LOC101929294
Position on chr8 (Mb)

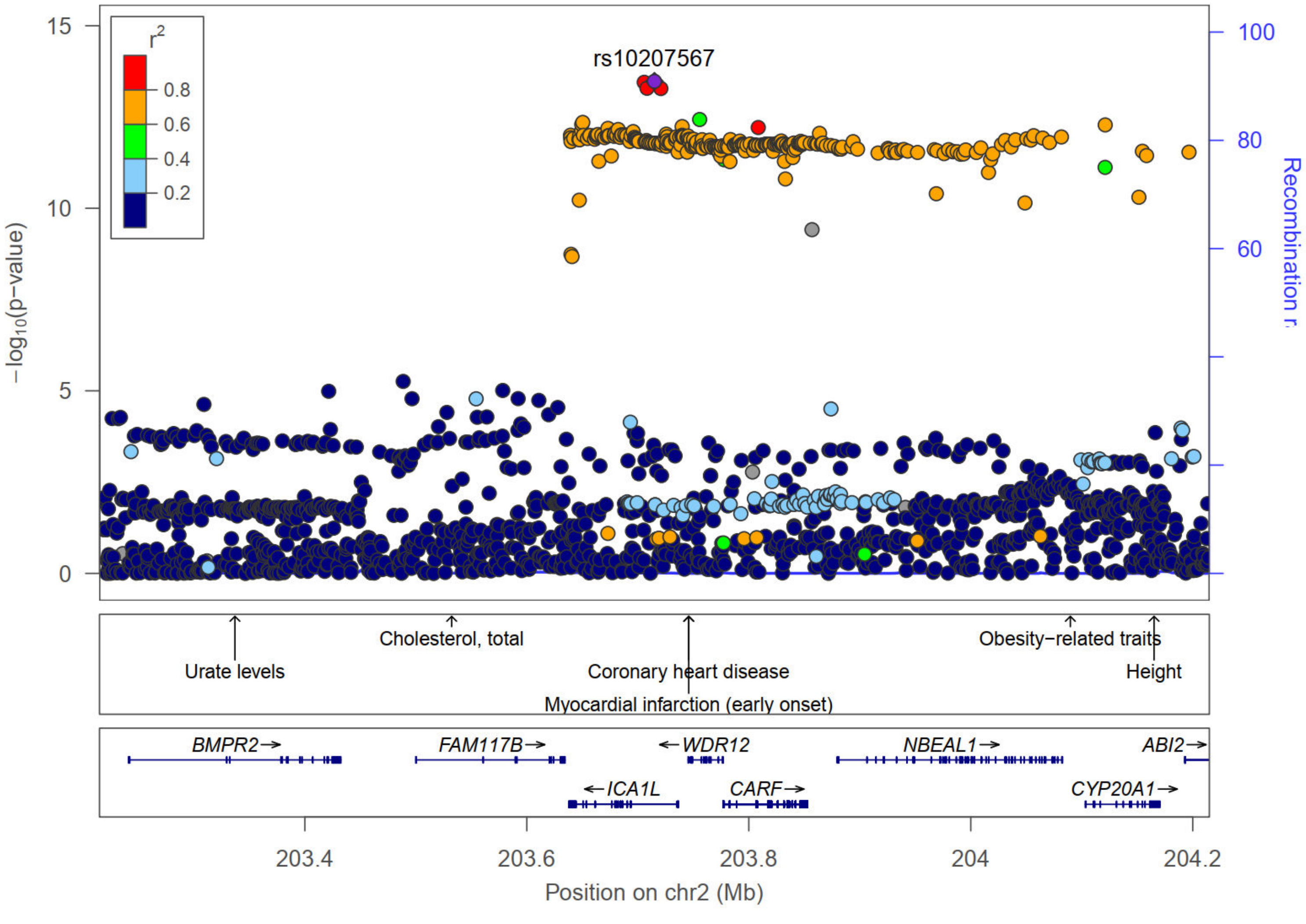

rs10207567
r2
0.8
0.6
0.4
0.2
15
10
5
0
−log10(p−value)
100
80
60
Recombination r
Urate levels
Cholesterol, total
Coronary heart disease
Myocardial infarction (early onset)
Obesity−related traits
Height
BMPR2→
FAM117B→
←WDR12
NBEAL1→
ABI2→
←ICA1L
CARF→
CYP20A1→
203.4
203.6
203.8
204
204.2
Position on chr2 (Mb)

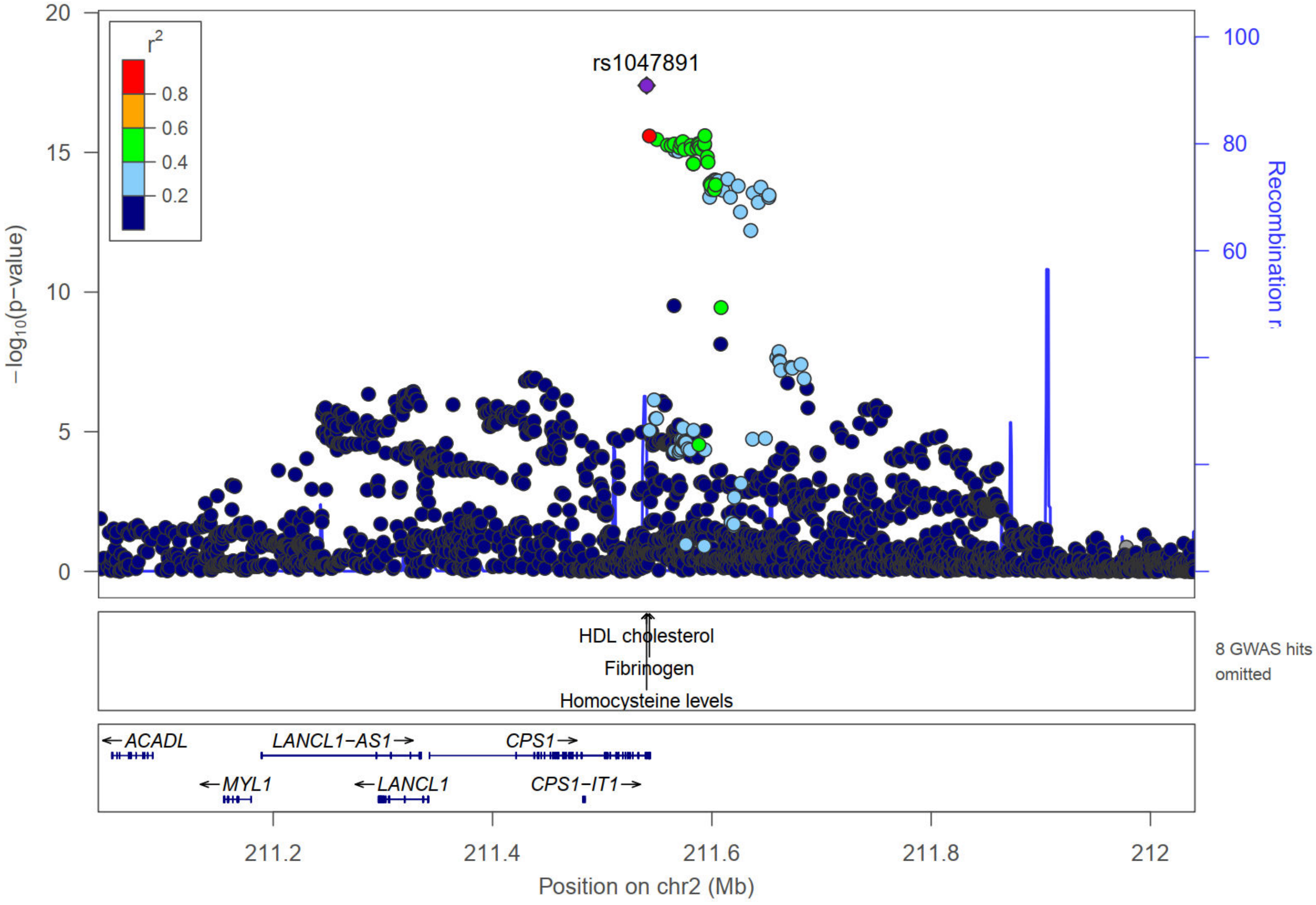

rs1047891
r2
0.8
0.6
0.4
0.2
20
15
10
5
0
−log10(p−value)
100
80
60
Recombination r
HDL cholesterol
Fibrinogen
Homocysteine levels
8 GWAS hits omitted
←ACADL
LANCL1−AS1→
CPS1→
←MYL1
←LANCL1
CPS1−IT1→
211.2
211.4
211.6
211.8
212
Position on chr2 (Mb)

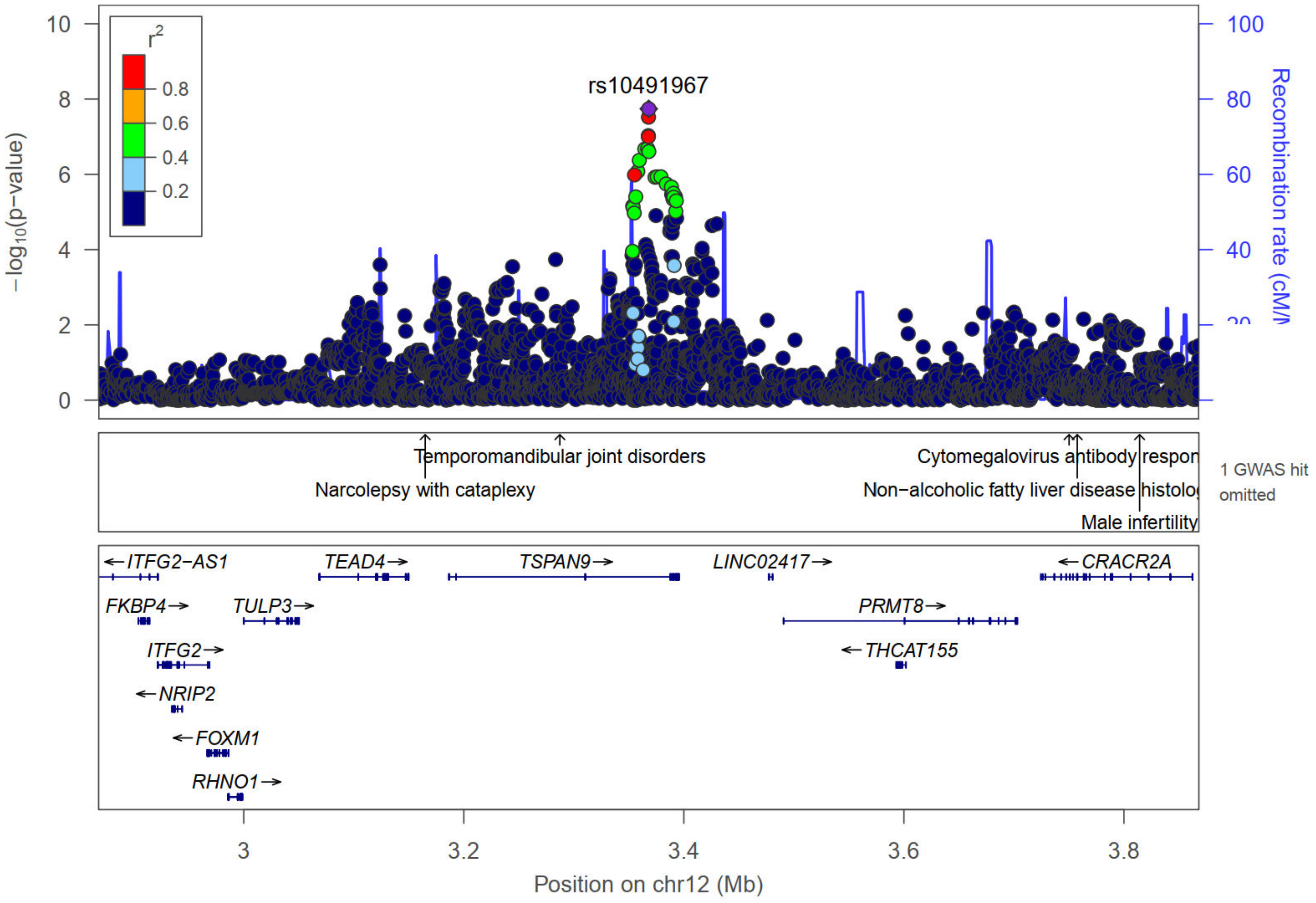

rs10491967
r2
0.8
0.6
0.4
0.2
−log10(p−value)
Recombination rate (cM/Mb)
Temporomandibular joint disorders
Narcolepsy with cataplexy
Cytomegalovirus antibody respon
Non−alcoholic fatty liver disease histolog
Male infertility
1 GWAS hit omitted
ITFG2−AS1
TEAD4
TSPAN9
LINC02417
CRACR2A
FKBP4
TULP3
PRMT8
ITFG2
THCAT155
NRIP2
FOXM1
RHNO1
Position on chr12 (Mb)

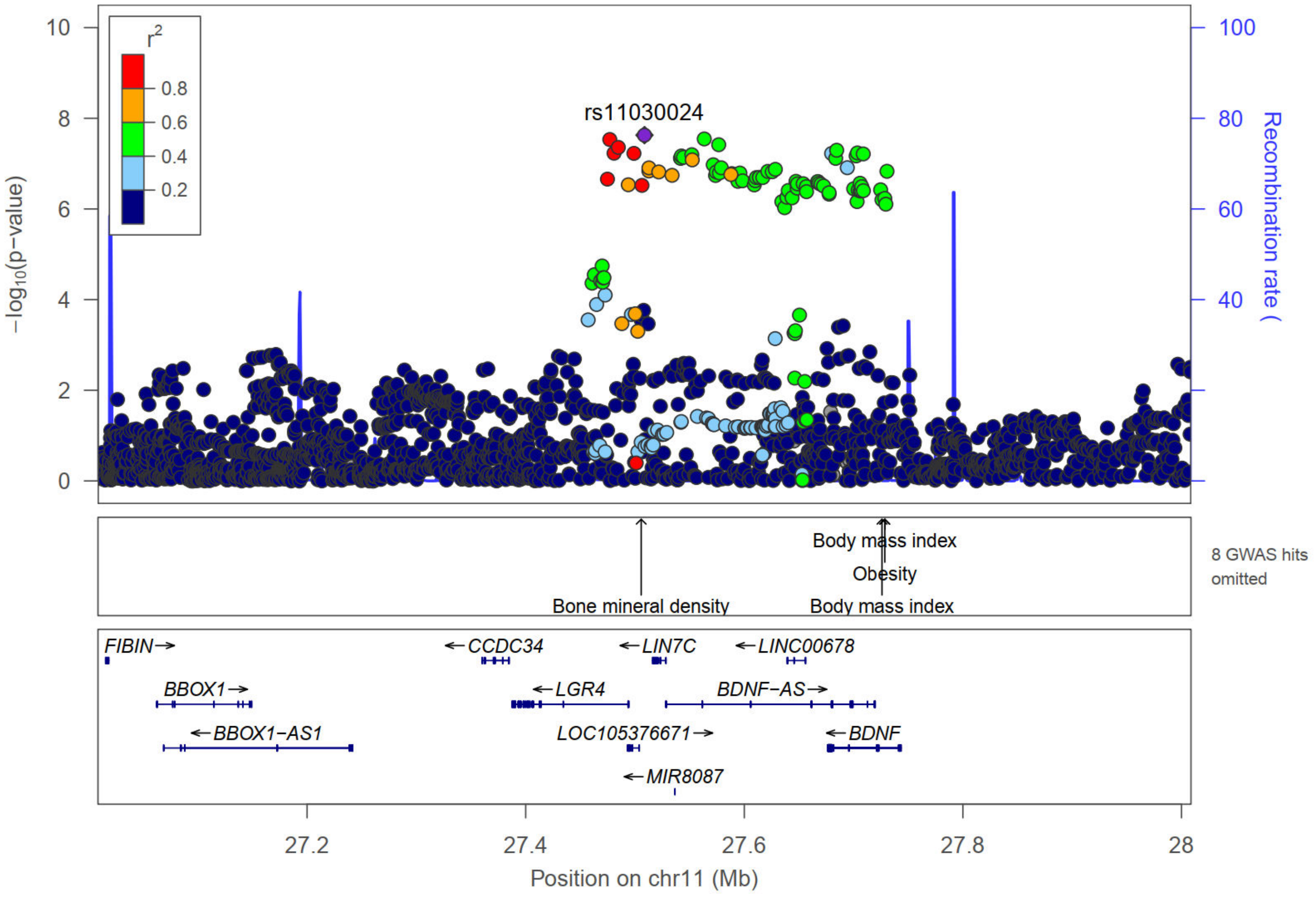

r2
0.8
0.6
0.4
0.2
rs11030024
−log10(p−value)
Recombination rate (
Body mass index
Obesity
Bone mineral density
Body mass index
8 GWAS hits omitted
FIBIN→
←CCDC34
←LIN7C
←LINC00678
BBOX1→
←LGR4
BDNF−AS→
←BBOX1−AS1
LOC105376671→
←BDNF
←MIR8087
27.2
27.4
27.6
27.8
28
Position on chr11 (Mb)

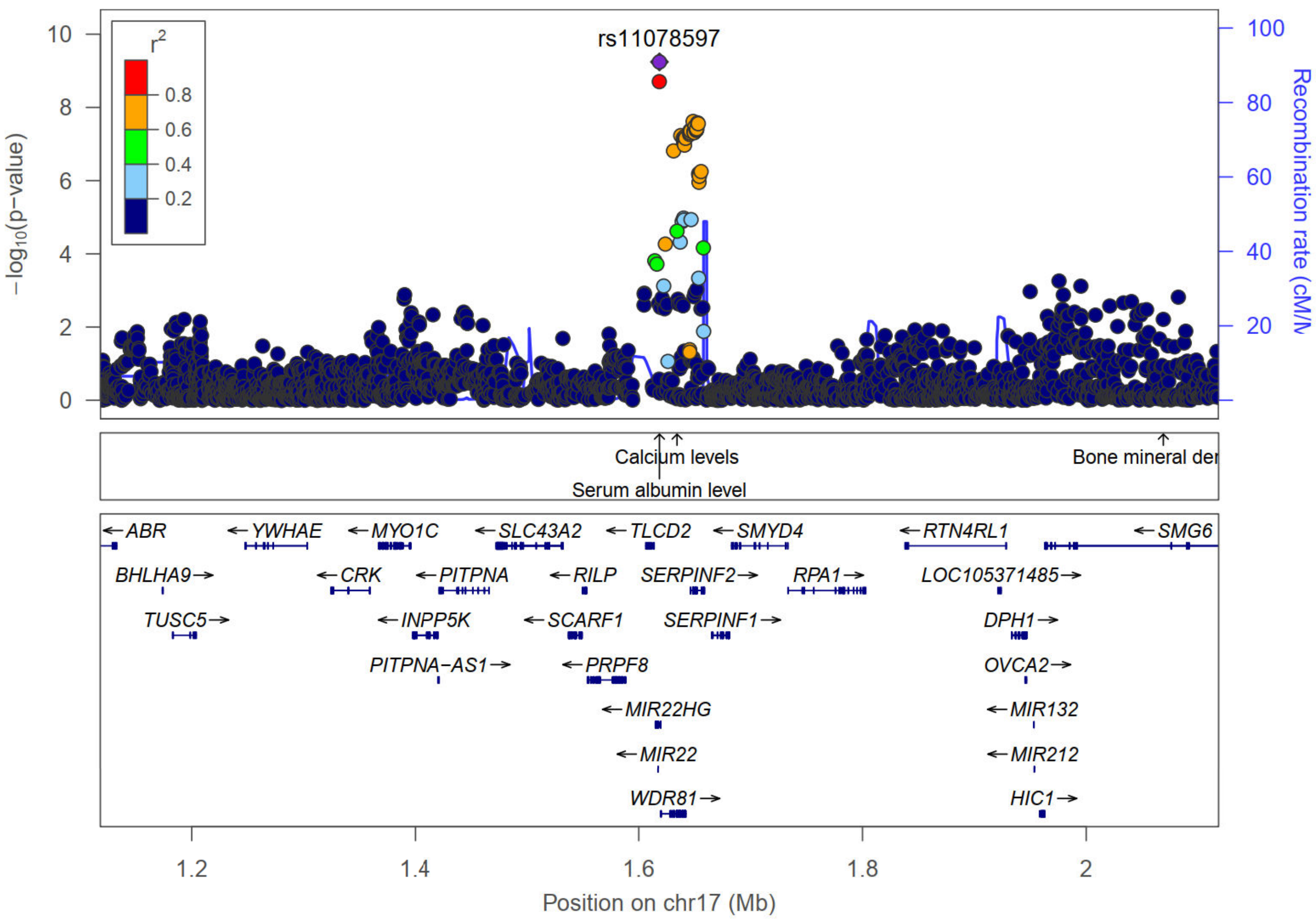

rs11078597
r2
0.8
0.6
0.4
0.2
−log10(p−value)
Recombination rate (cM/Mb)
Calcium levels
Serum albumin level
Bone mineral den
ABR
YWHAE
MYO1C
SLC43A2
TLCD2
SMYD4
RTN4RL1
SMG6
BHLHA9
CRK
PITPNA
RILP
SERPINF2
RPA1
LOC105371485
TUSC5
INPP5K
SCARF1
SERPINF1
DPH1
PITPNA−AS1
PRPF8
OVCA2
MIR22HG
MIR132
MIR22
MIR212
WDR81
HIC1
Position on chr17 (Mb)

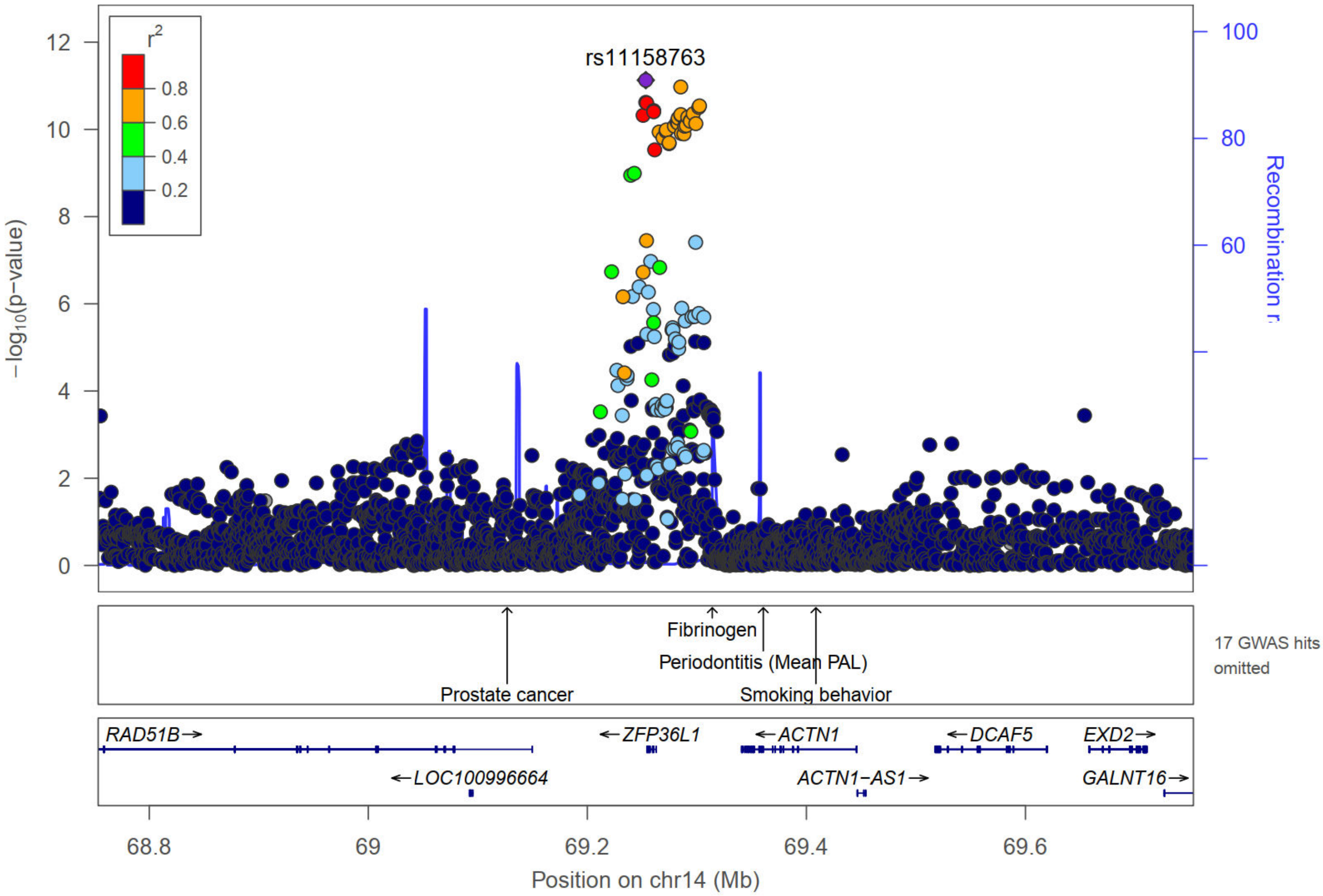

rs11158763
r2
0.8
0.6
0.4
0.2
−log10(p−value)
Recombination r
Fibrinogen
Periodontitis (Mean PAL)
Prostate cancer
Smoking behavior
17 GWAS hits omitted
RAD51B→
←ZFP36L1
←ACTN1
←DCAF5
EXD2→
←LOC100996664
ACTN1−AS1→
GALNT16→
68.8
69
69.2
69.4
69.6
Position on chr14 (Mb)

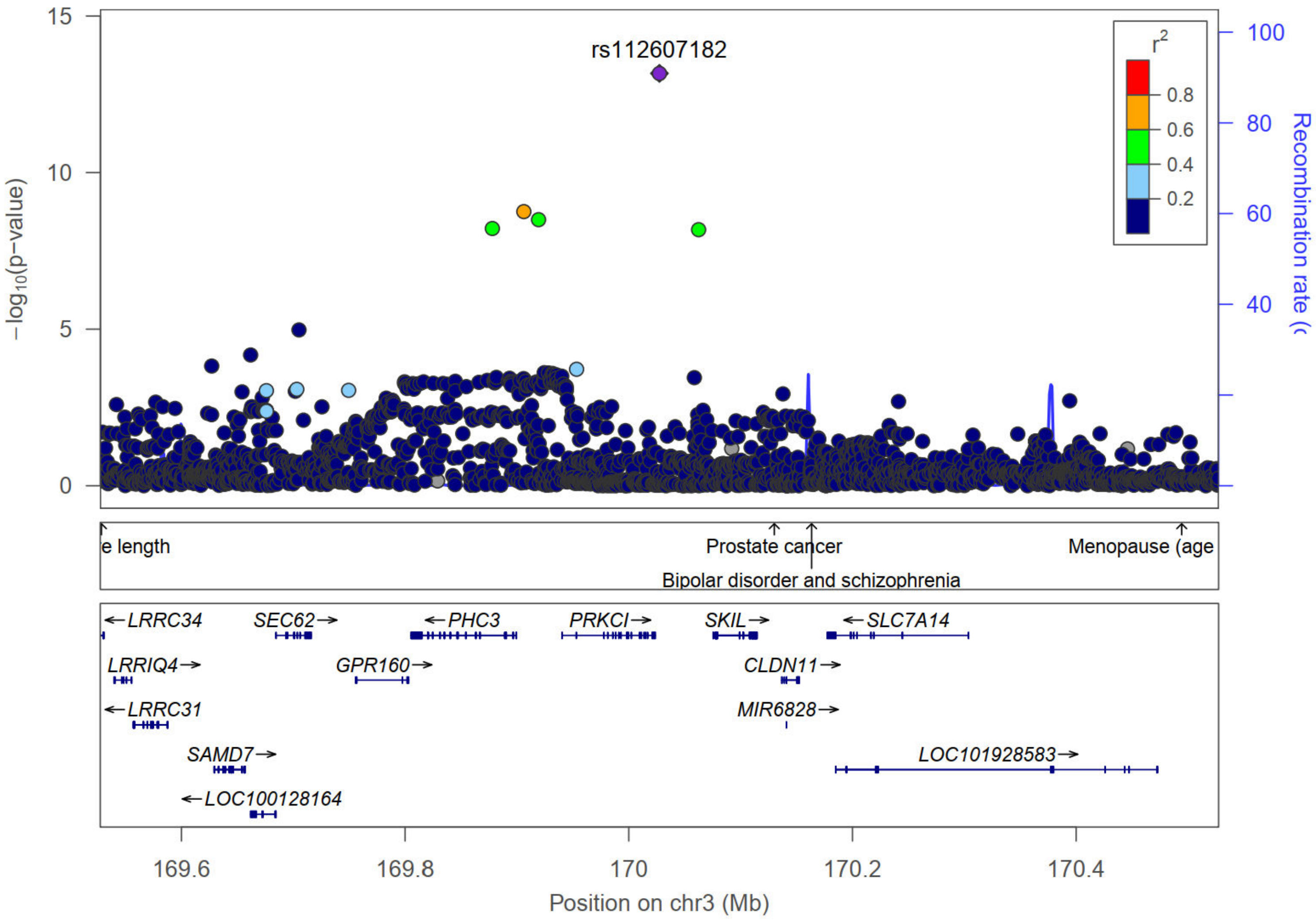
rs112607182
15
10
5
0
−log10(p−value)
100
80
60
40
Recombination rate (c
r2
0.8
0.6
0.4
0.2
e length
Prostate cancer
Menopause (age
Bipolar disorder and schizophrenia
LRRC34
SEC62
PHC3
PRKCI
SKIL
SLC7A14
LRRIQ4
GPR160
CLDN11
LRRC31
MIR6828
SAMD7
LOC101928583
LOC100128164
169.6
169.8
170
170.2
170.4
Position on chr3 (Mb)

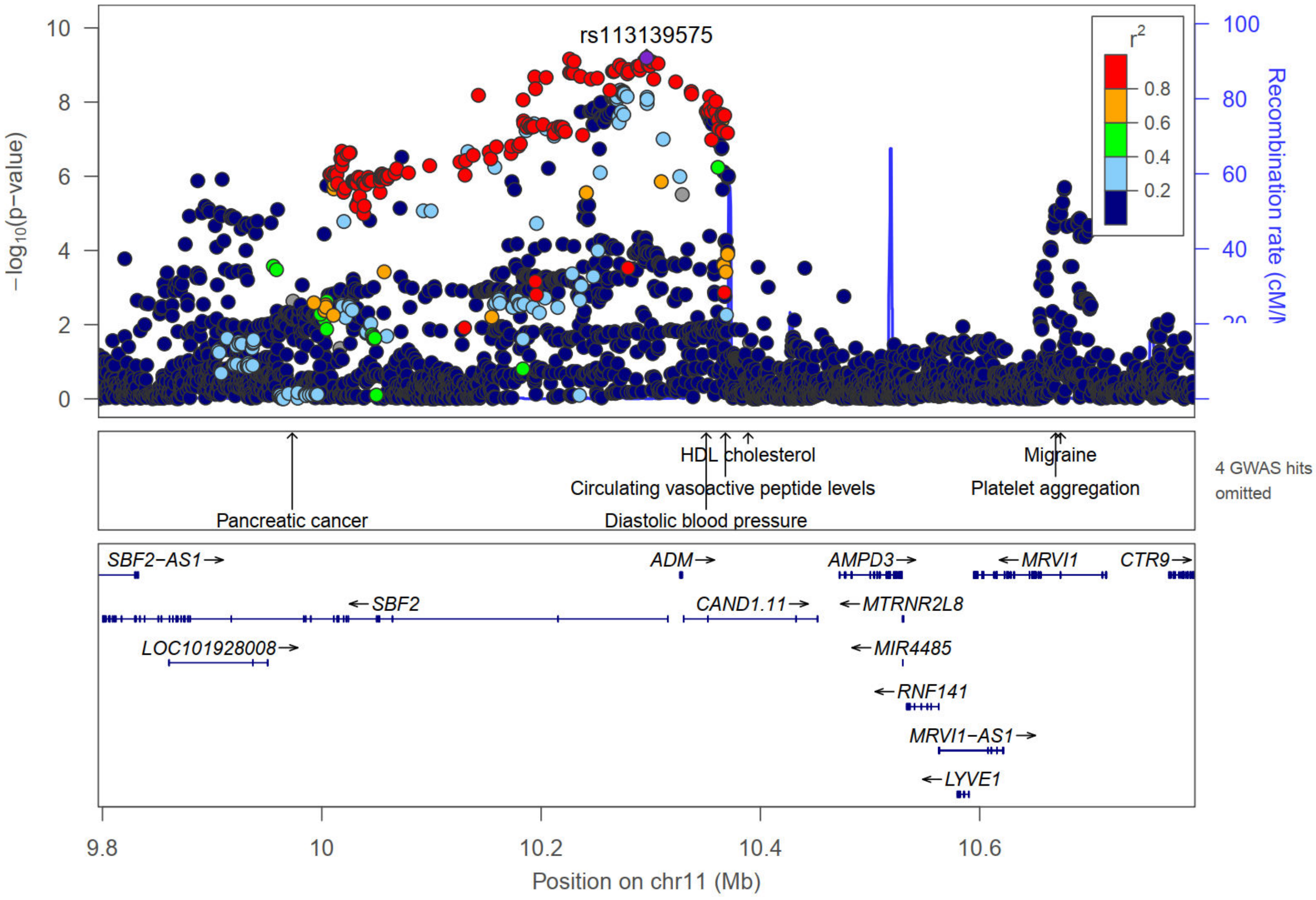

rs113139575
−log10(p−value)
Recombination rate (cM/Mb)
r2
0.8
0.6
0.4
0.2
Pancreatic cancer
HDL cholesterol
Circulating vasoactive peptide levels
Diastolic blood pressure
Migraine
Platelet aggregation
4 GWAS hits omitted
SBF2−AS1→
←SBF2
LOC101928008→
ADM→
CAND1.11→
AMPD3→
←MTRNR2L8
←MIR4485
←RNF141
MRVI1−AS1→
←LYVE1
←MRVI1
CTR9→
Position on chr11 (Mb)

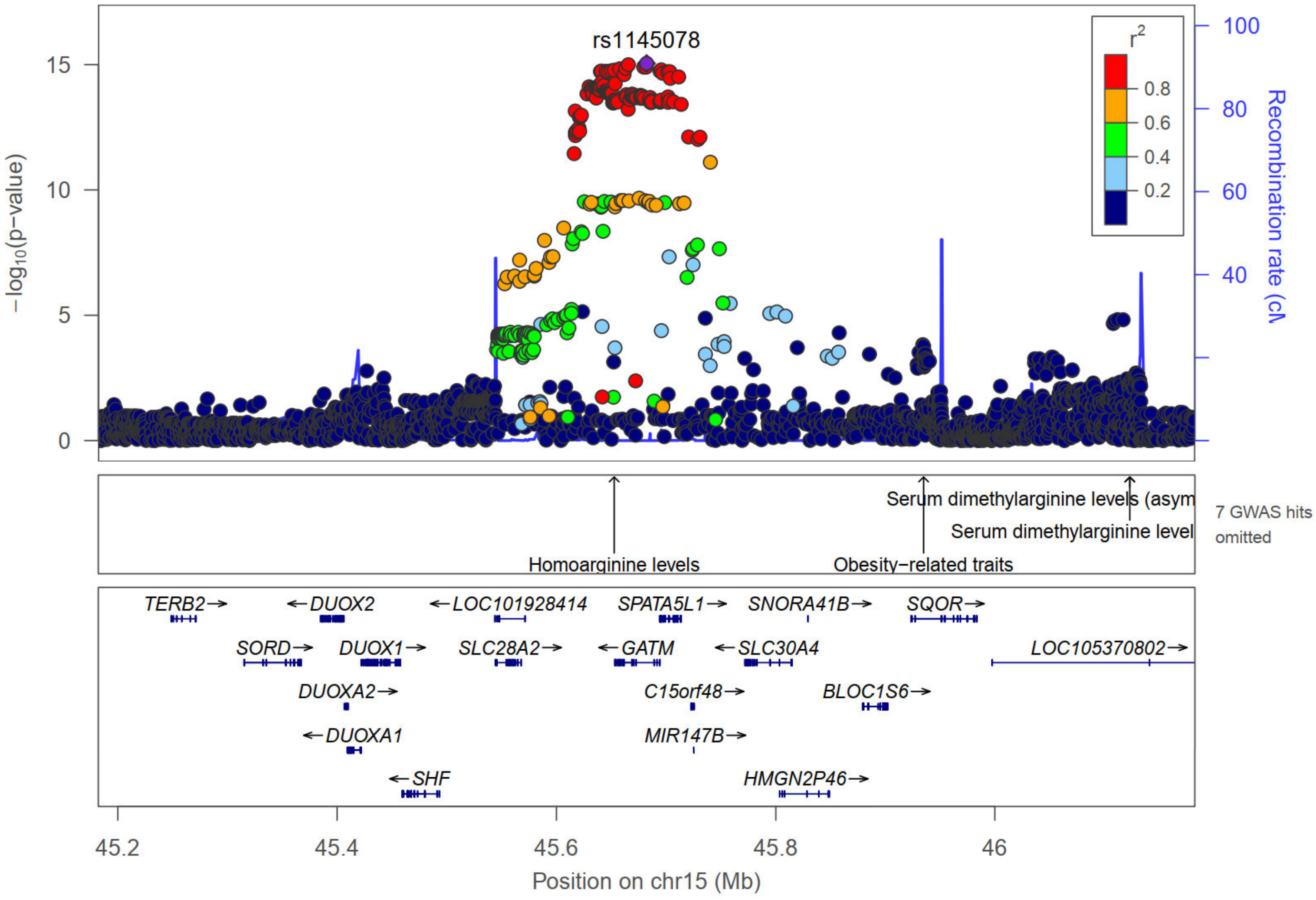

rs1145078
−log10(p−value)
Recombination rate (cM
r2
0.8
0.6
0.4
0.2
Homoarginine levels
Obesity−related traits
Serum dimethylarginine levels (asym
Serum dimethylarginine level
7 GWAS hits omitted
TERB2→
←DUOX2
←LOC101928414
SPATA5L1→
SNORA41B→
SQOR→
SORD→
DUOX1→
SLC28A2→
←GATM
←SLC30A4
LOC105370802→
DUOXA2→
C15orf48→
BLOC1S6→
←DUOXA1
MIR147B→
←SHF
HMGN2P46→
45.2
45.4
45.6
45.8
46
Position on chr15 (Mb)

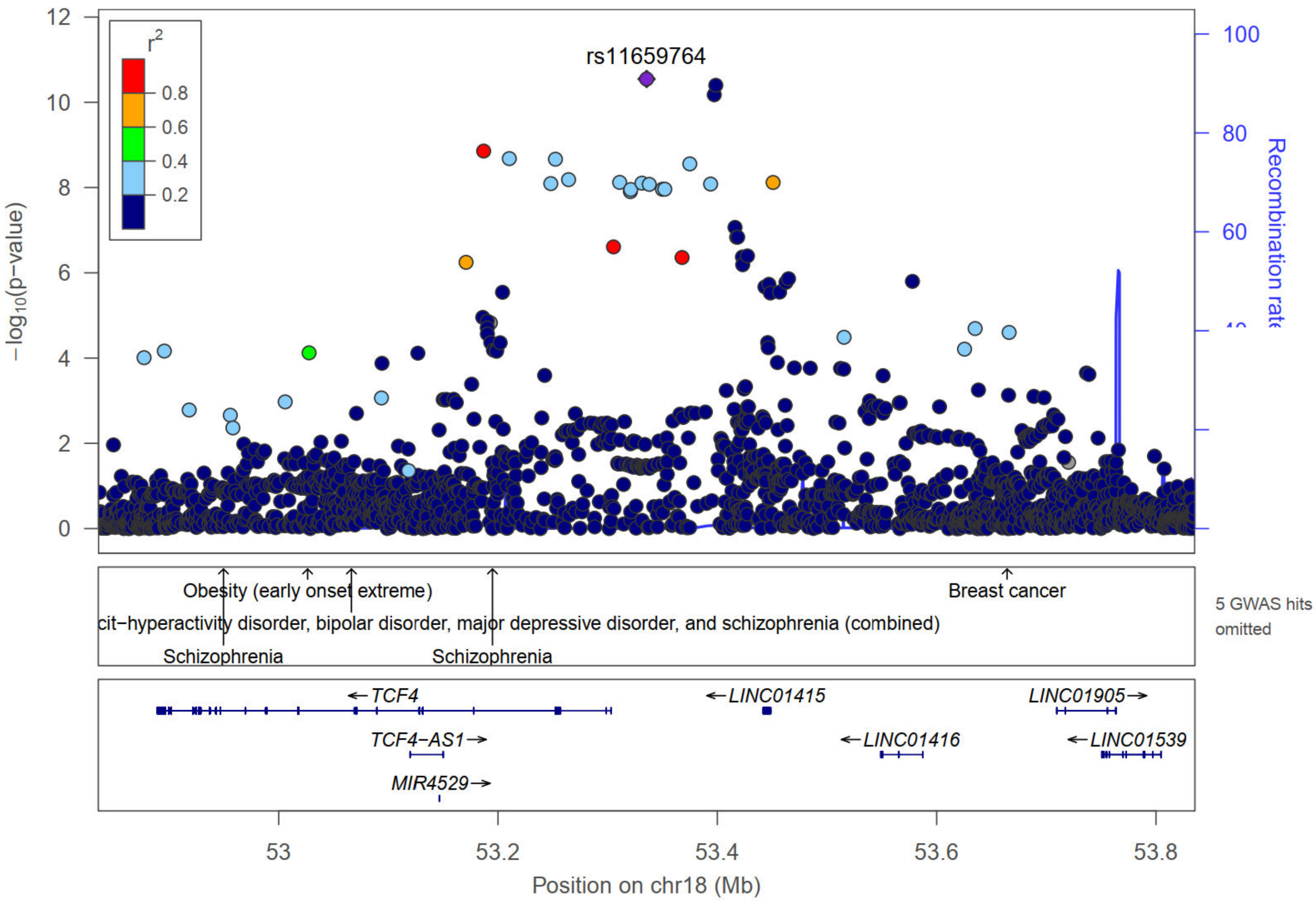
rs11659764
r2
0.8
0.6
0.4
0.2
−log10(p−value)
Recombination rate
Obesity (early onset extreme)
cit−hyperactivity disorder, bipolar disorder, major depressive disorder, and schizophrenia (combined)
Schizophrenia
Schizophrenia
Breast cancer
5 GWAS hits omitted
←TCF4
TCF4−AS1→
MIR4529→
←LINC01415
←LINC01416
LINC01905→
←LINC01539
Position on chr18 (Mb)

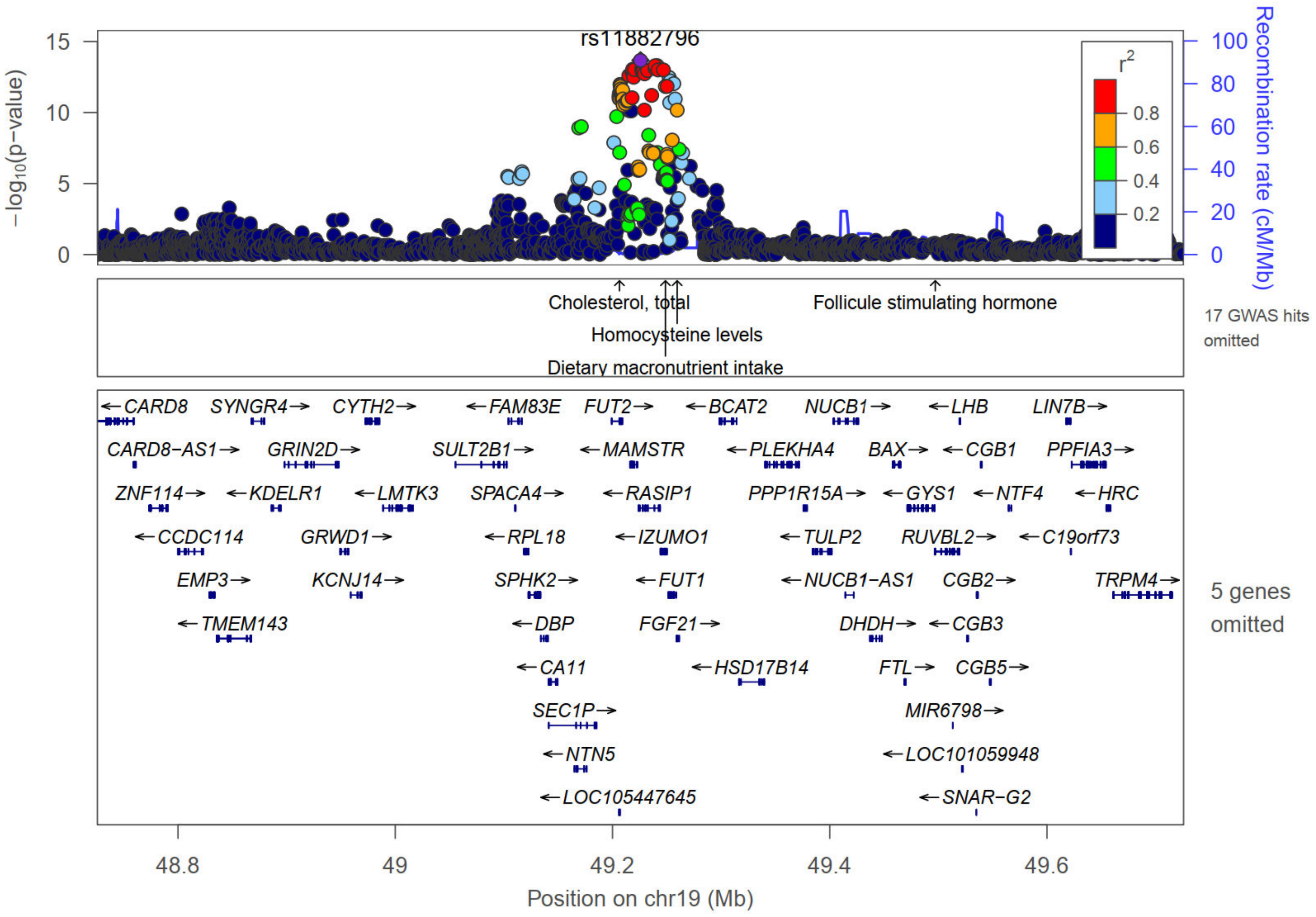

rs11882796
−log10(p−value)
Recombination rate (cM/Mb)
r2
Cholesterol, total
Homocysteine levels
Dietary macronutrient intake
Follicule stimulating hormone
17 GWAS hits omitted
5 genes omitted
Position on chr19 (Mb)

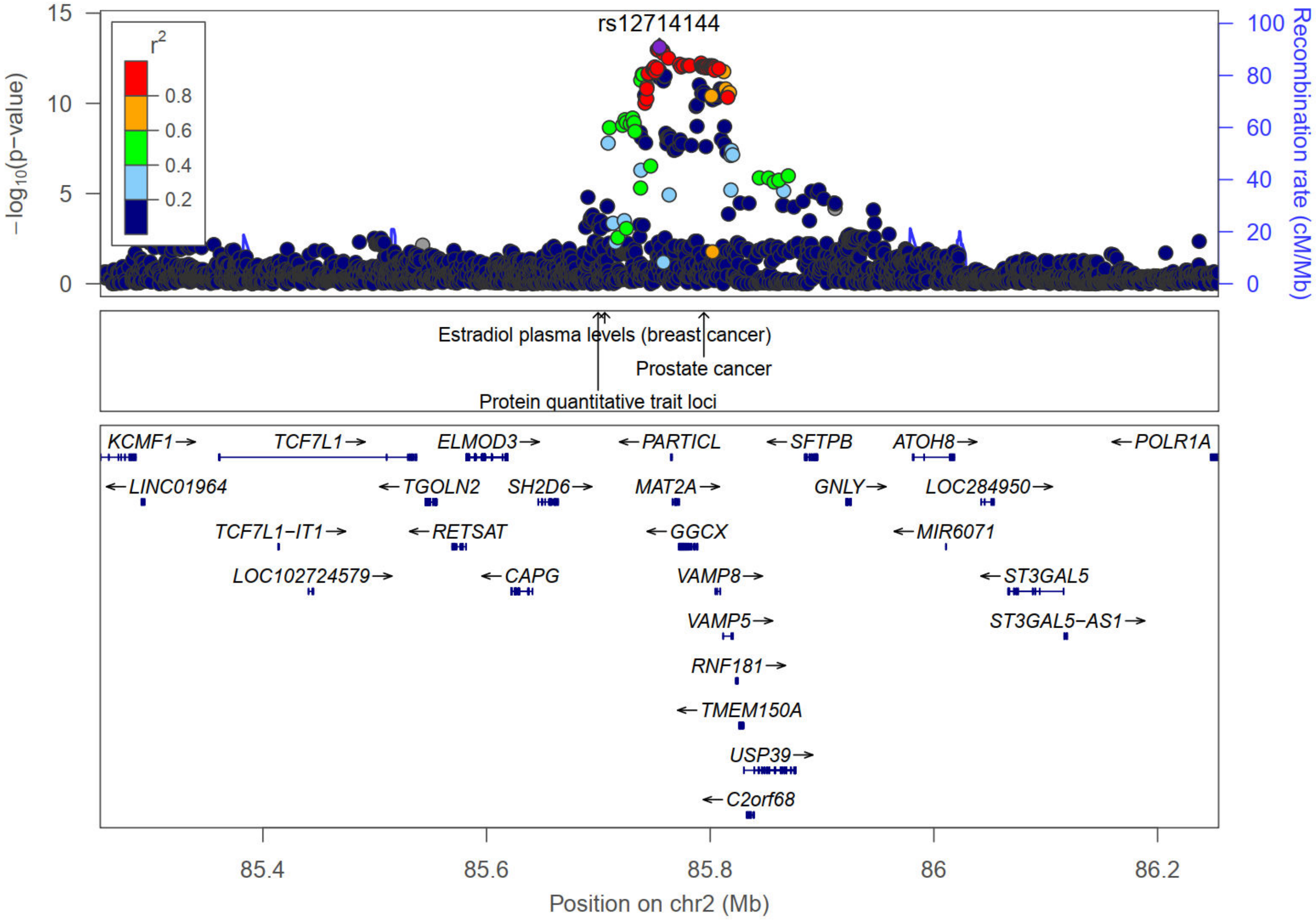

rs12714144
r²
0.8
0.6
0.4
0.2
−log10(p−value)
Recombination rate (cM/Mb)
Estradiol plasma levels (breast cancer)
Prostate cancer
Protein quantitative trait loci
KCMF1
TCF7L1
ELMOD3
PARTICL
SFTPB
ATOH8
POLR1A
LINC01964
TGOLN2
SH2D6
MAT2A
GNLY
LOC284950
TCF7L1−IT1
RETSAT
GGCX
MIR6071
LOC102724579
CAPG
VAMP8
ST3GAL5
VAMP5
ST3GAL5−AS1
RNF181
TMEM150A
USP39
C2orf68
Position on chr2 (Mb)

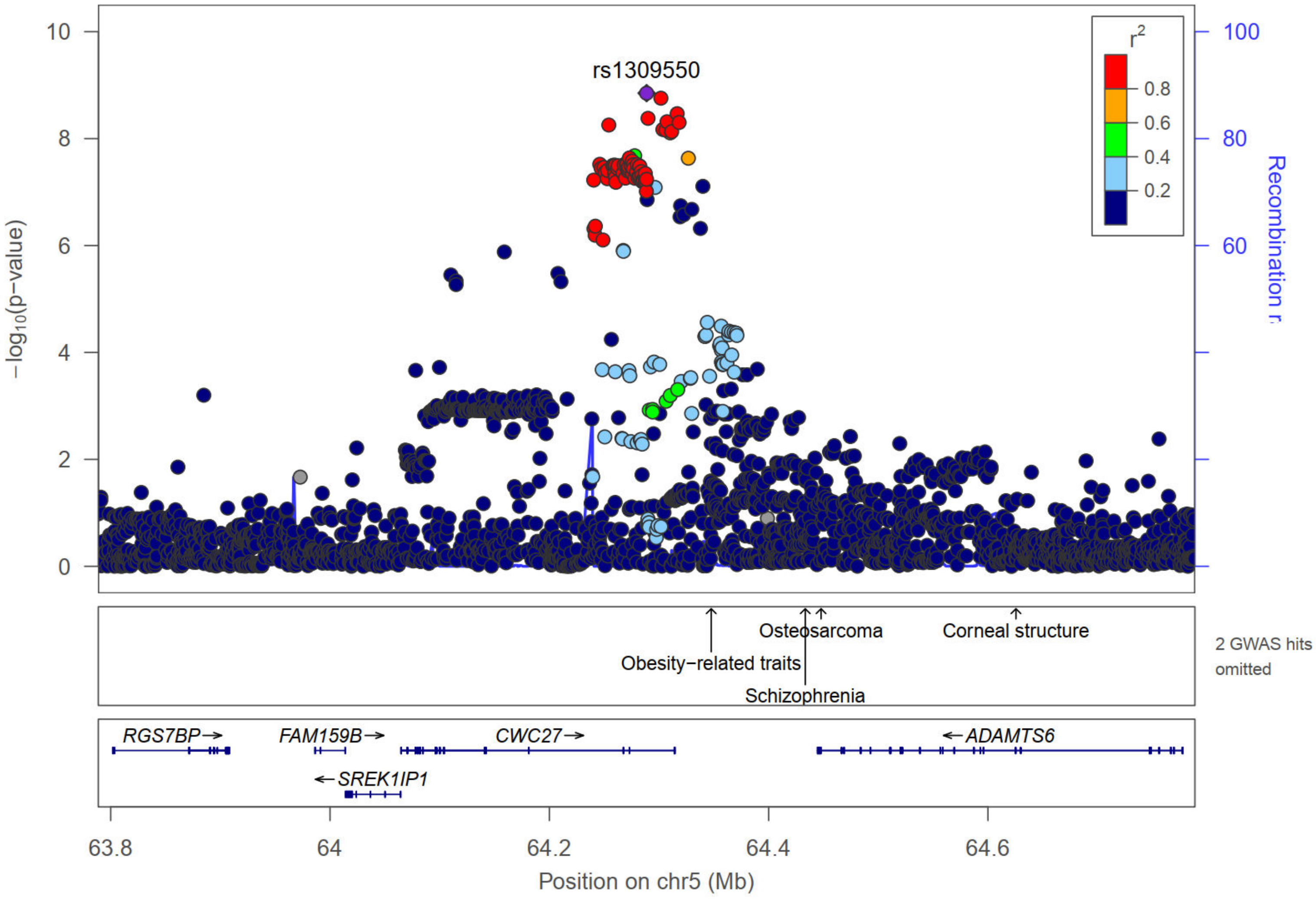
rs1309550
−log10(p−value)
Recombination r
r2
0.8
0.6
0.4
0.2
Osteosarcoma
Corneal structure
Obesity−related traits
Schizophrenia
2 GWAS hits omitted
RGS7BP→
FAM159B→
CWC27→
←ADAMTS6
←SREK1IP1
Position on chr5 (Mb)

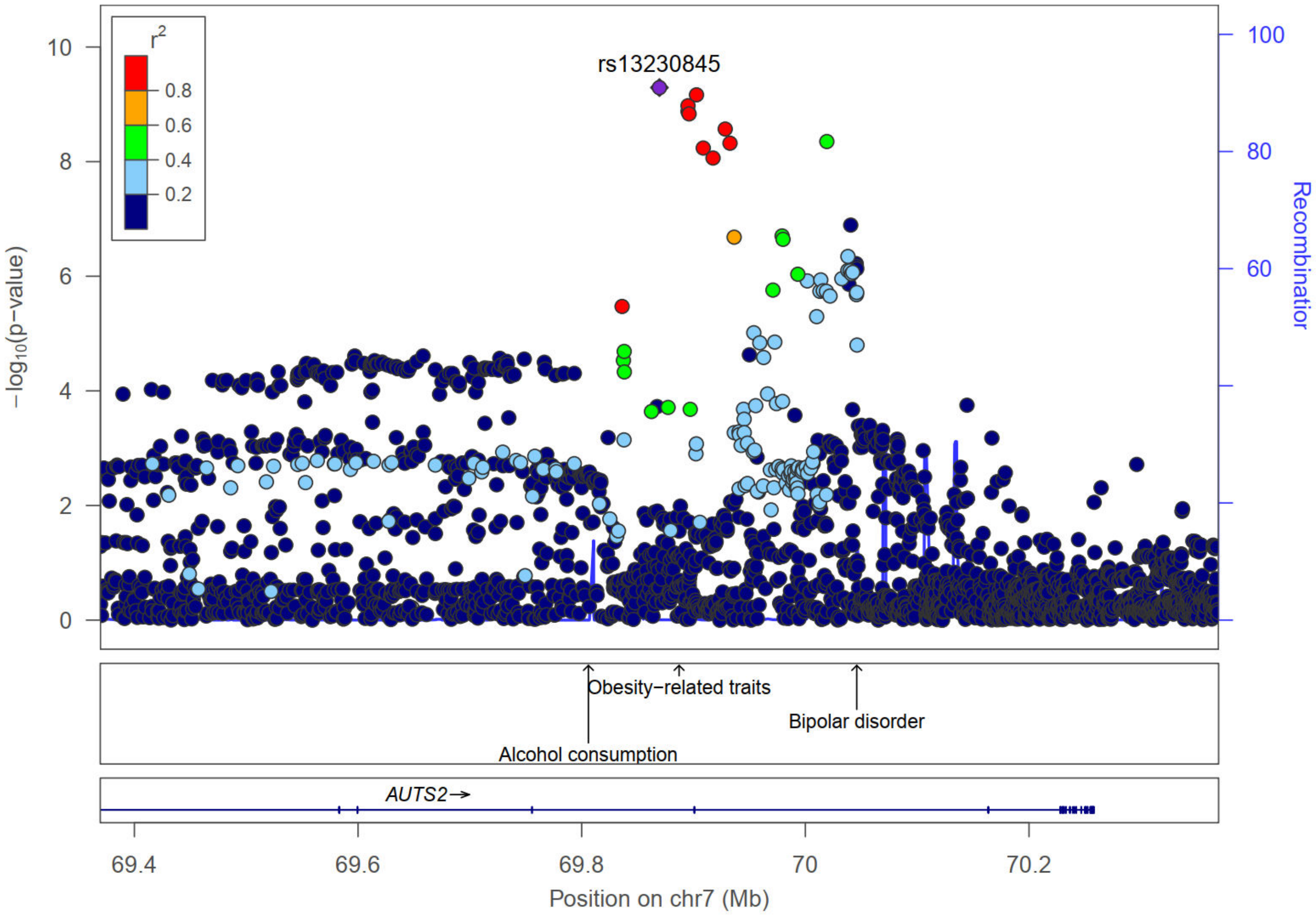

r2
0.8
0.6
0.4
0.2
rs13230845
−log10(p−value)
Recombination
10
8
6
4
2
0
100
80
60
Obesity−related traits
Bipolar disorder
Alcohol consumption
AUTS2→
69.4
69.6
69.8
70
70.2
Position on chr7 (Mb)

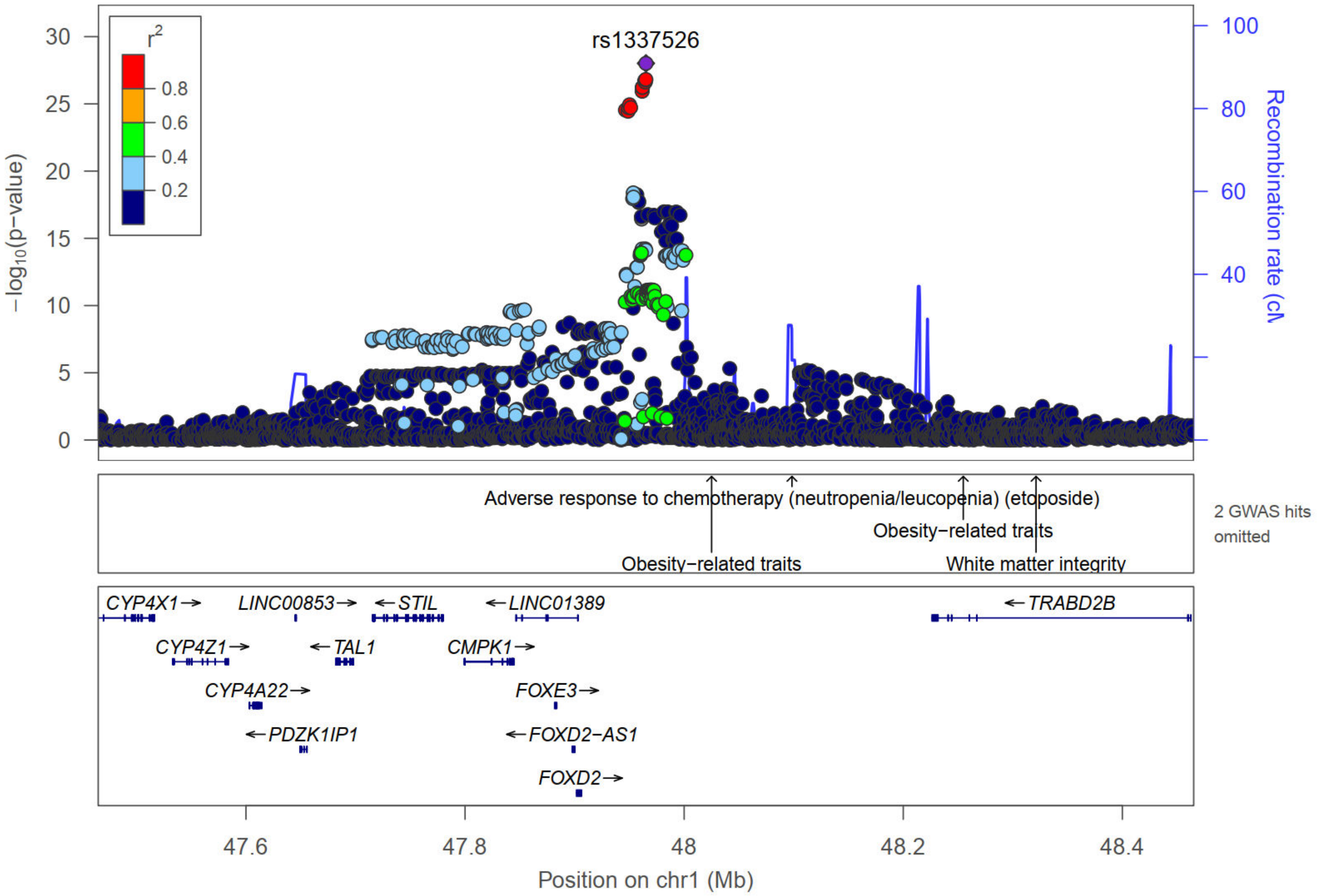

rs1337526
r2
0.8
0.6
0.4
0.2
−log10(p−value)
Recombination rate (cM
Adverse response to chemotherapy (neutropenia/leucopenia) (etoposide)
Obesity−related traits
Obesity−related traits
White matter integrity
2 GWAS hits omitted
CYP4X1→
LINC00853→
←STIL
←LINC01389
←TRABD2B
CYP4Z1→
←TAL1
CMPK1→
CYP4A22→
FOXE3→
←PDZK1IP1
←FOXD2−AS1
FOXD2→
Position on chr1 (Mb)

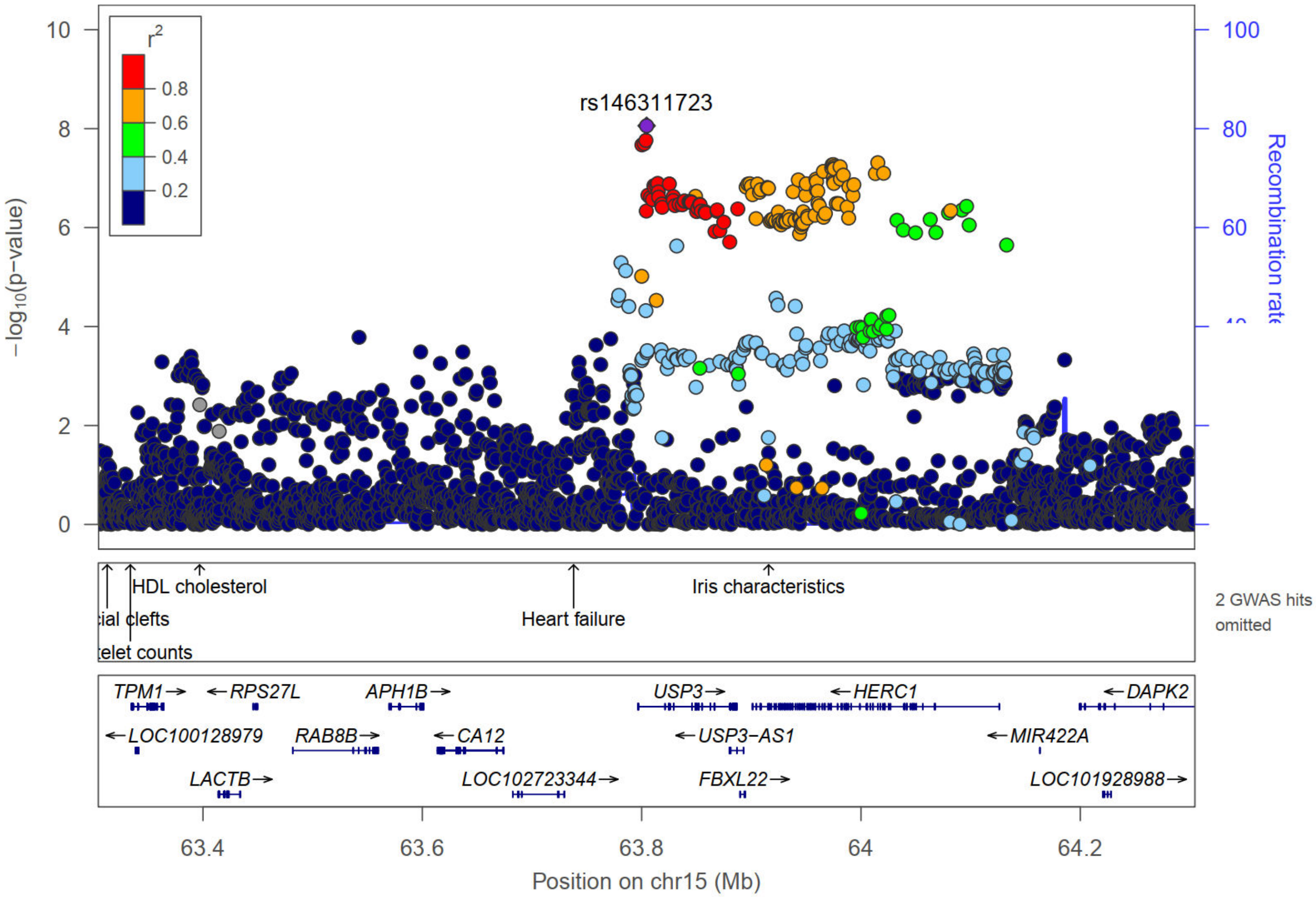

rs146311723
r2
0.8
0.6
0.4
0.2
−log10(p−value)
Recombination rate
HDL cholesterol
Heart failure
Iris characteristics
2 GWAS hits omitted
TPM1
RPS27L
APH1B
USP3
HERC1
DAPK2
LOC100128979
RAB8B
CA12
USP3−AS1
MIR422A
LACTB
LOC102723344
FBXL22
LOC101928988
63.4
63.6
63.8
64
64.2
Position on chr15 (Mb)

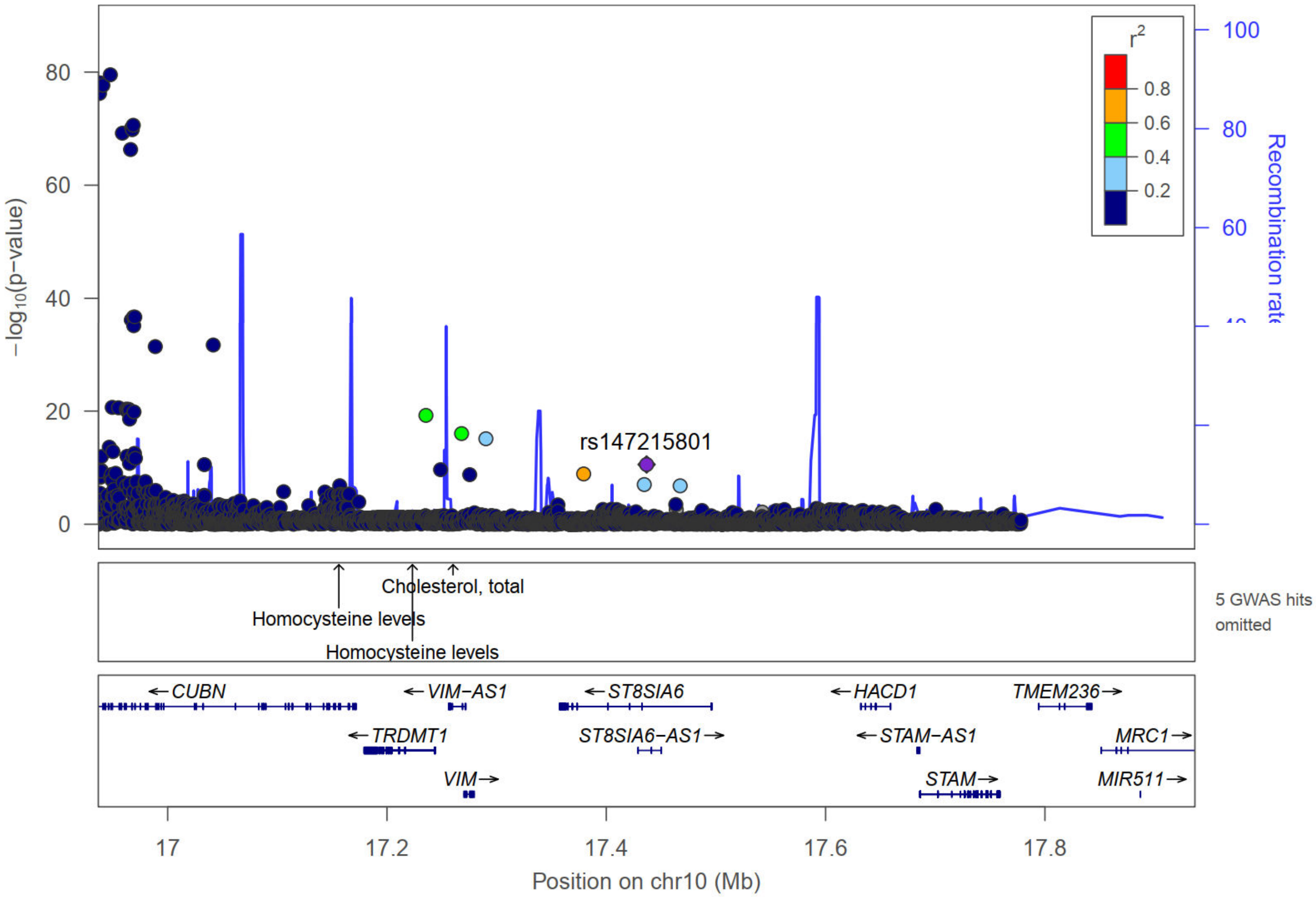

80
60
40
20
0
−log10(p−value)
100
80
60
Recombination rate
r2
0.8
0.6
0.4
0.2
rs147215801
Cholesterol, total
Homocysteine levels
Homocysteine levels
5 GWAS hits omitted
CUBN
VIM−AS1
ST8SIA6
HACD1
TMEM236
TRDMT1
ST8SIA6−AS1
STAM−AS1
MRC1
VIM
STAM
MIR511
17
17.2
17.4
17.6
17.8
Position on chr10 (Mb)

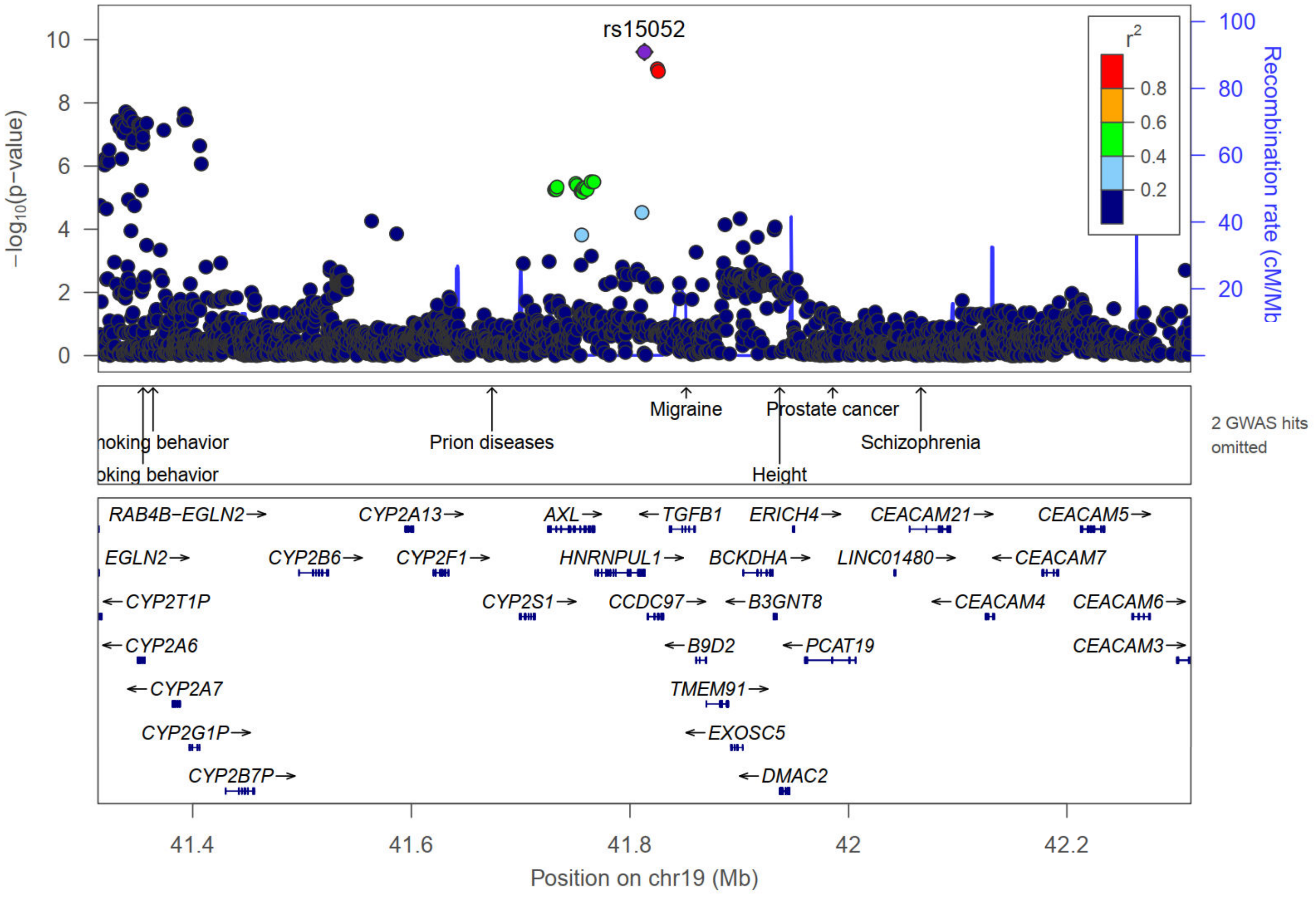

rs15052
−log10(p−value)
Recombination rate (cM/Mb)
r2
0.8
0.6
0.4
0.2
Migraine
Prostate cancer
Schizophrenia
Prion diseases
Height
2 GWAS hits omitted
RAB4B−EGLN2→
CYP2A13→
AXL→
←TGFB1
ERICH4→
CEACAM21→
CEACAM5→
EGLN2→
CYP2B6→
CYP2F1→
HNRNPUL1→
BCKDHA→
LINC01480→
←CEACAM7
←CYP2T1P
CYP2S1→
CCDC97→
←B3GNT8
←CEACAM4
CEACAM6→
←CYP2A6
←B9D2
←PCAT19
CEACAM3→
←CYP2A7
TMEM91→
CYP2G1P→
←EXOSC5
CYP2B7P→
←DMAC2
Position on chr19 (Mb)

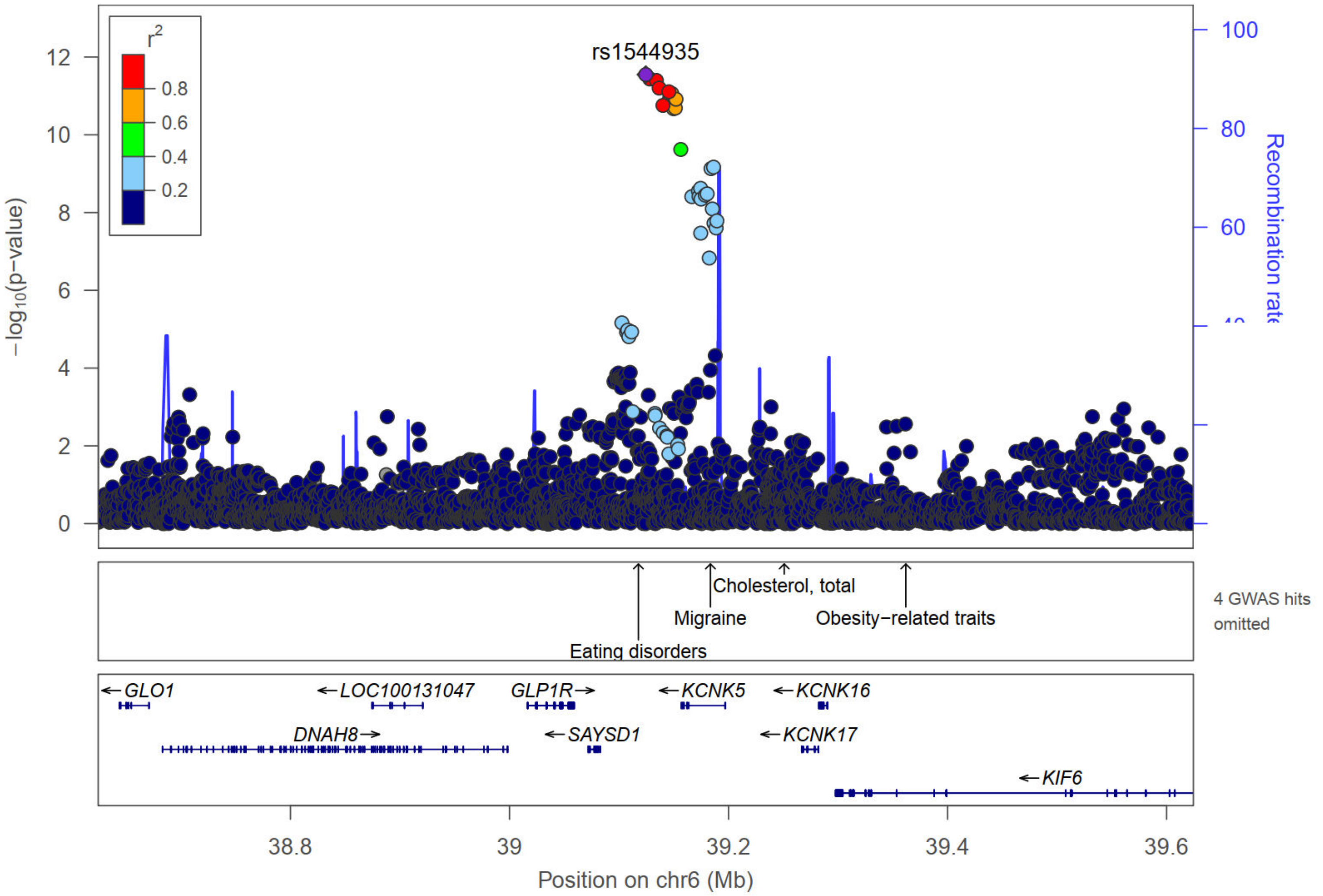

rs1544935
r2
0.8
0.6
0.4
0.2
−log10(p−value)
Recombination rate
Cholesterol, total
Migraine
Obesity−related traits
Eating disorders
4 GWAS hits omitted
GLO1
LOC100131047
GLP1R
KCNK5
KCNK16
DNAH8
SAYSD1
KCNK17
KIF6
38.8
39
39.2
39.4
39.6
Position on chr6 (Mb)

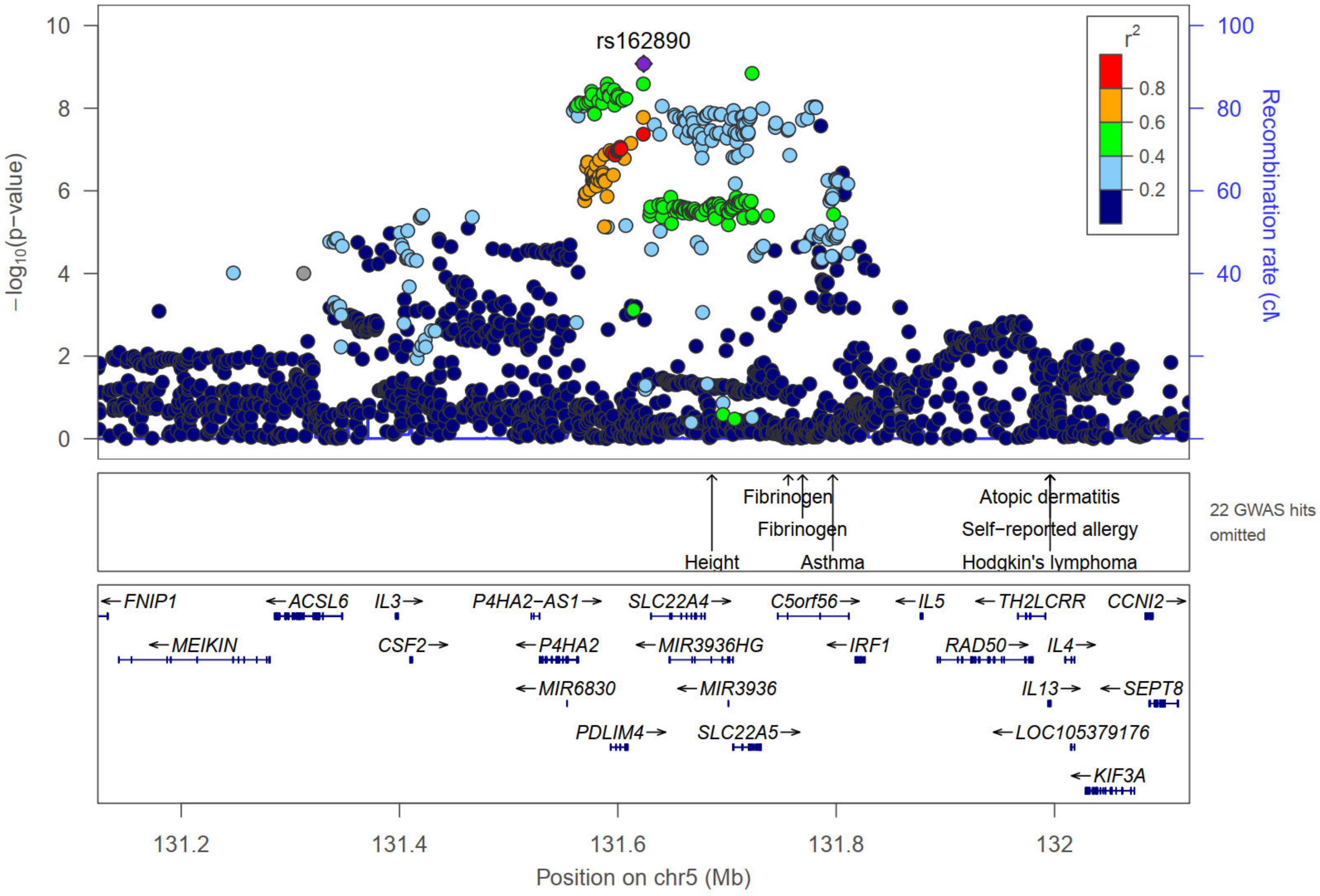
rs162890
−log10(p−value)
Recombination rate (cM
r2
0.8
0.6
0.4
0.2
Height
Fibrinogen
Fibrinogen
Asthma
Atopic dermatitis
Self−reported allergy
Hodgkin's lymphoma
22 GWAS hits omitted
FNIP1
ACSL6
IL3
P4HA2−AS1
SLC22A4
C5orf56
IL5
TH2LCRR
CCNI2
MEIKIN
CSF2
P4HA2
MIR3936HG
IRF1
RAD50
IL4
MIR6830
MIR3936
IL13
SEPT8
PDLIM4
SLC22A5
LOC105379176
KIF3A
Position on chr5 (Mb)

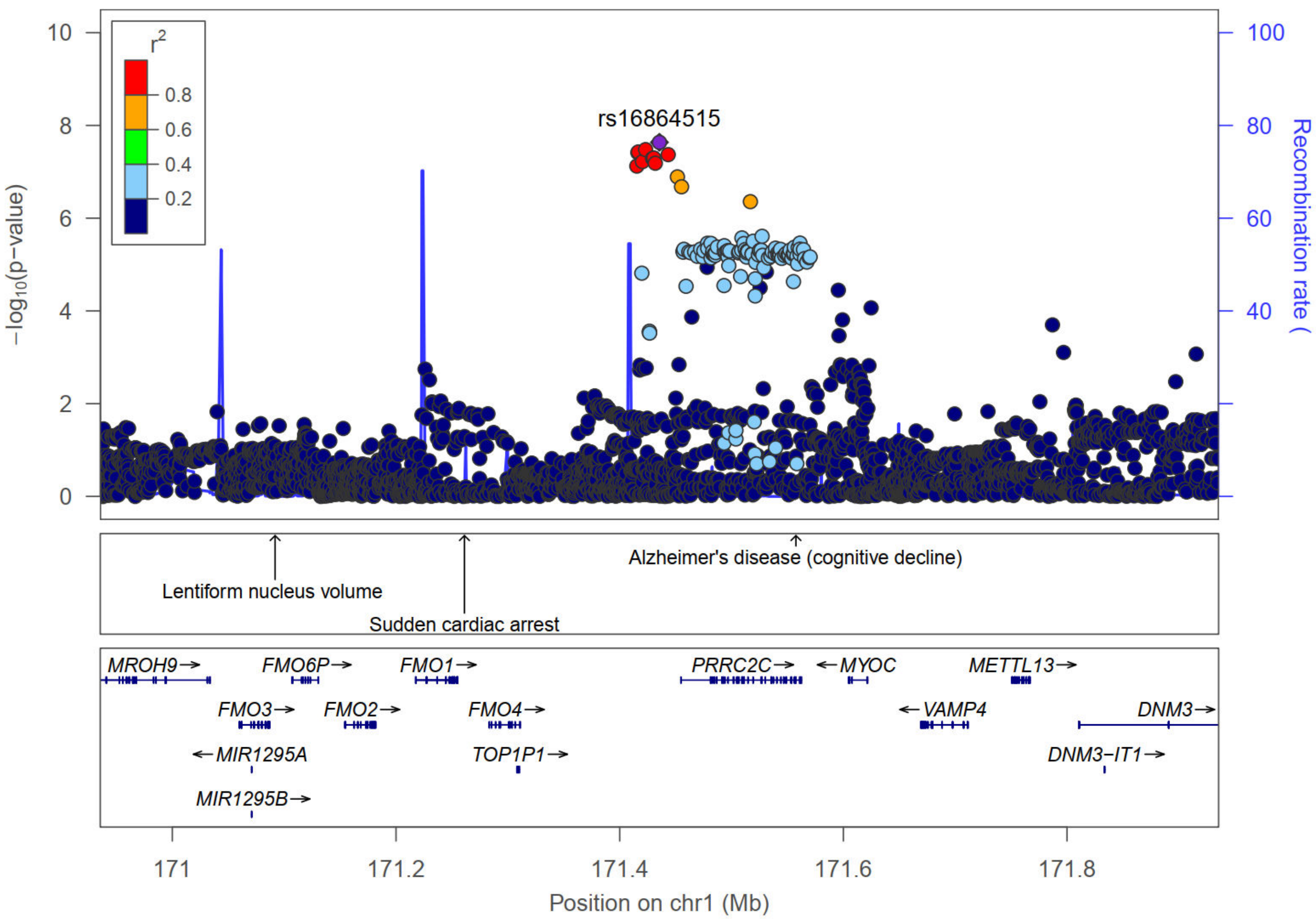

rs16864515
r²
0.8
0.6
0.4
0.2
10
8
6
4
2
0
-log10(p-value)
100
80
60
40
Recombination rate (
Lentiform nucleus volume
Sudden cardiac arrest
Alzheimer's disease (cognitive decline)
MROH9→
FMO6P→
FMO1→
PRRC2C→
←MYOC
METTL13→
FMO3→
FMO2→
FMO4→
←VAMP4
DNM3→
←MIR1295A
TOP1P1→
DNM3−IT1→
MIR1295B→
171
171.2
171.4
171.6
171.8
Position on chr1 (Mb)

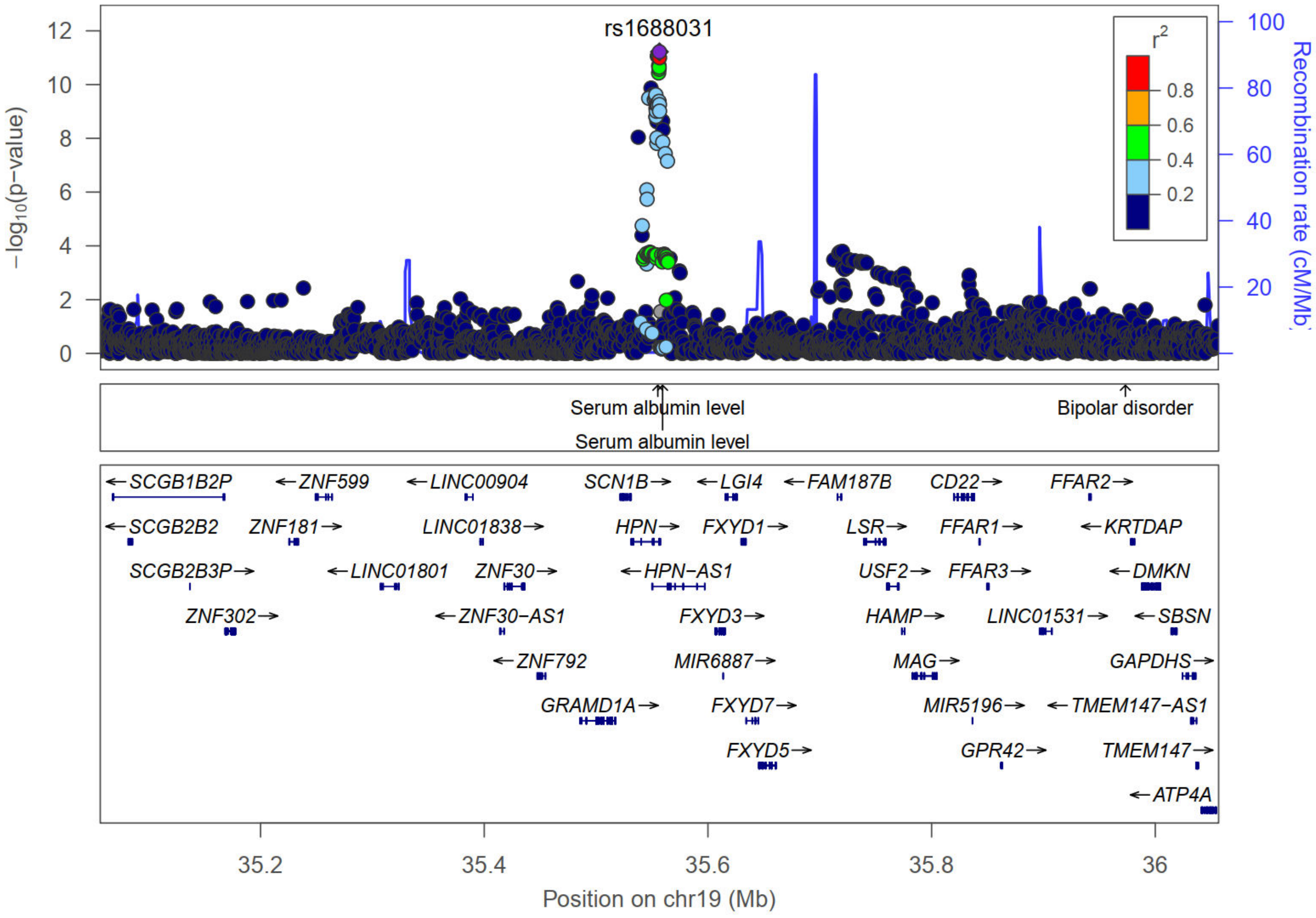
rs1688031
−log10(p−value)
Recombination rate (cM/Mb)
r2
0.8
0.6
0.4
0.2
Serum albumin level
Serum albumin level
Bipolar disorder
SCGB1B2P
ZNF599
LINC00904
SCN1B
LGI4
FAM187B
CD22
FFAR2
SCGB2B2
ZNF181
LINC01838
HPN
FXYD1
LSR
FFAR1
KRTDAP
SCGB2B3P
LINC01801
ZNF30
HPN−AS1
USF2
FFAR3
DMKN
ZNF302
ZNF30−AS1
FXYD3
HAMP
LINC01531
SBSN
ZNF792
MIR6887
MAG
GAPDHS
GRAMD1A
FXYD7
MIR5196
TMEM147−AS1
FXYD5
GPR42
TMEM147
ATP4A
Position on chr19 (Mb)

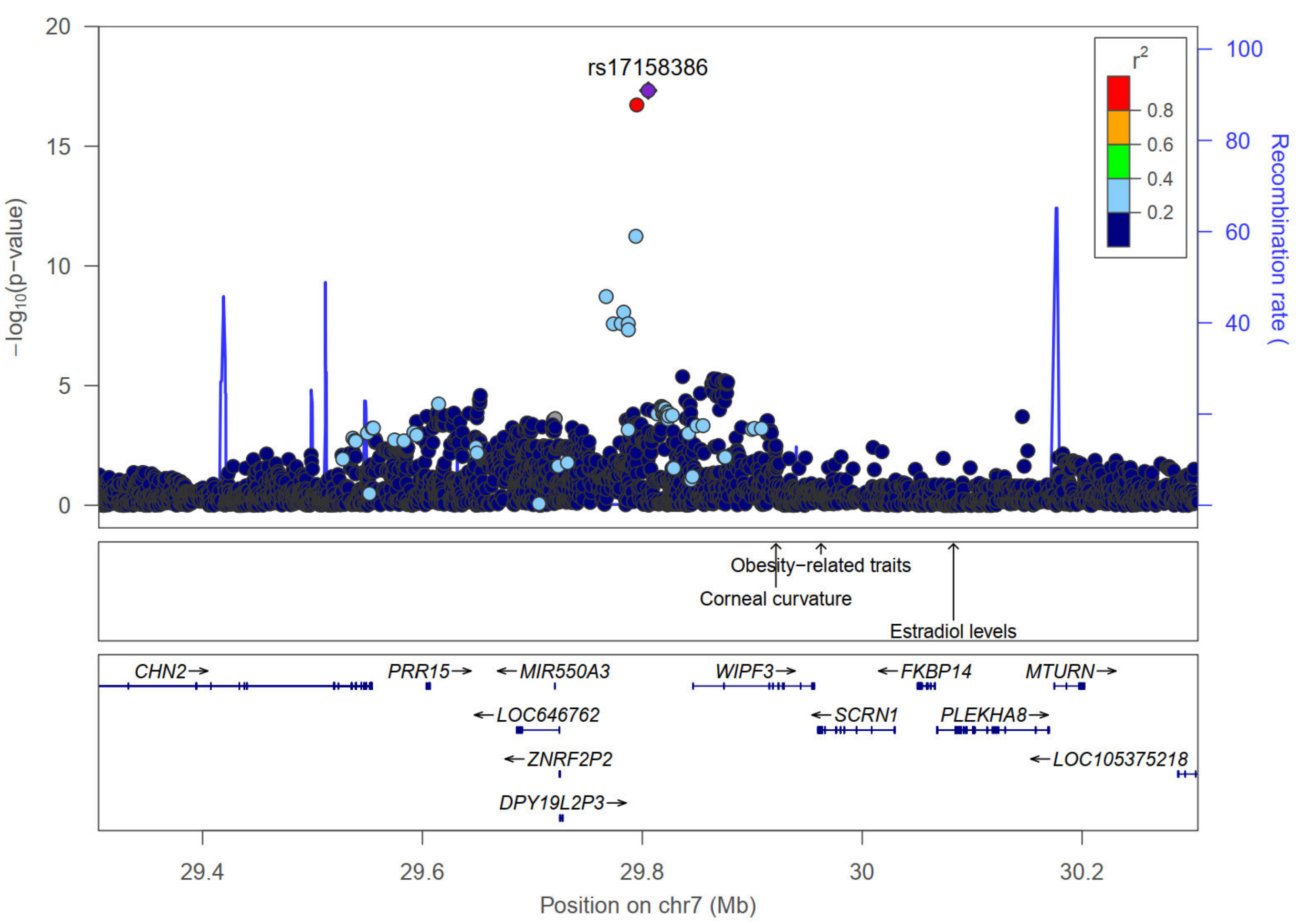

rs17158386
−log10(p−value)
Recombination rate (
r2
0.8
0.6
0.4
0.2
Obesity−related traits
Corneal curvature
Estradiol levels
CHN2→
PRR15→
←MIR550A3
WIPF3→
←FKBP14
MTURN→
←LOC646762
←SCRN1
PLEKHA8→
←ZNRF2P2
←LOC105375218
DPY19L2P3→
29.4
29.6
29.8
30
30.2
Position on chr7 (Mb)

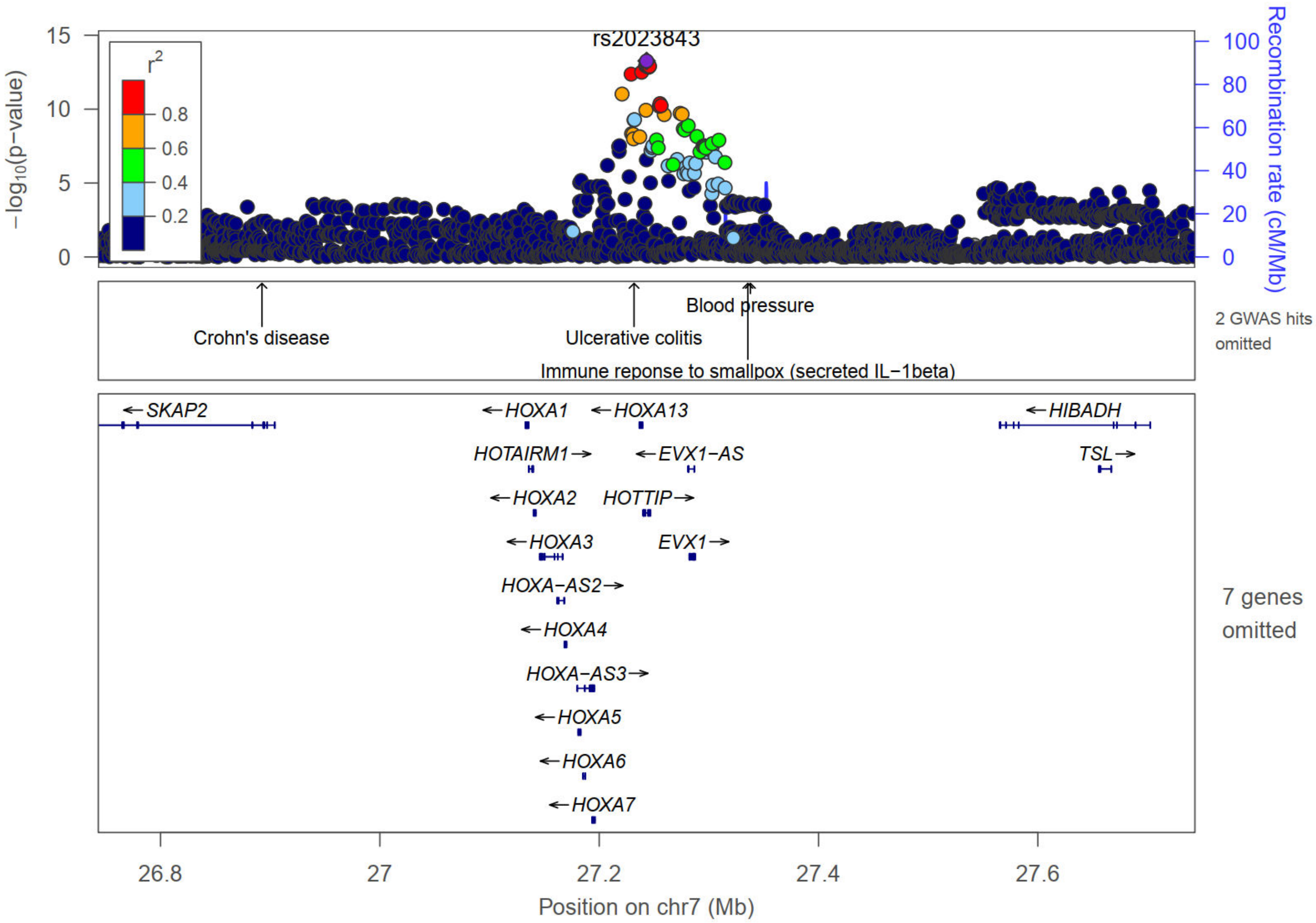
rs2023843
−log10(p−value)
Recombination rate (cM/Mb)
r2
0.8
0.6
0.4
0.2
Crohn's disease
Ulcerative colitis
Blood pressure
Immune reponse to smallpox (secreted IL−1beta)
2 GWAS hits omitted
SKAP2
HOXA1
HOXA13
HIBADH
HOTAIRM1
EVX1−AS
TSL
HOXA2
HOTTIP
HOXA3
EVX1
HOXA−AS2
HOXA4
HOXA−AS3
HOXA5
HOXA6
HOXA7
7 genes omitted
26.8
27
27.2
27.4
27.6
Position on chr7 (Mb)

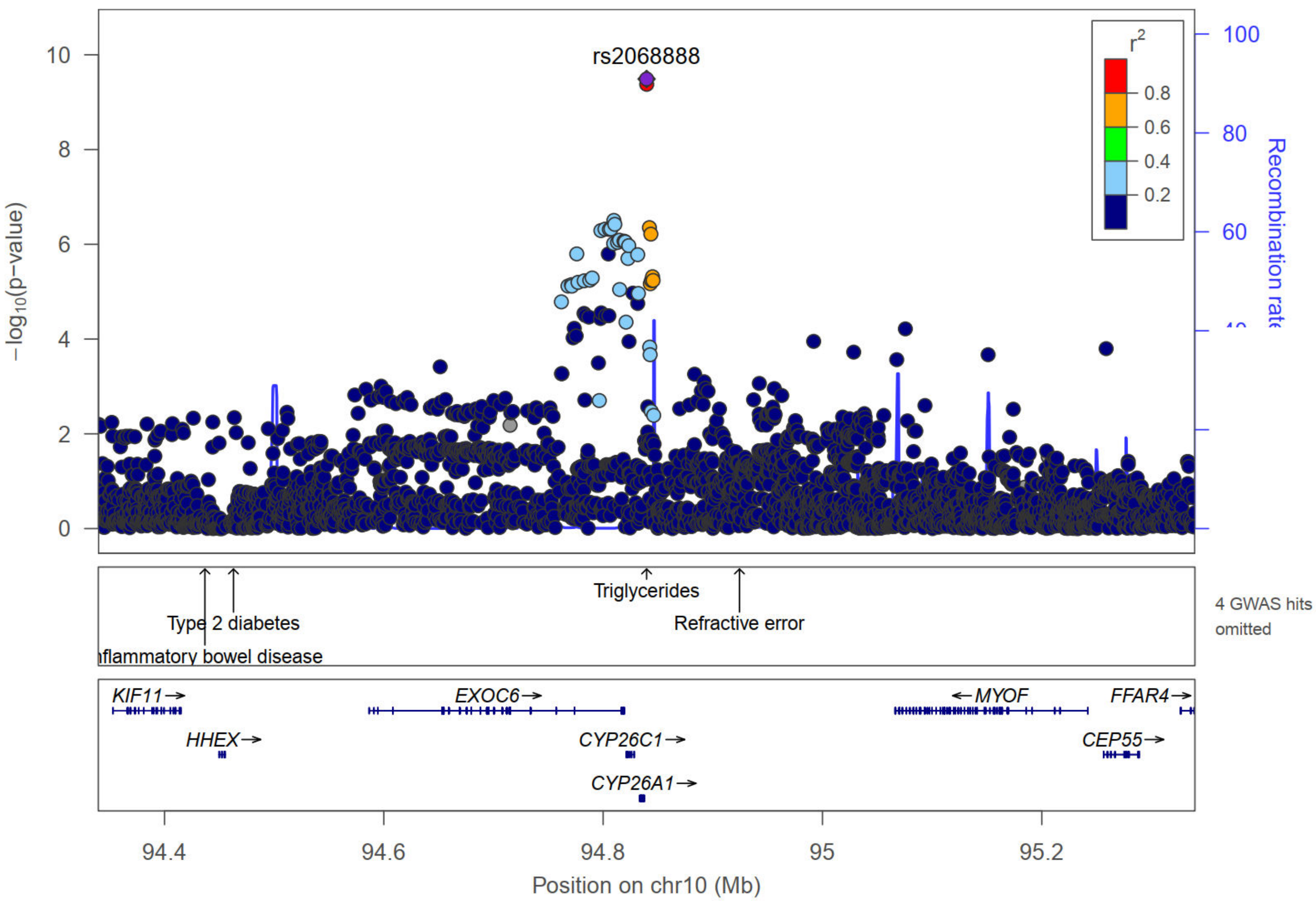

rs2068888
−log10(p−value)
Recombination rate
r2
0.8
0.6
0.4
0.2
Triglycerides
Type 2 diabetes
Refractive error
nflammatory bowel disease
4 GWAS hits omitted
KIF11→
EXOC6→
←MYOF
FFAR4→
HHEX→
CYP26C1→
CEP55→
CYP26A1→
94.4
94.6
94.8
95
95.2
Position on chr10 (Mb)

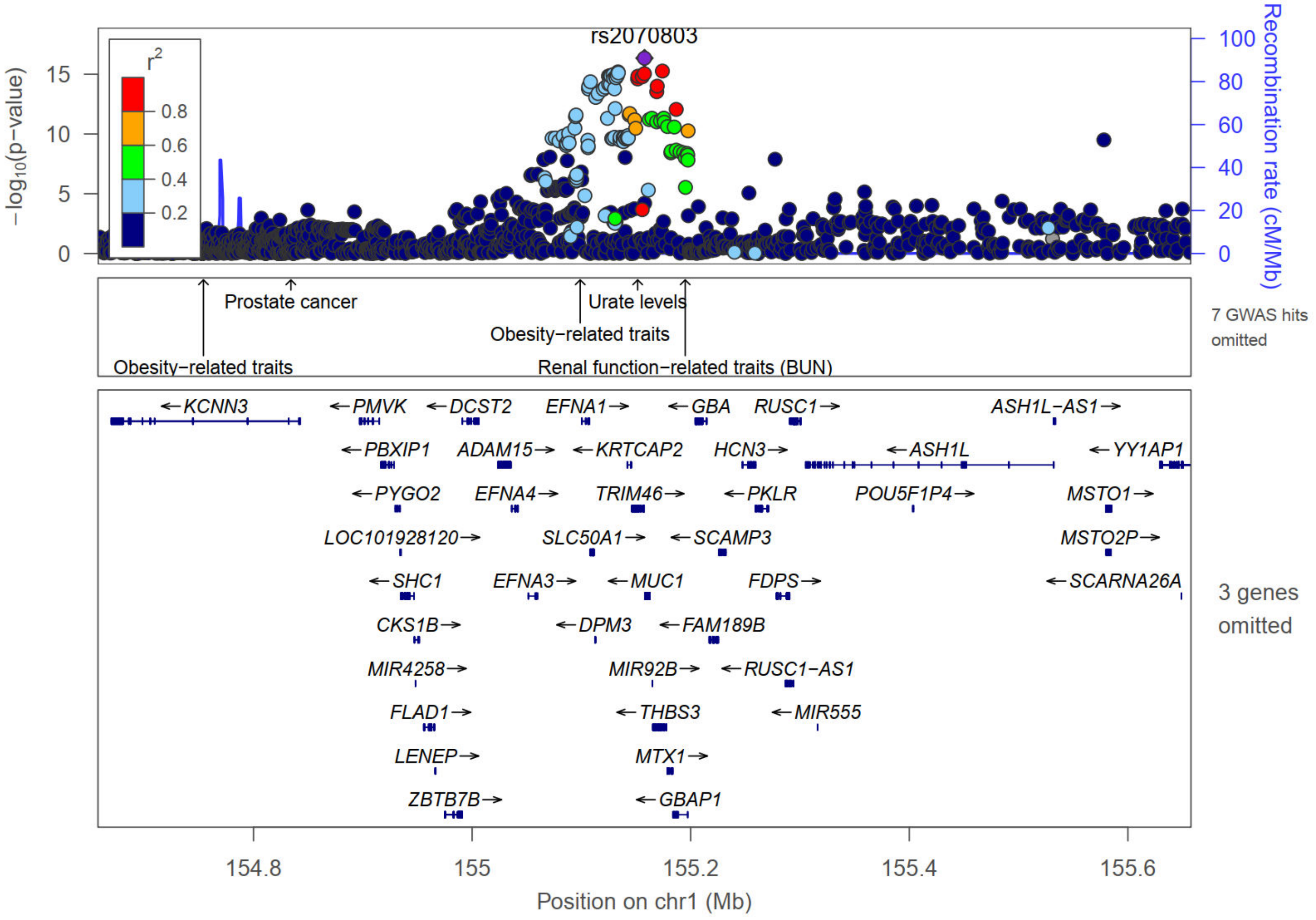

rs2070803
−log10(p−value)
Recombination rate (cM/Mb)
r2
0.8
0.6
0.4
0.2
Prostate cancer
Urate levels
Obesity−related traits
Obesity−related traits
Renal function−related traits (BUN)
7 GWAS hits omitted
KCNN3
PMVK
DCST2
EFNA1
GBA
RUSC1
ASH1L−AS1
PBXIP1
ADAM15
KRTCAP2
HCN3
ASH1L
YY1AP1
PYGO2
EFNA4
TRIM46
PKLR
POU5F1P4
MSTO1
LOC101928120
SLC50A1
SCAMP3
MSTO2P
SHC1
EFNA3
MUC1
FDPS
SCARNA26A
CKS1B
DPM3
FAM189B
MIR4258
MIR92B
RUSC1−AS1
FLAD1
THBS3
MIR555
LENEP
MTX1
ZBTB7B
GBAP1
3 genes omitted
154.8
155
155.2
155.4
155.6
Position on chr1 (Mb)

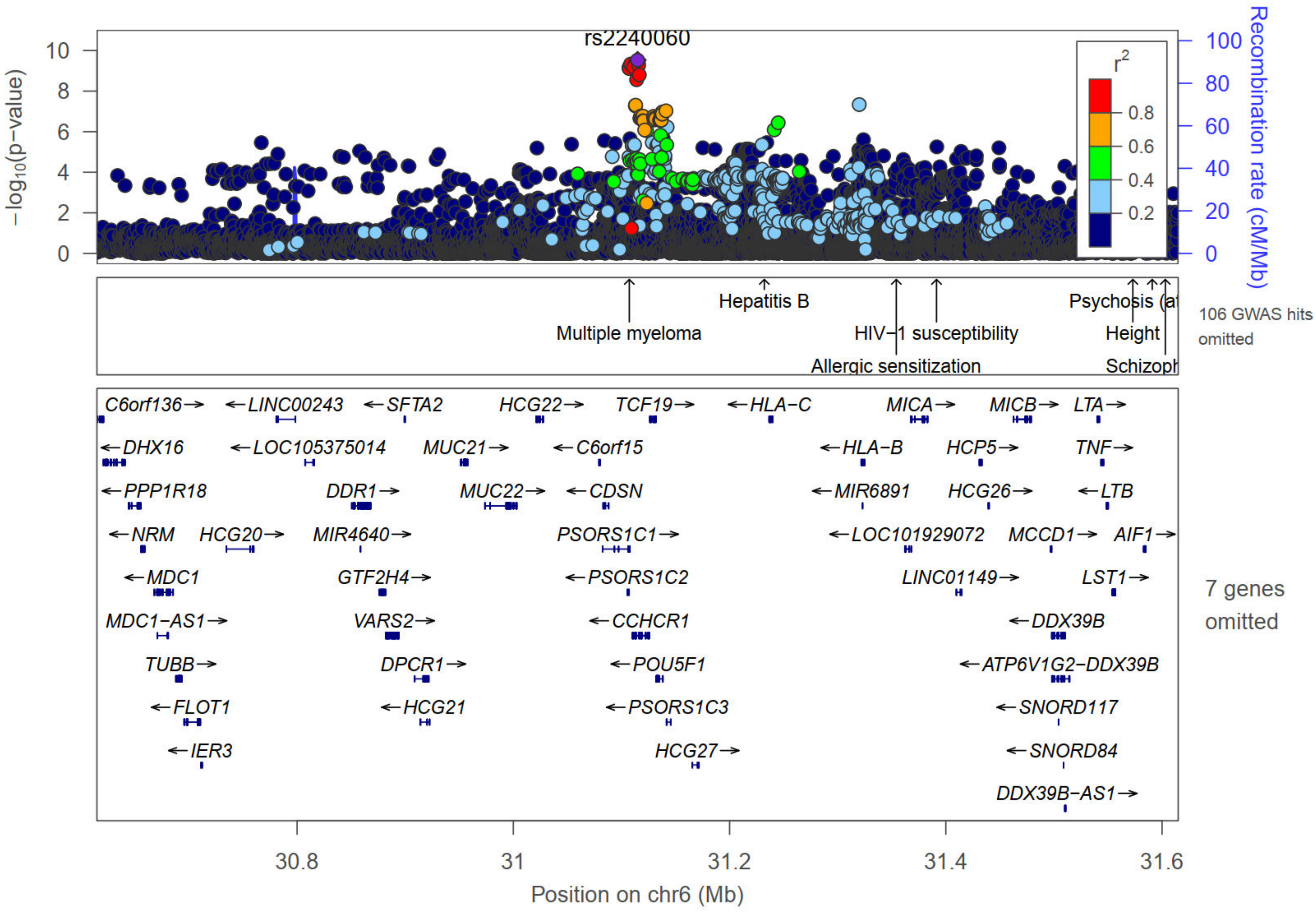

rs2240060
−log10(p−value)
Recombination rate (cM/Mb)
r2
0.8
0.6
0.4
0.2
Multiple myeloma
Hepatitis B
HIV−1 susceptibility
Allergic sensitization
Psychosis (at
Height
Schizoph
106 GWAS hits omitted
7 genes omitted
C6orf136 LINC00243 SFTA2 HCG22 TCF19 HLA−C MICA MICB LTA
DHX16 LOC105375014 MUC21 C6orf15 HLA−B HCP5 TNF
PPP1R18 DDR1 MUC22 CDSN MIR6891 HCG26 LTB
NRM HCG20 MIR4640 PSORS1C1 LOC101929072 MCCD1 AIF1
MDC1 GTF2H4 PSORS1C2 LINC01149 LST1
MDC1−AS1 VARS2 CCHCR1 DDX39B
TUBB DPCR1 POU5F1 ATP6V1G2−DDX39B
FLOT1 HCG21 PSORS1C3 SNORD117
IER3 HCG27 SNORD84
DDX39B−AS1
30.8
31
31.2
31.4
31.6
Position on chr6 (Mb)

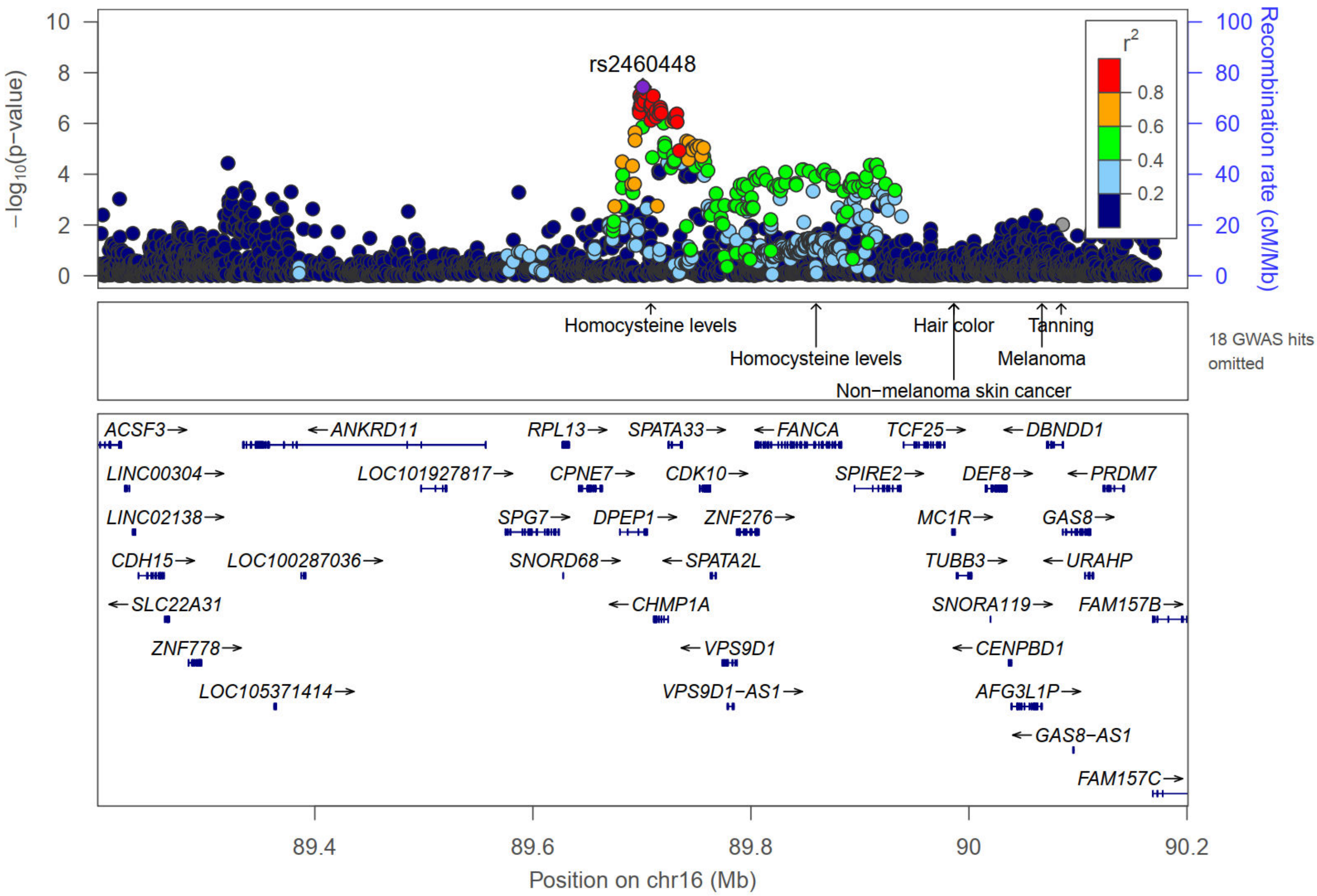

rs2460448
−log10(p−value)
Recombination rate (cM/Mb)
r2
0.8
0.6
0.4
0.2
Homocysteine levels
Homocysteine levels
Hair color
Non−melanoma skin cancer
Tanning
Melanoma
18 GWAS hits omitted
ACSF3
ANKRD11
RPL13
SPATA33
FANCA
TCF25
DBNDD1
LINC00304
LOC101927817
CPNE7
CDK10
SPIRE2
DEF8
PRDM7
LINC02138
SPG7
DPEP1
ZNF276
MC1R
GAS8
CDH15
LOC100287036
SNORD68
SPATA2L
TUBB3
URAHP
SLC22A31
CHMP1A
SNORA119
FAM157B
ZNF778
VPS9D1
CENPBD1
LOC105371414
VPS9D1−AS1
AFG3L1P
GAS8−AS1
FAM157C
Position on chr16 (Mb)

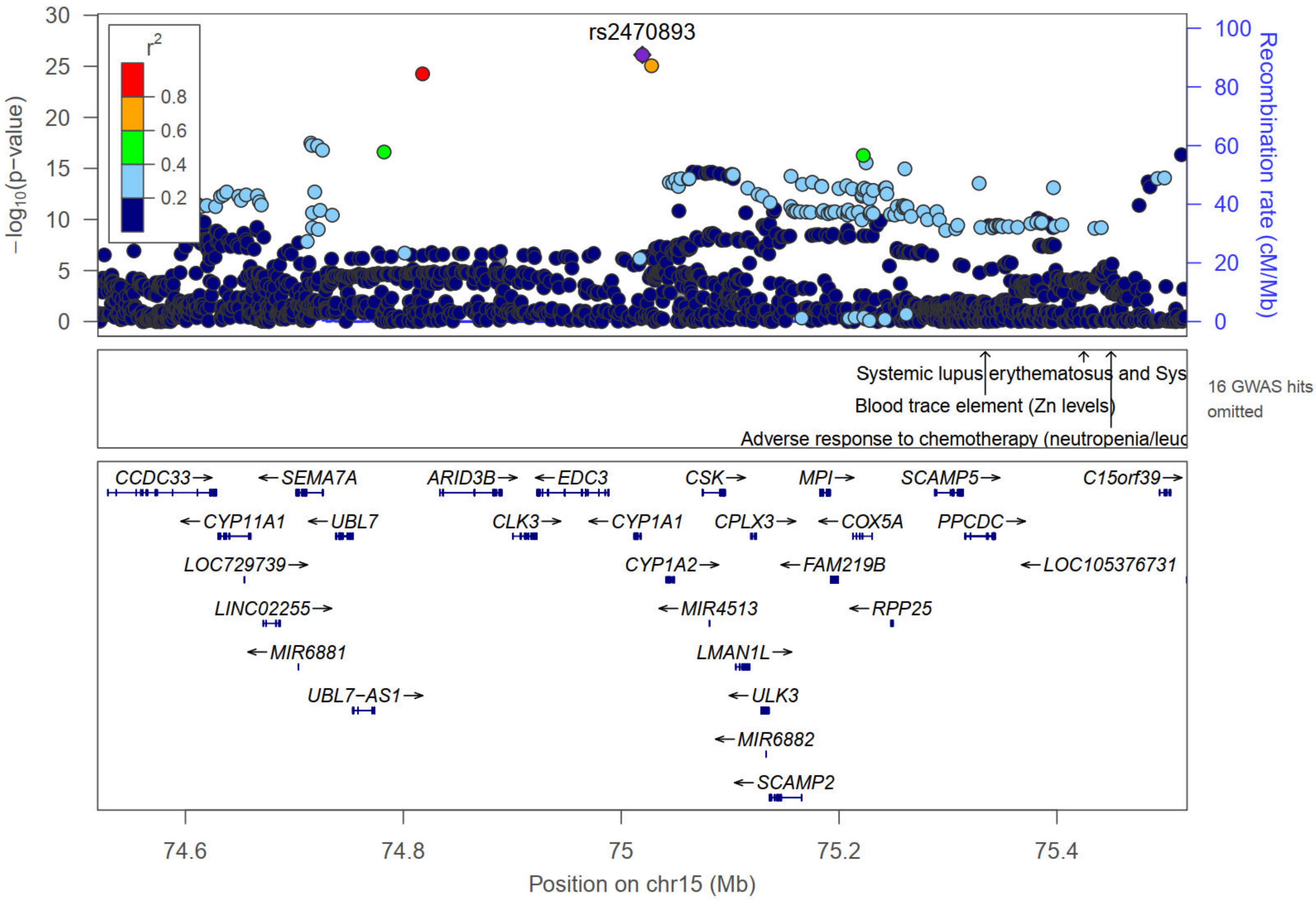

rs2470893
r2
0.8
0.6
0.4
0.2
−log10(p−value)
Recombination rate (cM/Mb)
Systemic lupus erythematosus and Sys
Blood trace element (Zn levels)
Adverse response to chemotherapy (neutropenia/leuc
16 GWAS hits omitted
CCDC33
SEMA7A
ARID3B
EDC3
CSK
MPI
SCAMP5
C15orf39
CYP11A1
UBL7
CLK3
CYP1A1
CPLX3
COX5A
PPCDC
LOC729739
CYP1A2
FAM219B
LOC105376731
LINC02255
MIR4513
RPP25
MIR6881
LMAN1L
UBL7−AS1
ULK3
MIR6882
SCAMP2
Position on chr15 (Mb)

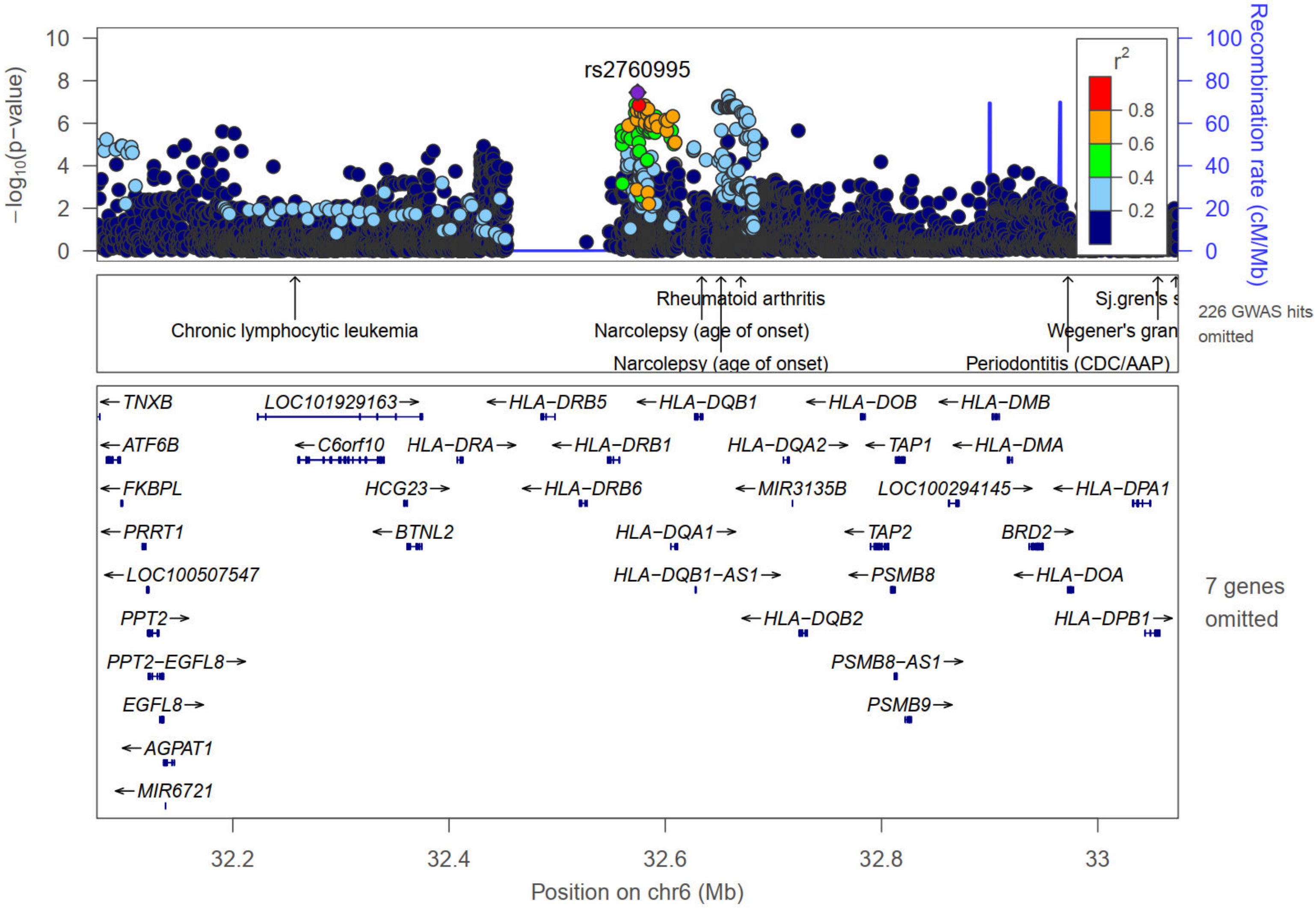
rs2760995
-log10(p-value)
Recombination rate (cM/Mb)
r2
0.8
0.6
0.4
0.2
Chronic lymphocytic leukemia
Rheumatoid arthritis
Narcolepsy (age of onset)
Narcolepsy (age of onset)
Sj.gren's s
Wegener's gran
Periodontitis (CDC/AAP)
226 GWAS hits omitted
TNXB
ATF6B
FKBPL
PRRT1
LOC100507547
PPT2
PPT2-EGFL8
EGFL8
AGPAT1
MIR6721
LOC101929163
C6orf10
HCG23
BTNL2
HLA-DRA
HLA-DRB5
HLA-DRB1
HLA-DRB6
HLA-DQB1
HLA-DQA1
HLA-DQB1-AS1
HLA-DQA2
MIR3135B
HLA-DQB2
HLA-DOB
TAP1
TAP2
PSMB8
PSMB8-AS1
PSMB9
LOC100294145
HLA-DMB
HLA-DMA
BRD2
HLA-DOA
HLA-DPA1
HLA-DPB1
7 genes omitted
Position on chr6 (Mb)

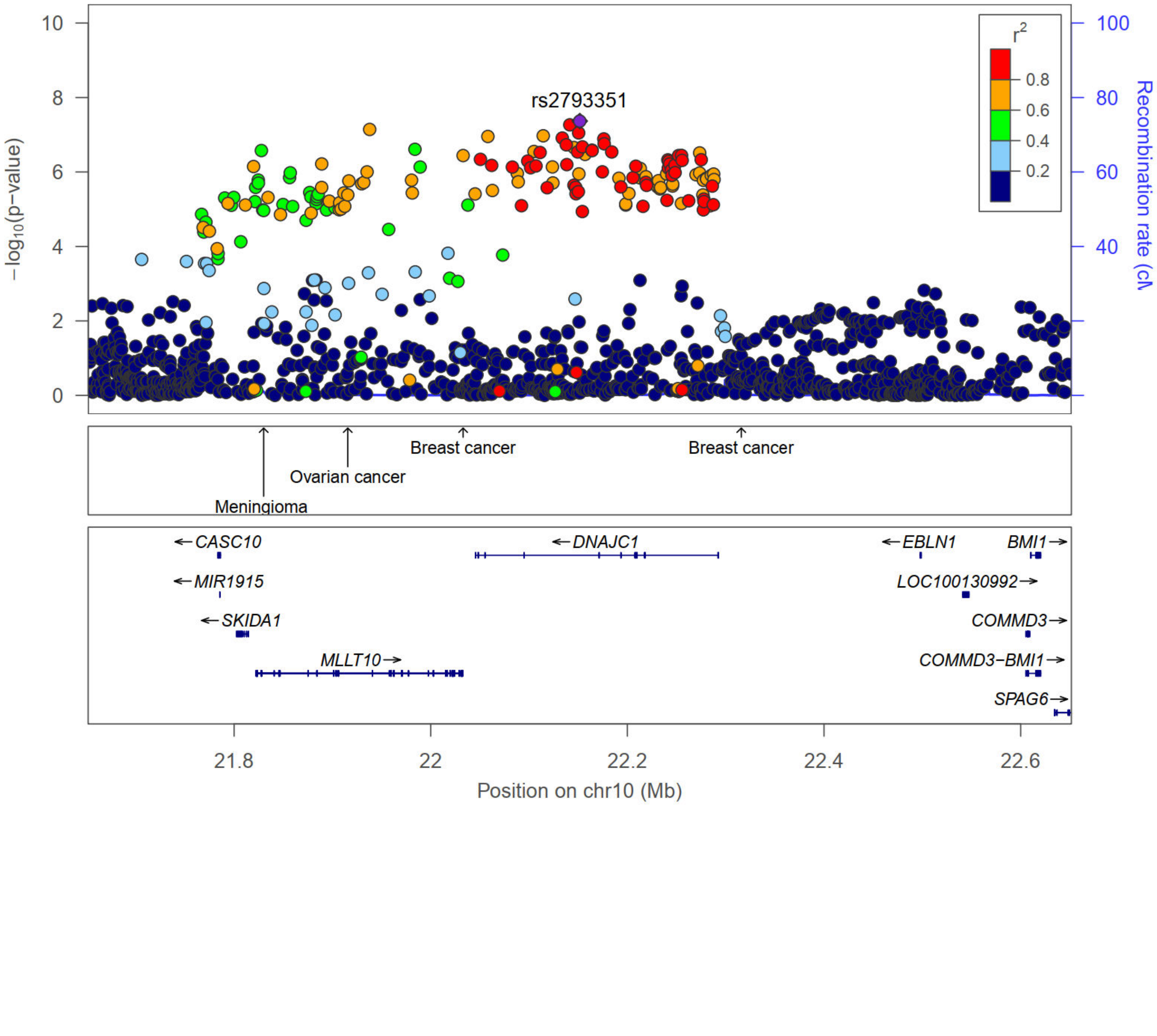
rs2793351
r2
0.8
0.6
0.4
0.2
−log10(p−value)
Recombination rate (cM
Meningioma
Ovarian cancer
Breast cancer
Breast cancer
CASC10
MIR1915
SKIDA1
MLLT10
DNAJC1
EBLN1
BMI1
LOC100130992
COMMD3
COMMD3−BMI1
SPAG6
21.8
22
22.2
22.4
22.6
Position on chr10 (Mb)

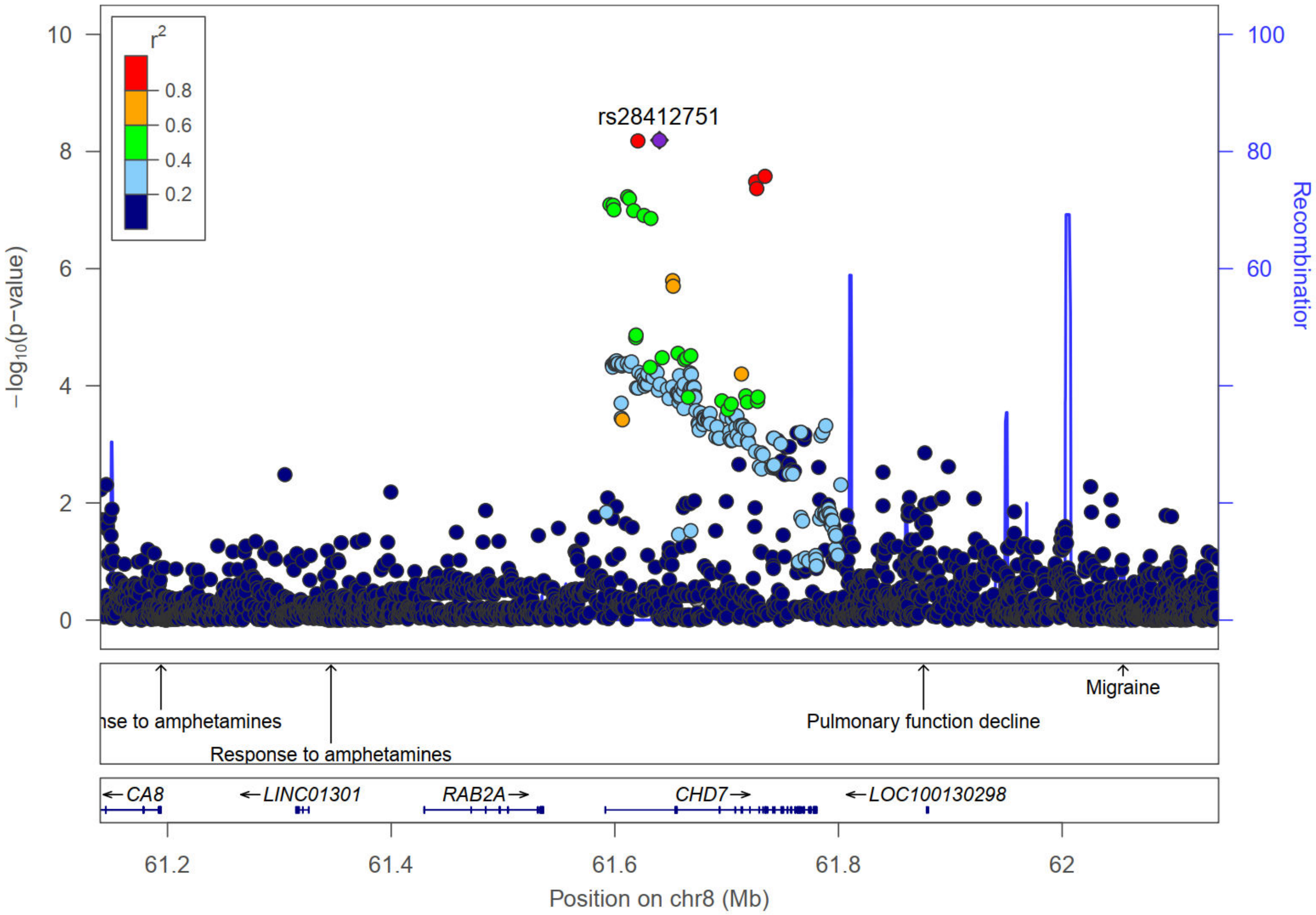

r2
0.8
0.6
0.4
0.2
rs28412751
10
8
6
4
2
0
−log10(p−value)
100
80
60
Recombination
nse to amphetamines
Response to amphetamines
Pulmonary function decline
Migraine
←CA8
←LINC01301
RAB2A→
CHD7→
←LOC100130298
61.2
61.4
61.6
61.8
62
Position on chr8 (Mb)

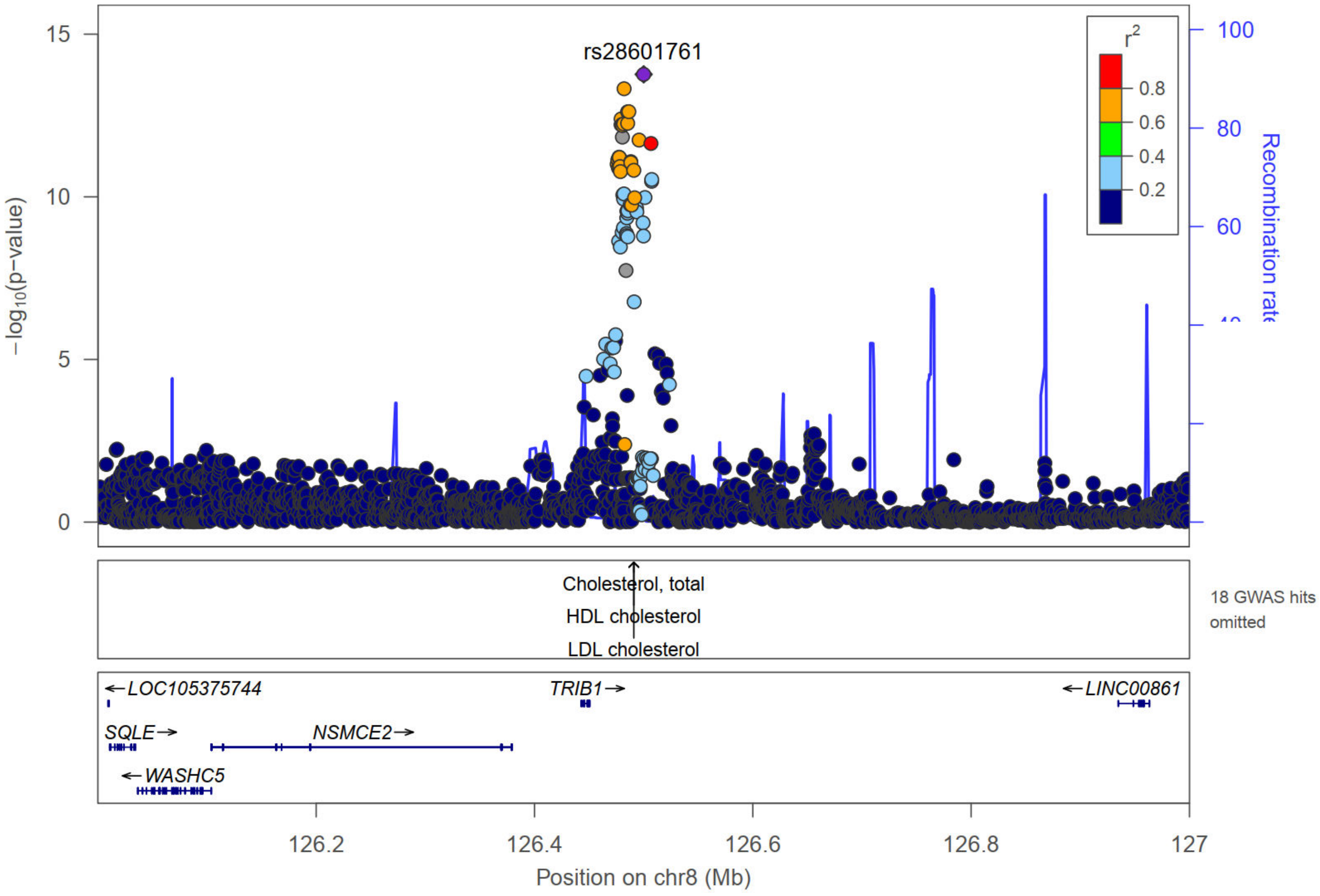

rs28601761
15
10
5
0
−log10(p−value)
100
80
60
40
Recombination rate
r2
0.8
0.6
0.4
0.2
Cholesterol, total
HDL cholesterol
LDL cholesterol
18 GWAS hits omitted
LOC105375744
TRIB1
LINC00861
SQLE
NSMCE2
WASHC5
126.2
126.4
126.6
126.8
127
Position on chr8 (Mb)

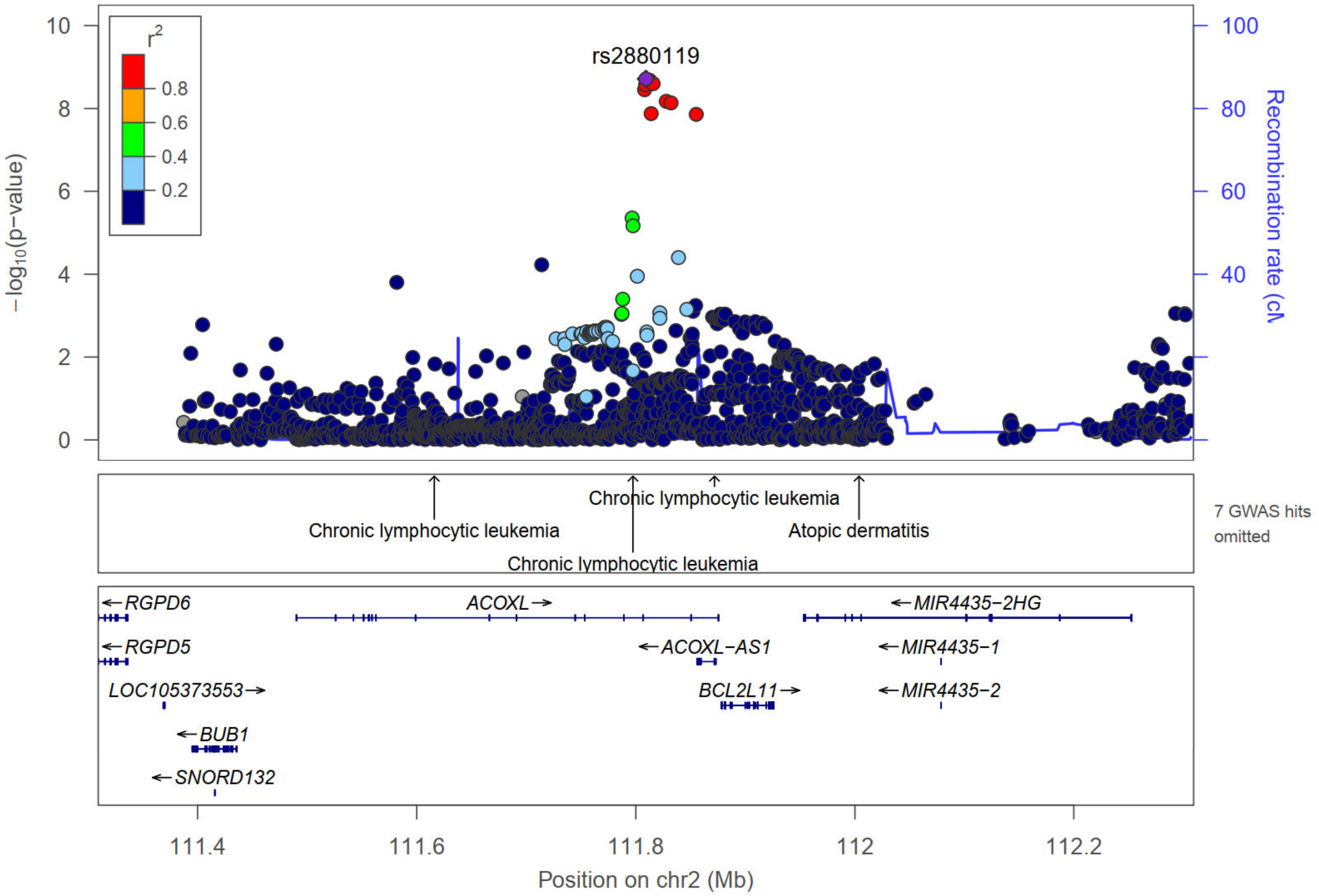

rs2880119
r2
0.8
0.6
0.4
0.2
−log10(p−value)
Recombination rate (cM
10
8
6
4
2
0
100
80
60
40
Chronic lymphocytic leukemia
Chronic lymphocytic leukemia
Atopic dermatitis
Chronic lymphocytic leukemia
7 GWAS hits omitted
RGPD6
ACOXL
MIR4435−2HG
RGPD5
ACOXL−AS1
MIR4435−1
LOC105373553
BCL2L11
MIR4435−2
BUB1
SNORD132
111.4
111.6
111.8
112
112.2
Position on chr2 (Mb)

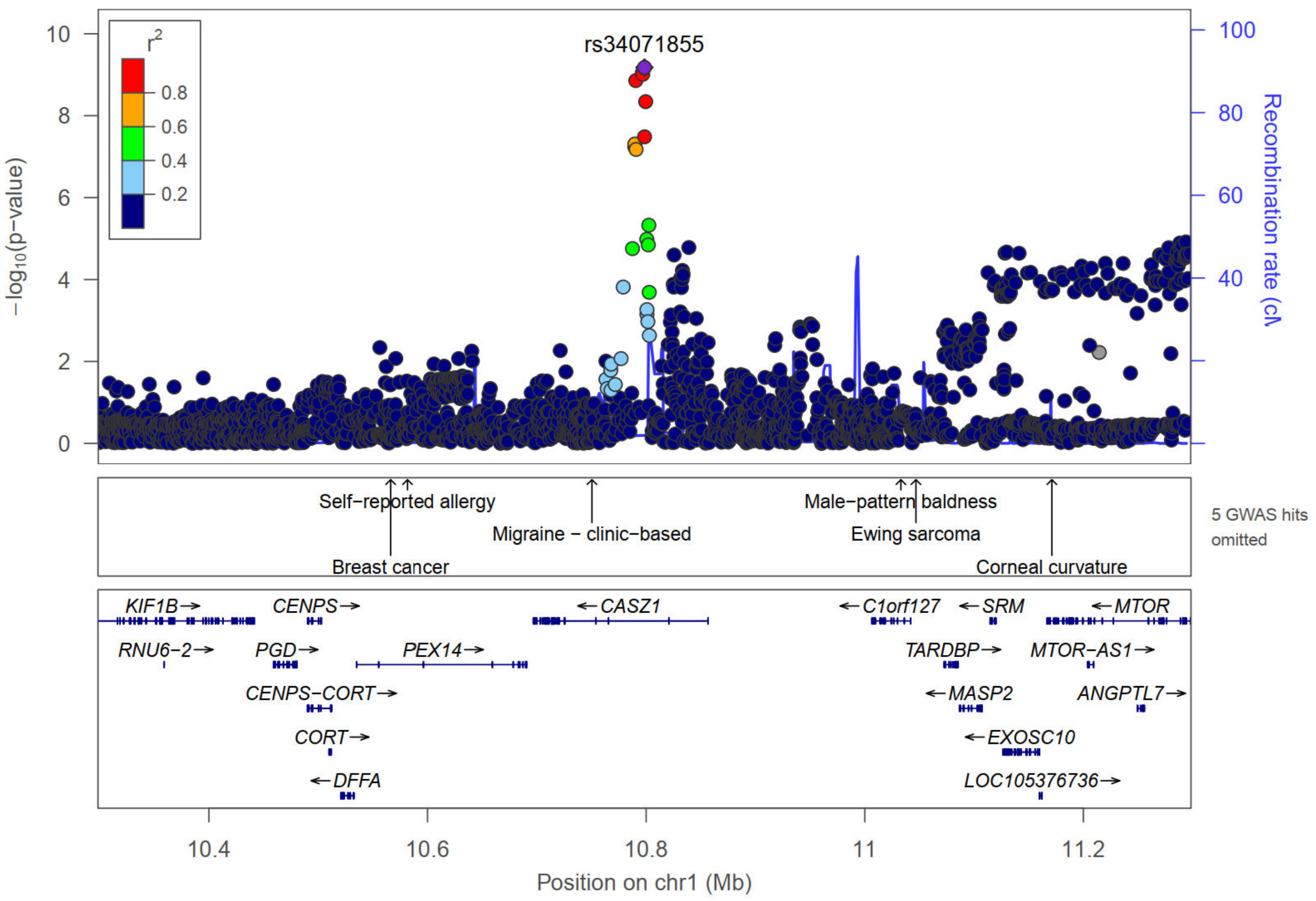

rs34071855
r2
0.8
0.6
0.4
0.2
−log10(p−value)
Recombination rate (cM
Self−reported allergy
Migraine − clinic−based
Male−pattern baldness
Ewing sarcoma
Breast cancer
Corneal curvature
5 GWAS hits omitted
KIF1B→
CENPS→
←CASZ1
←C1orf127
←SRM
←MTOR
RNU6−2→
PGD→
PEX14→
TARDBP→
MTOR−AS1→
CENPS−CORT→
←MASP2
ANGPTL7→
CORT→
←EXOSC10
←DFFA
LOC105376736→
Position on chr1 (Mb)

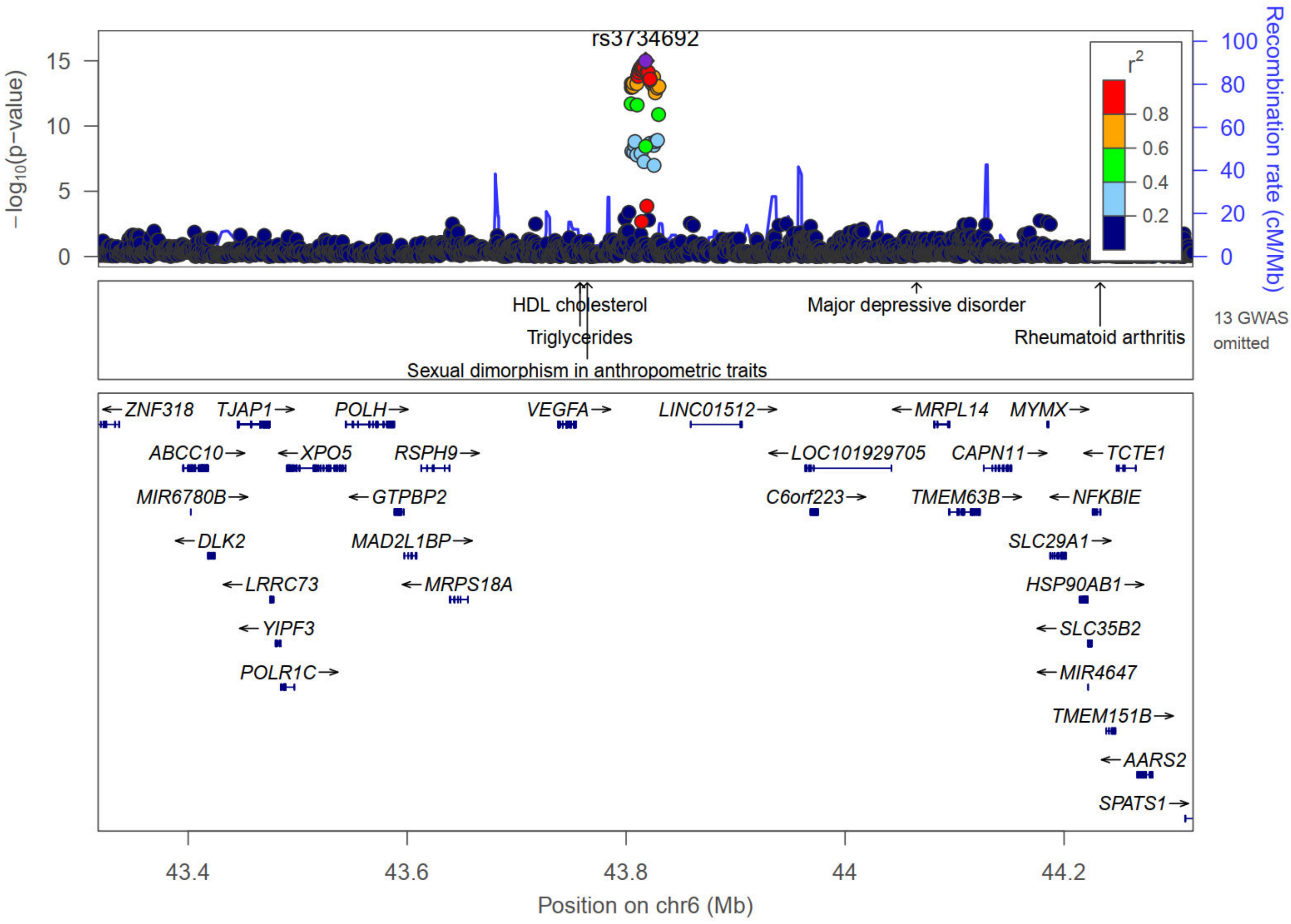

rs3734692
−log10(p−value)
Recombination rate (cM/Mb)
r2
0.8
0.6
0.4
0.2
HDL cholesterol
Triglycerides
Sexual dimorphism in anthropometric traits
Major depressive disorder
Rheumatoid arthritis
13 GWAS hits omitted
ZNF318
TJAP1
POLH
VEGFA
LINC01512
MRPL14
MYMX
ABCC10
XPO5
RSPH9
LOC101929705
CAPN11
TCTE1
MIR6780B
GTPBP2
C6orf223
TMEM63B
NFKBIE
DLK2
MAD2L1BP
SLC29A1
LRRC73
MRPS18A
HSP90AB1
YIPF3
SLC35B2
POLR1C
MIR4647
TMEM151B
AARS2
SPATS1
Position on chr6 (Mb)

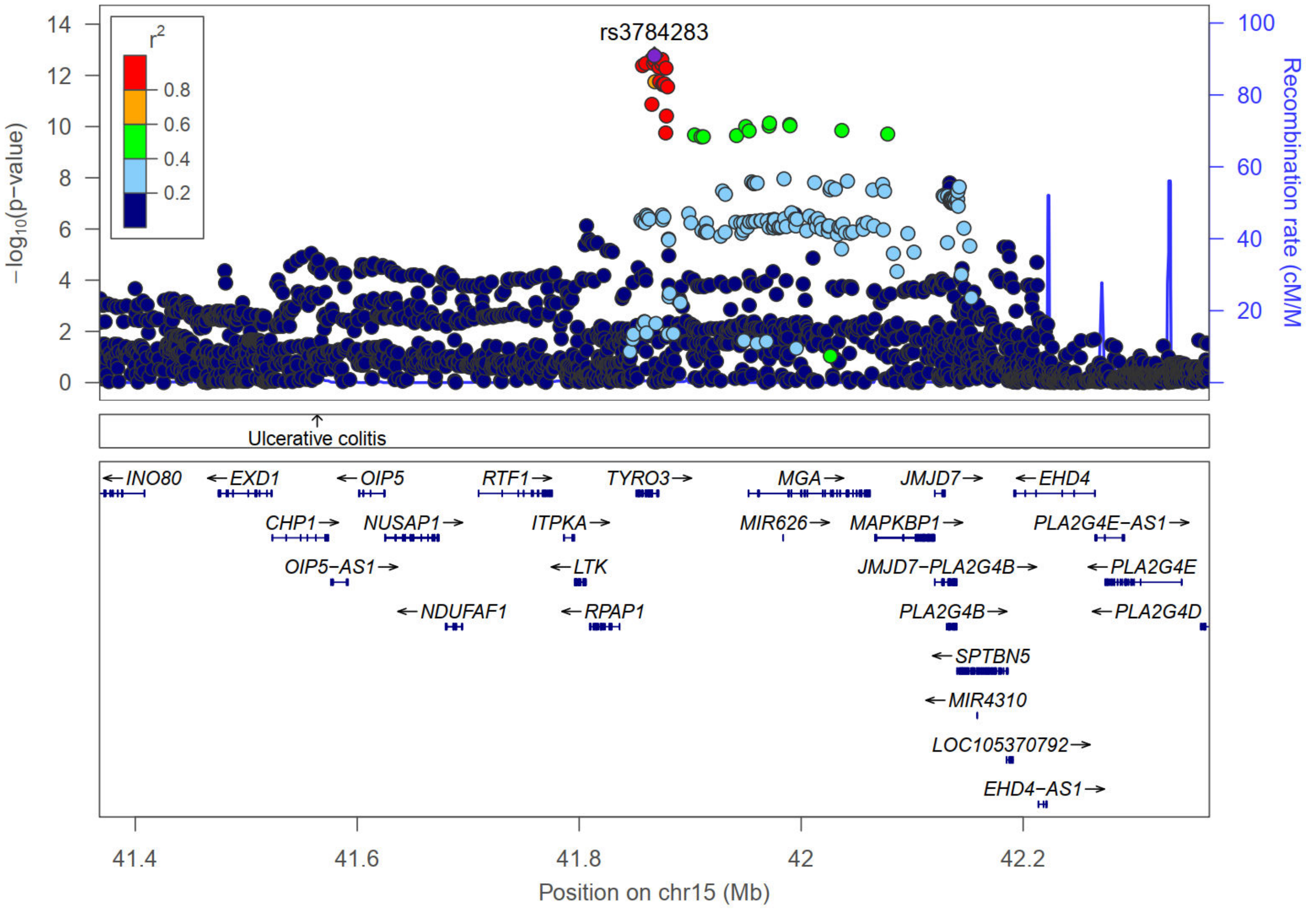

rs3784283
r2
0.8
0.6
0.4
0.2
−log10(p−value)
Recombination rate (cM/Mb)
Ulcerative colitis
INO80
EXD1
OIP5
RTF1
TYRO3
MGA
JMJD7
EHD4
CHP1
NUSAP1
ITPKA
MIR626
MAPKBP1
PLA2G4E−AS1
OIP5−AS1
LTK
JMJD7−PLA2G4B
PLA2G4E
NDUFAF1
RPAP1
PLA2G4B
PLA2G4D
SPTBN5
MIR4310
LOC105370792
EHD4−AS1
Position on chr15 (Mb)

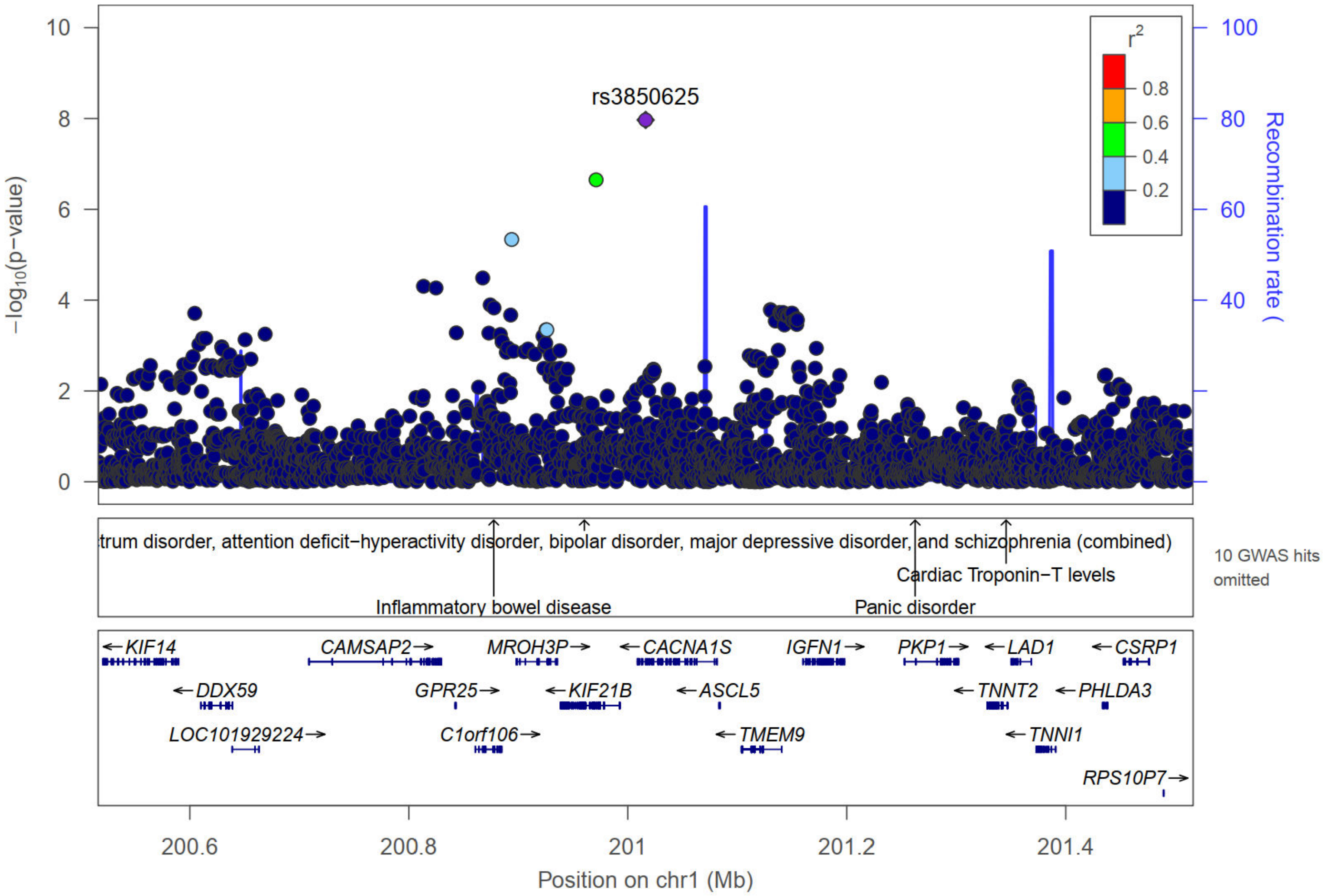

rs3850625
−log10(p−value)
Recombination rate (
r2
0.8
0.6
0.4
0.2
:trum disorder, attention deficit−hyperactivity disorder, bipolar disorder, major depressive disorder, and schizophrenia (combined)
Cardiac Troponin−T levels
Inflammatory bowel disease
Panic disorder
10 GWAS hits omitted
KIF14
CAMSAP2
MROH3P
CACNA1S
IGFN1
PKP1
LAD1
CSRP1
DDX59
GPR25
KIF21B
ASCL5
TNNT2
PHLDA3
LOC101929224
C1orf106
TMEM9
TNNI1
RPS10P7
200.6
200.8
201
201.2
201.4
Position on chr1 (Mb)

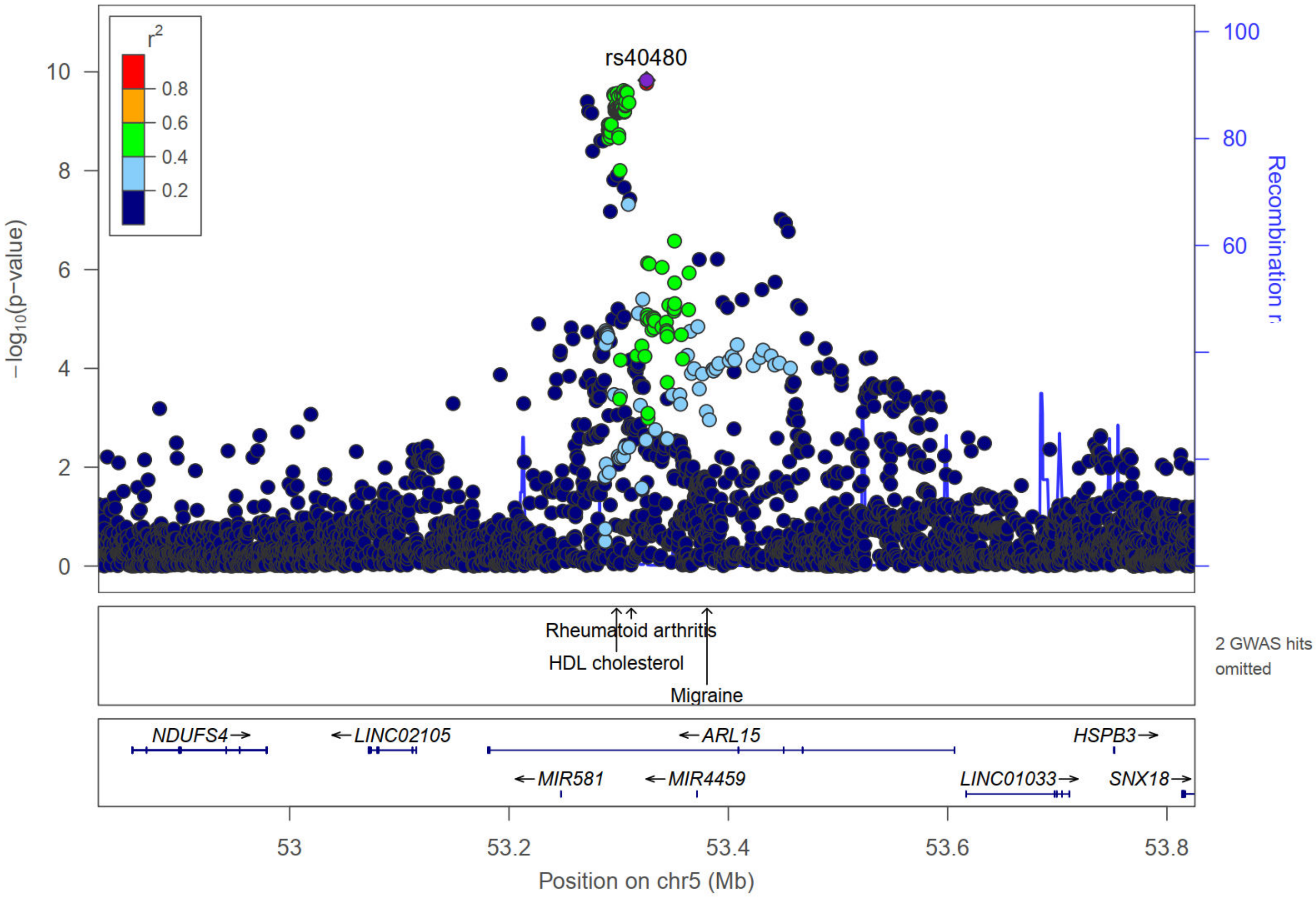
r2
0.8
0.6
0.4
0.2
rs40480
−log10(p−value)
Recombination r
100
80
60
Rheumatoid arthritis
HDL cholesterol
Migraine
2 GWAS hits omitted
NDUFS4→
←LINC02105
←ARL15
HSPB3→
←MIR581
←MIR4459
LINC01033→
SNX18→
53
53.2
53.4
53.6
53.8
Position on chr5 (Mb)

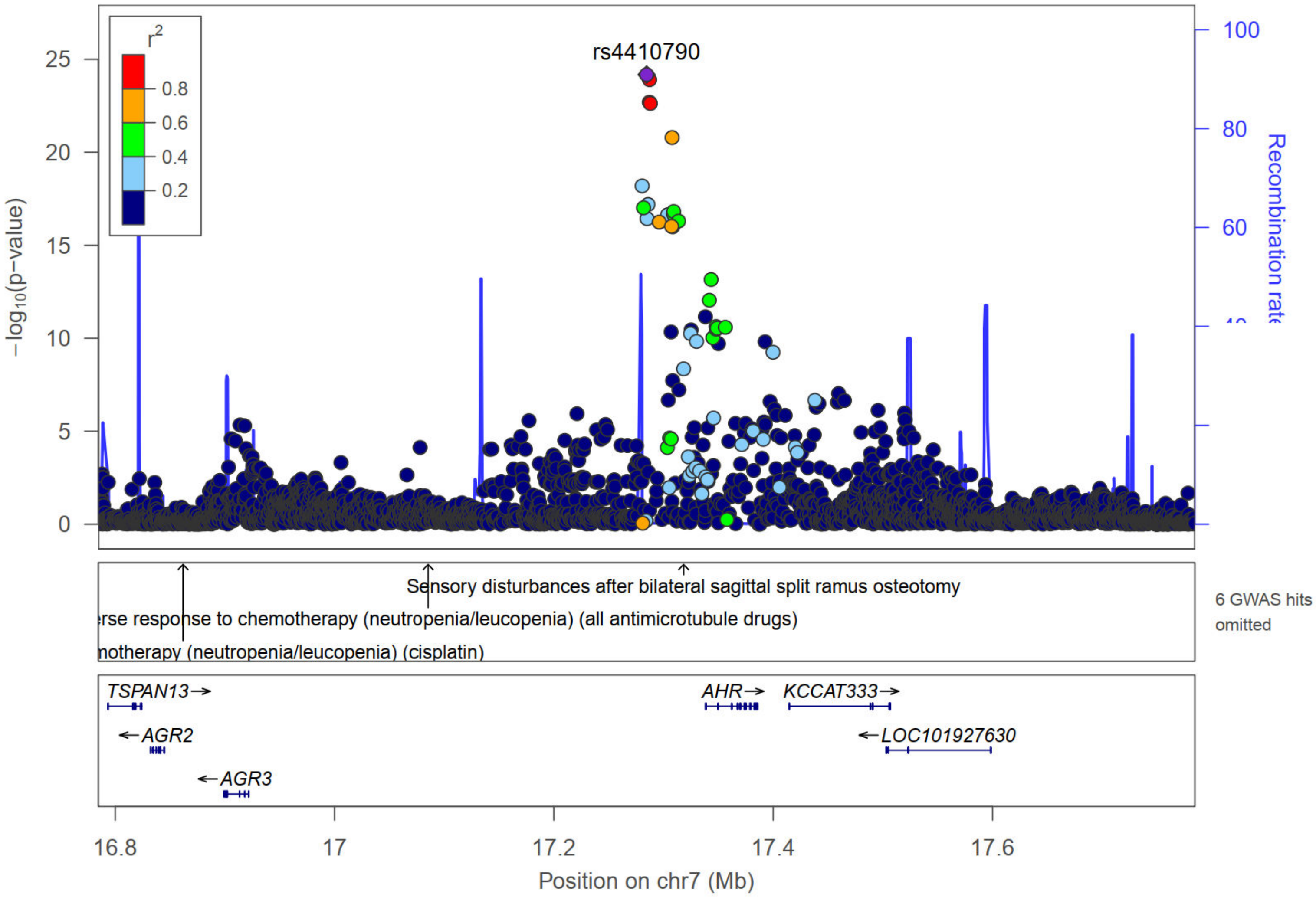

rs4410790
r2
0.8
0.6
0.4
0.2
−log10(p−value)
Recombination rate
Sensory disturbances after bilateral sagittal split ramus osteotomy
erse response to chemotherapy (neutropenia/leucopenia) (all antimicrotubule drugs)
notherapy (neutropenia/leucopenia) (cisplatin)
6 GWAS hits omitted
TSPAN13→
←AGR2
←AGR3
AHR→
KCCAT333→
←LOC101927630
Position on chr7 (Mb)

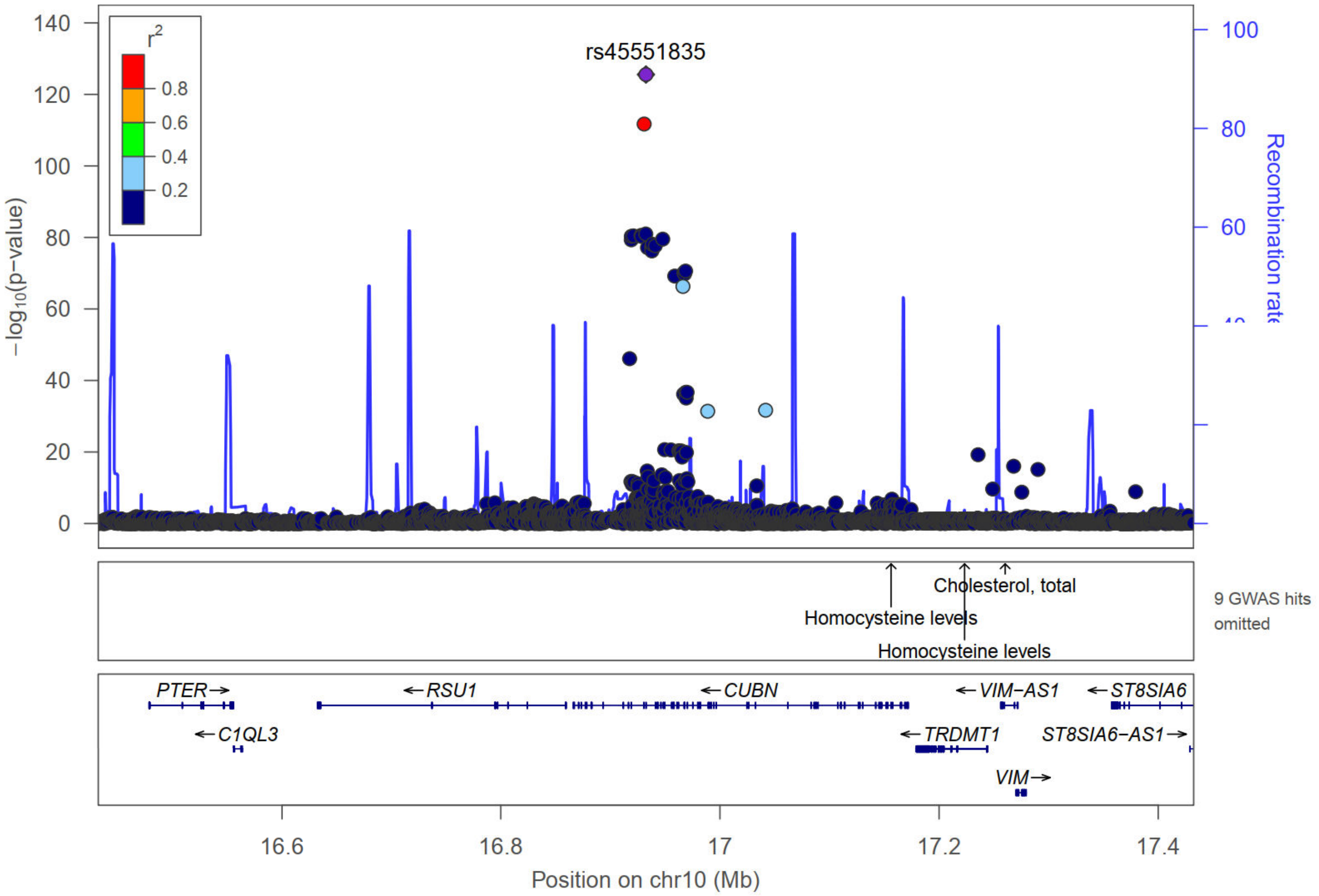

rs45551835
r2
0.8
0.6
0.4
0.2
140
120
100
80
60
40
20
0
−log10(p−value)
100
80
60
Recombination rate
Cholesterol, total
Homocysteine levels
Homocysteine levels
9 GWAS hits omitted
PTER→
←RSU1
←CUBN
←VIM−AS1
←ST8SIA6
←C1QL3
←TRDMT1
ST8SIA6−AS1→
VIM→
16.6
16.8
17
17.2
17.4
Position on chr10 (Mb)

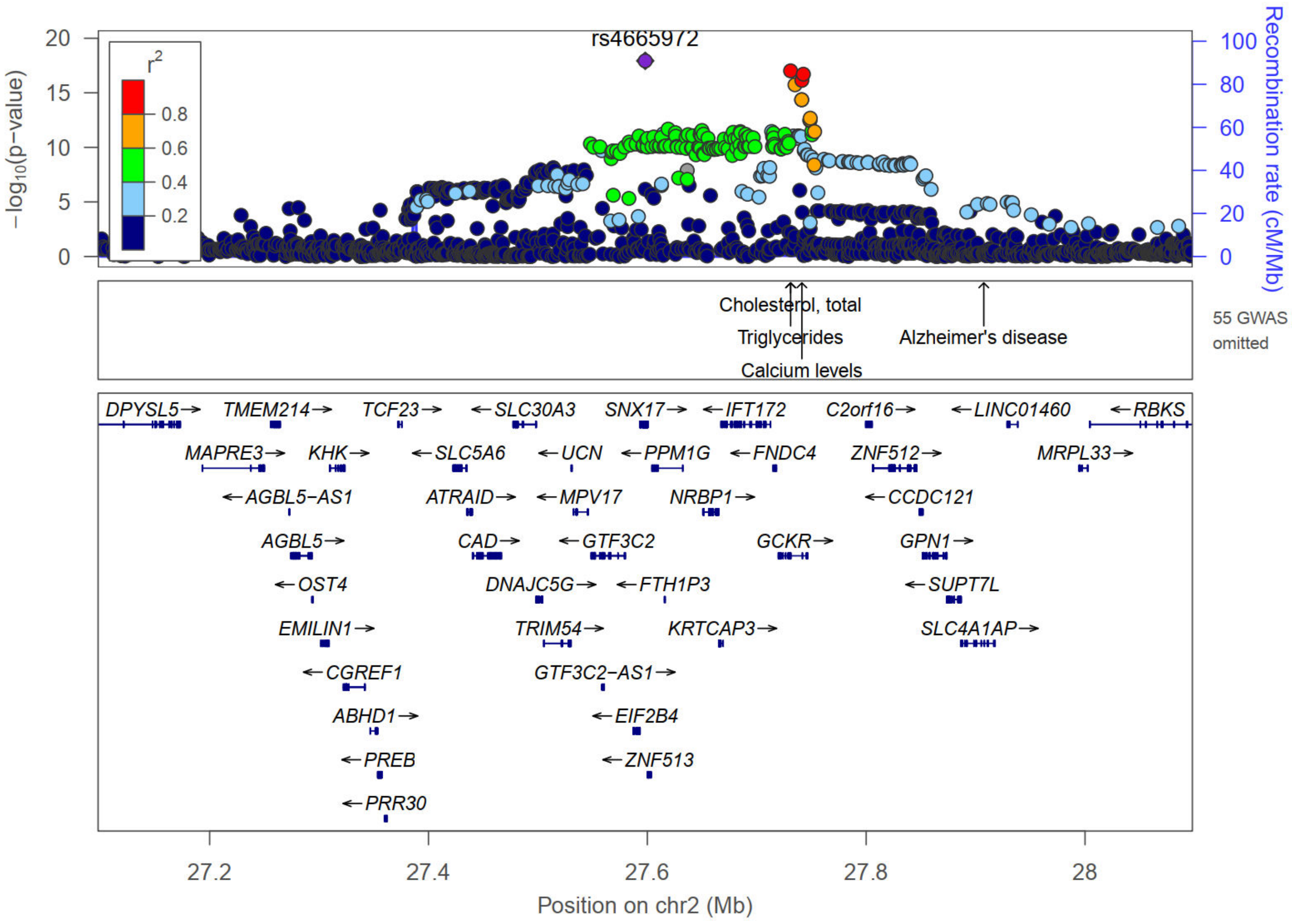

rs4665972
r2
0.8
0.6
0.4
0.2
−log10(p−value)
Recombination rate (cM/Mb)
Cholesterol, total
Triglycerides
Calcium levels
Alzheimer's disease
55 GWAS hits omitted
DPYSL5 TMEM214 TCF23 SLC30A3 SNX17 IFT172 C2orf16 LINC01460 RBKS
MAPRE3 KHK SLC5A6 UCN PPM1G FNDC4 ZNF512 MRPL33
AGBL5−AS1 ATRAID MPV17 NRBP1 CCDC121
AGBL5 CAD GTF3C2 GCKR GPN1
OST4 DNAJC5G FTH1P3 SUPT7L
EMILIN1 TRIM54 KRTCAP3 SLC4A1AP
CGREF1 GTF3C2−AS1
ABHD1 EIF2B4
PREB ZNF513
PRR30
27.2 27.4 27.6 27.8 28
Position on chr2 (Mb)

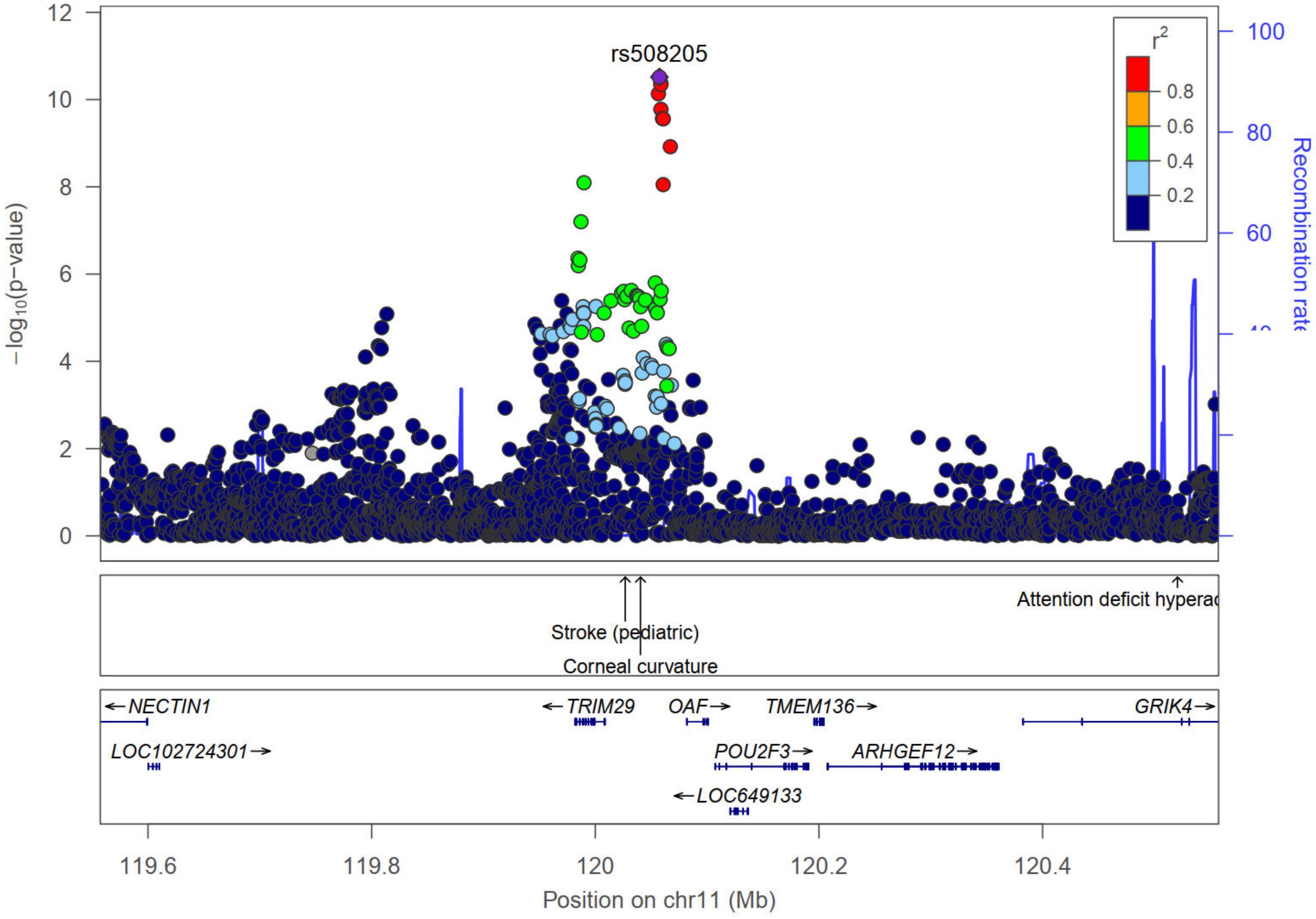

rs508205
−log10(p−value)
Recombination rate
r2
0.8
0.6
0.4
0.2
Stroke (pediatric)
Corneal curvature
Attention deficit hyperac
NECTIN1
LOC102724301
TRIM29
OAF
TMEM136
POU2F3
ARHGEF12
LOC649133
GRIK4
119.6
119.8
120
120.2
120.4
Position on chr11 (Mb)

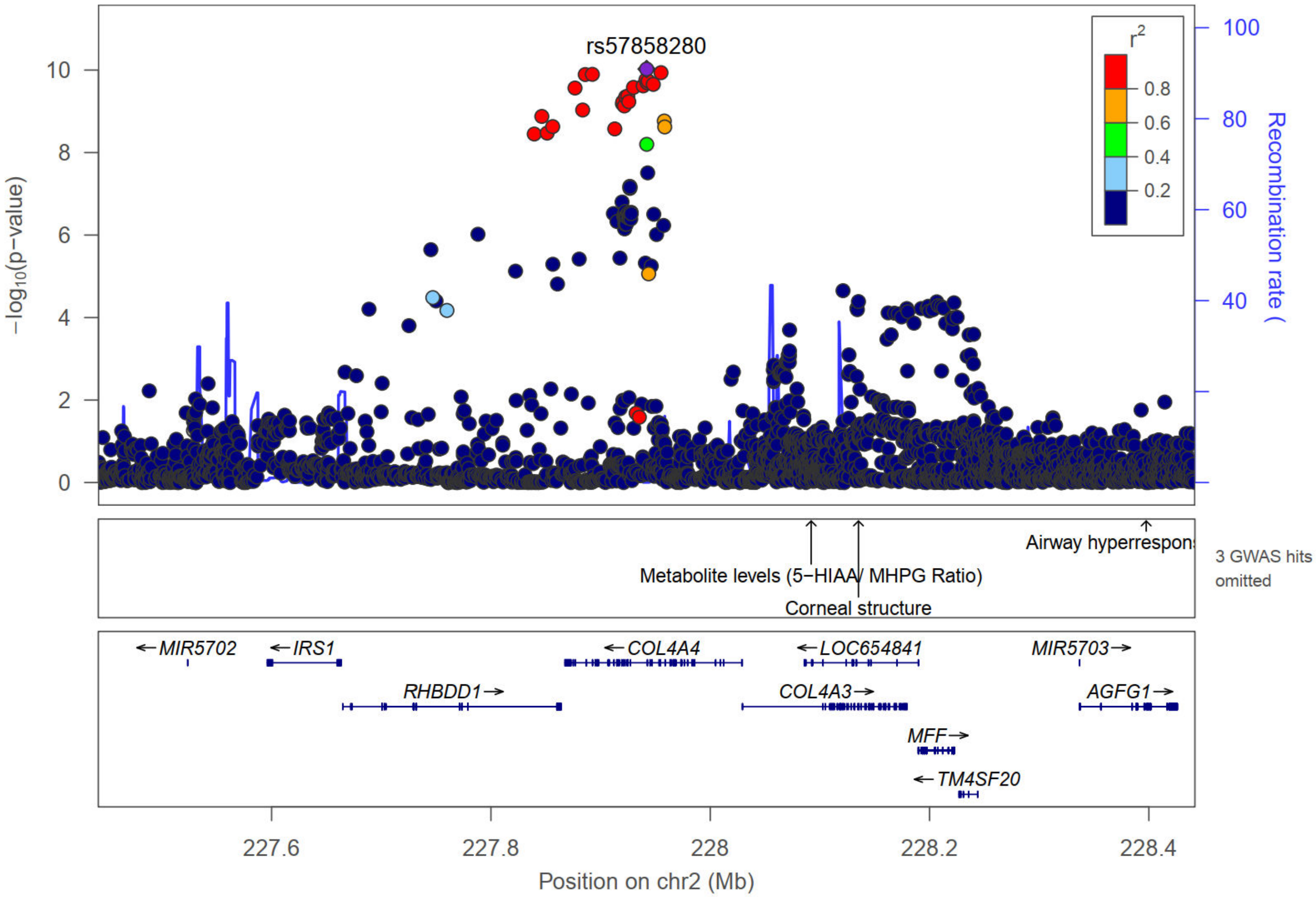

rs57858280
r2
0.8
0.6
0.4
0.2
−log10(p−value)
Recombination rate (
Metabolite levels (5−HIAA/ MHPG Ratio)
Corneal structure
Airway hyperrespon
3 GWAS hits omitted
MIR5702
IRS1
COL4A4
LOC654841
MIR5703
RHBDD1
COL4A3
AGFG1
MFF
TM4SF20
227.6
227.8
228
228.2
228.4
Position on chr2 (Mb)

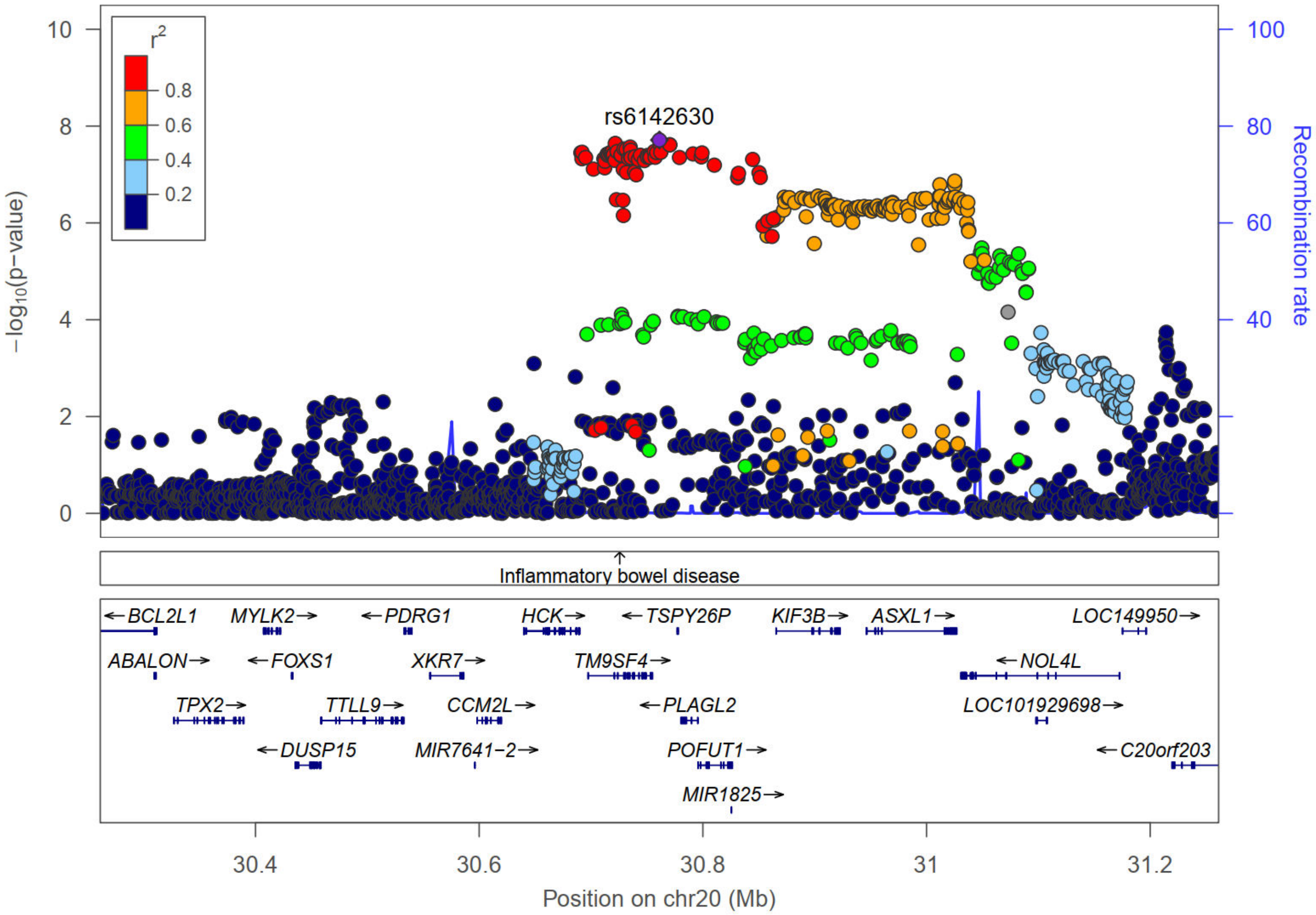

rs6142630
r2
0.8
0.6
0.4
0.2
−log10(p−value)
Recombination rate
Inflammatory bowel disease
BCL2L1
MYLK2
PDRG1
HCK
TSPY26P
KIF3B
ASXL1
LOC149950
ABALON
FOXS1
XKR7
TM9SF4
NOL4L
TPX2
TTLL9
CCM2L
PLAGL2
LOC101929698
DUSP15
MIR7641−2
POFUT1
C20orf203
MIR1825
Position on chr20 (Mb)

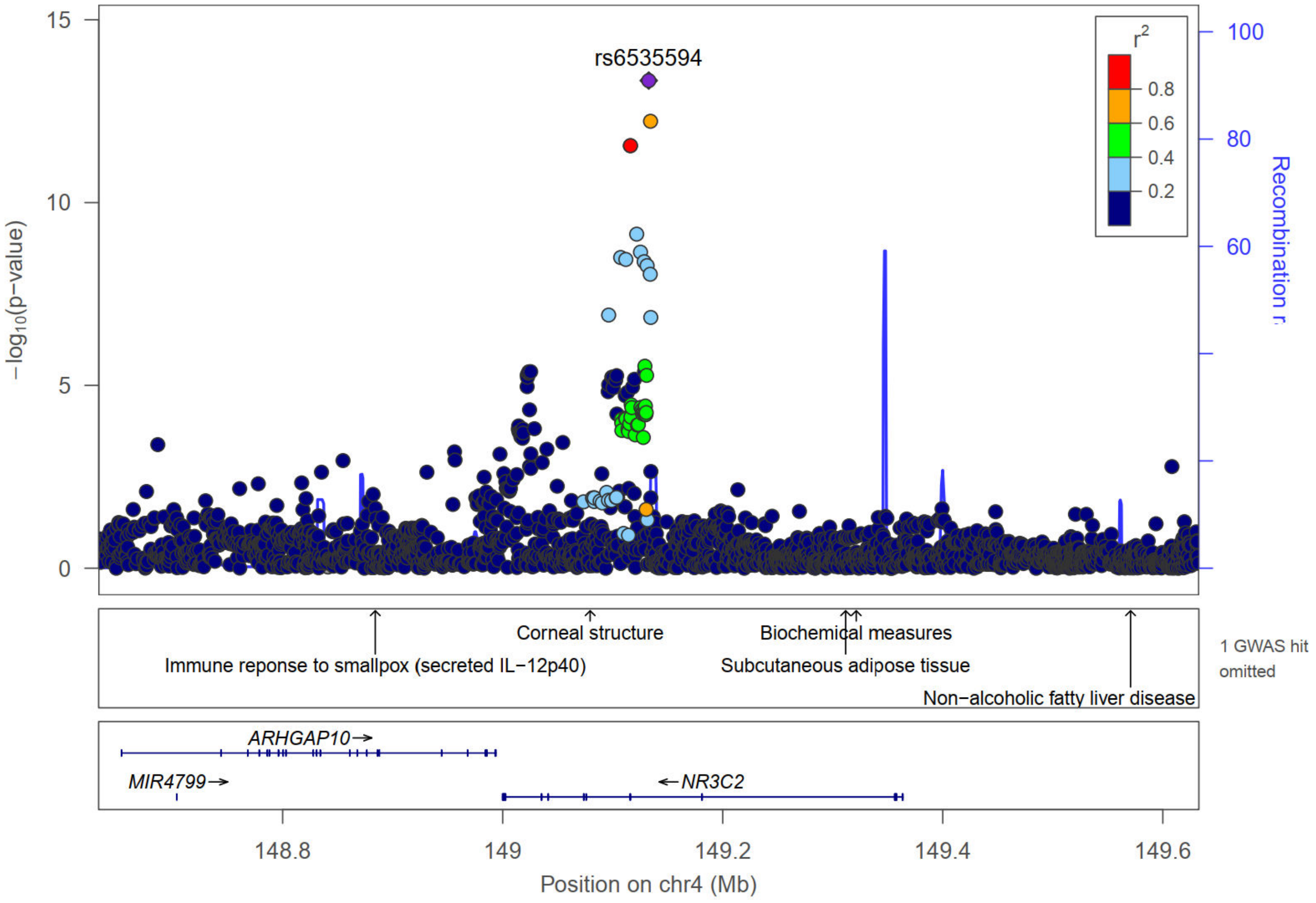
rs6535594
−log10(p−value)
Recombination r
r2
0.8
0.6
0.4
0.2
15
10
5
0
100
80
60
Corneal structure
Biochemical measures
Immune reponse to smallpox (secreted IL−12p40)
Subcutaneous adipose tissue
Non−alcoholic fatty liver disease
1 GWAS hit omitted
ARHGAP10→
MIR4799→
←NR3C2
148.8
149
149.2
149.4
149.6
Position on chr4 (Mb)

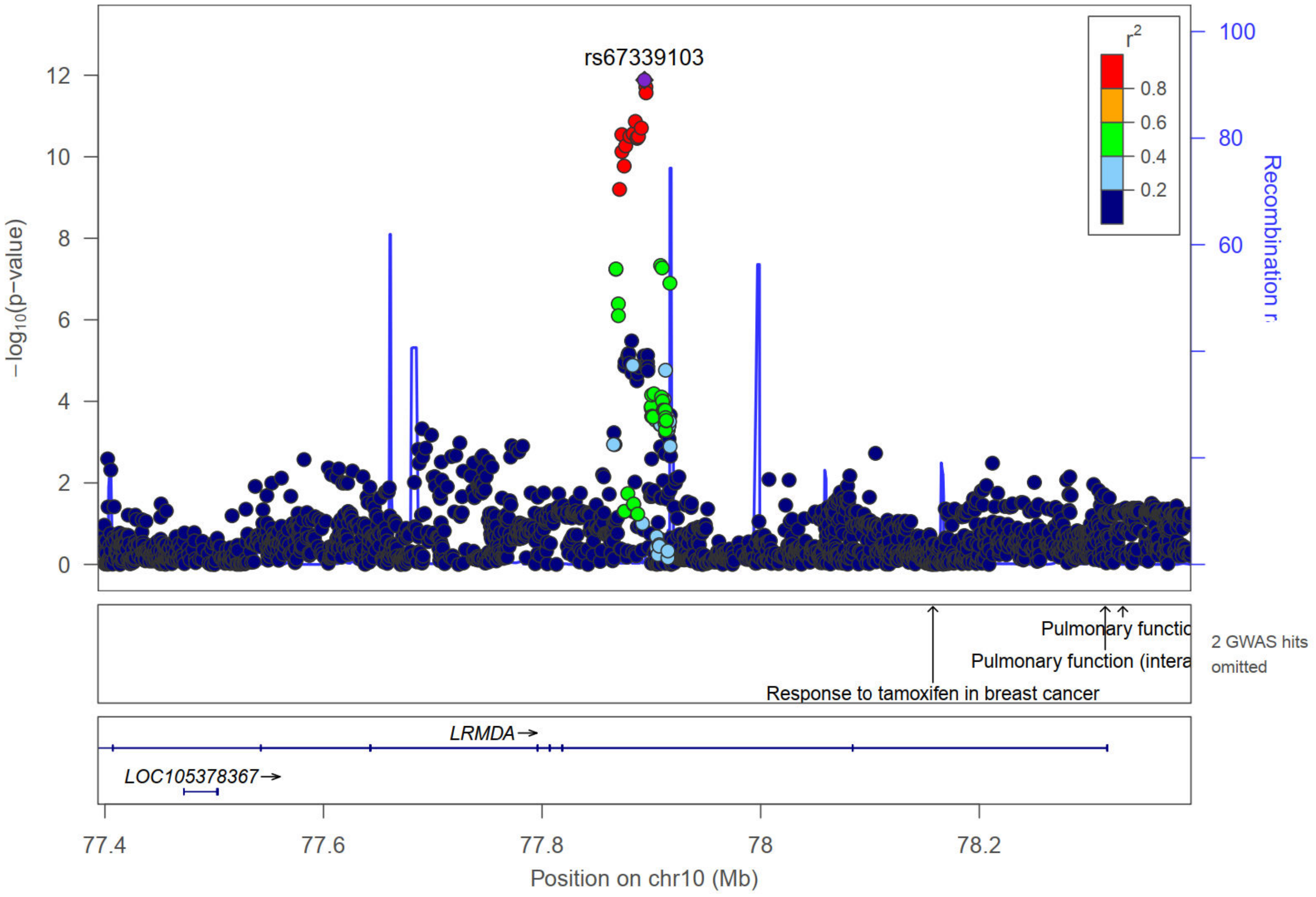

rs67339103
r2
0.8
0.6
0.4
0.2
−log10(p−value)
Recombination r.
Pulmonary functio
Pulmonary function (intera
Response to tamoxifen in breast cancer
2 GWAS hits omitted
LRMDA→
LOC105378367→
77.4
77.6
77.8
78
78.2
Position on chr10 (Mb)

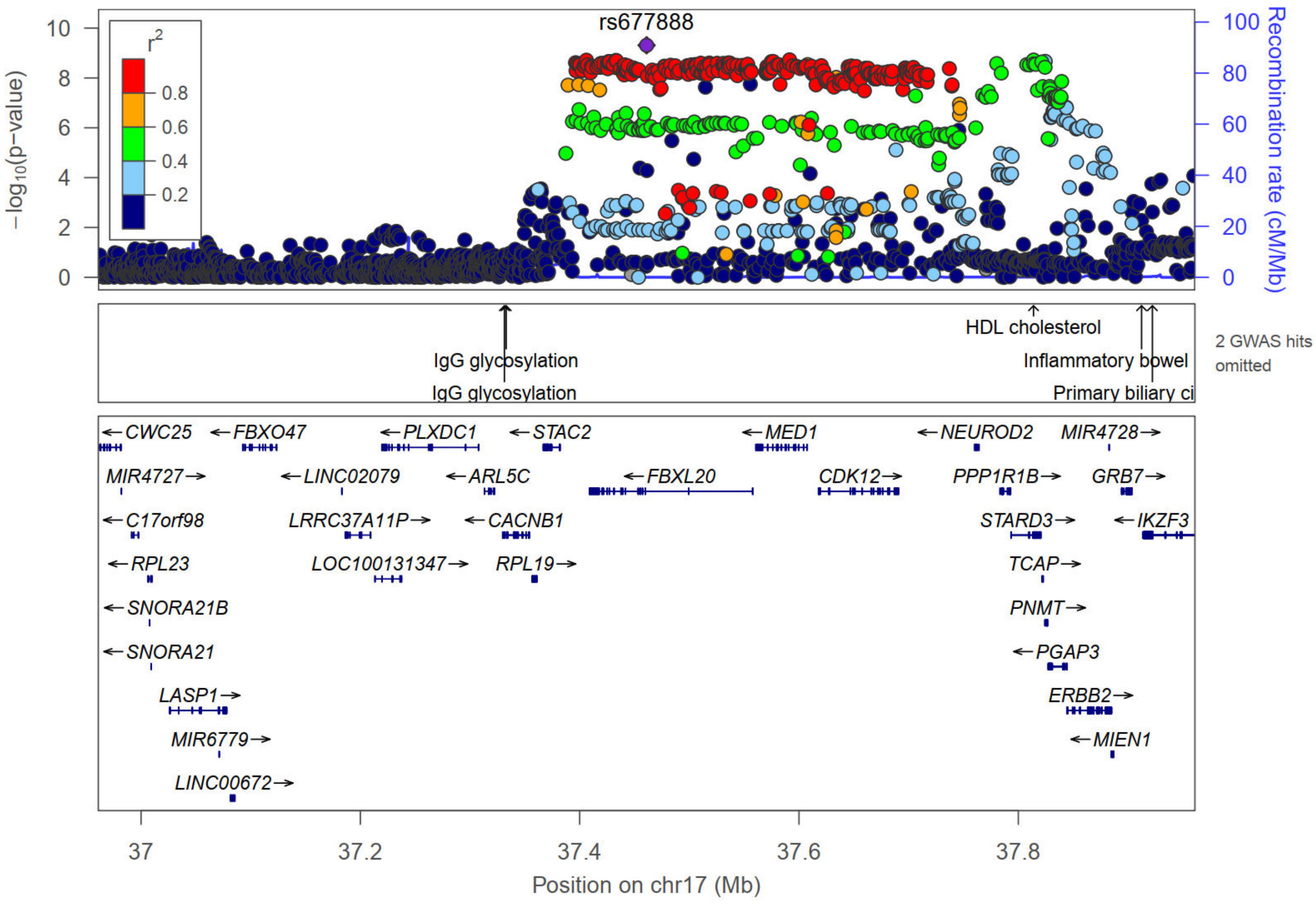
rs677888
r²
0.8
0.6
0.4
0.2
−log10(p−value)
Recombination rate (cM/Mb)
HDL cholesterol
IgG glycosylation
Inflammatory bowel
IgG glycosylation
Primary biliary ci
2 GWAS hits omitted
CWC25
FBXO47
PLXDC1
STAC2
MED1
NEUROD2
MIR4728
MIR4727
LINC02079
ARL5C
FBXL20
CDK12
PPP1R1B
GRB7
C17orf98
LRRC37A11P
CACNB1
STARD3
IKZF3
RPL23
LOC100131347
RPL19
TCAP
SNORA21B
PNMT
SNORA21
PGAP3
LASP1
ERBB2
MIR6779
MIEN1
LINC00672
37
37.2
37.4
37.6
37.8
Position on chr17 (Mb)

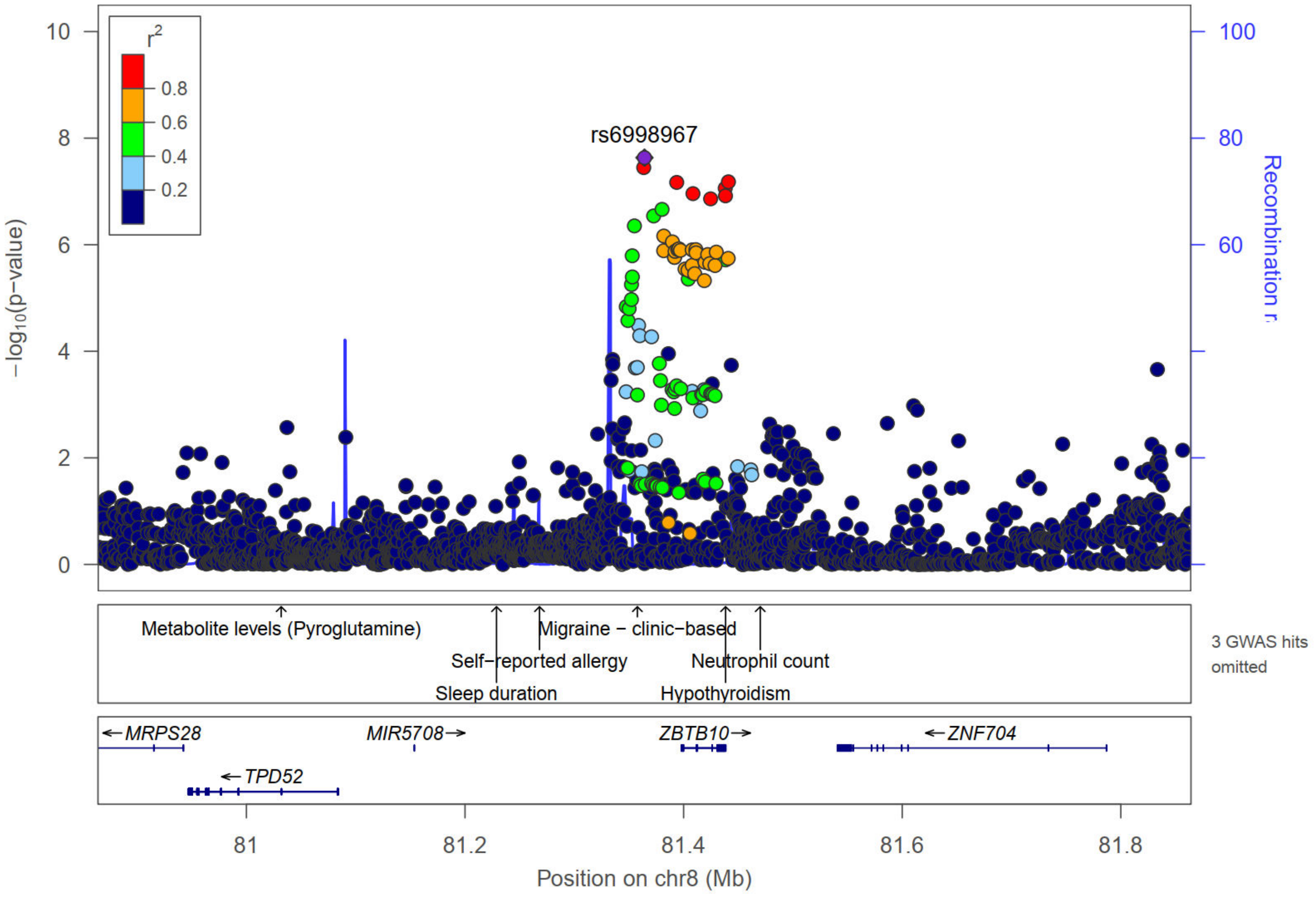

r2
0.8
0.6
0.4
0.2
rs6998967
10
8
6
4
2
0
−log10(p−value)
100
80
60
Recombination r
Metabolite levels (Pyroglutamine)
Self−reported allergy
Sleep duration
Migraine − clinic−based
Neutrophil count
Hypothyroidism
3 GWAS hits omitted
←MRPS28
←TPD52
MIR5708→
ZBTB10→
←ZNF704
81
81.2
81.4
81.6
81.8
Position on chr8 (Mb)

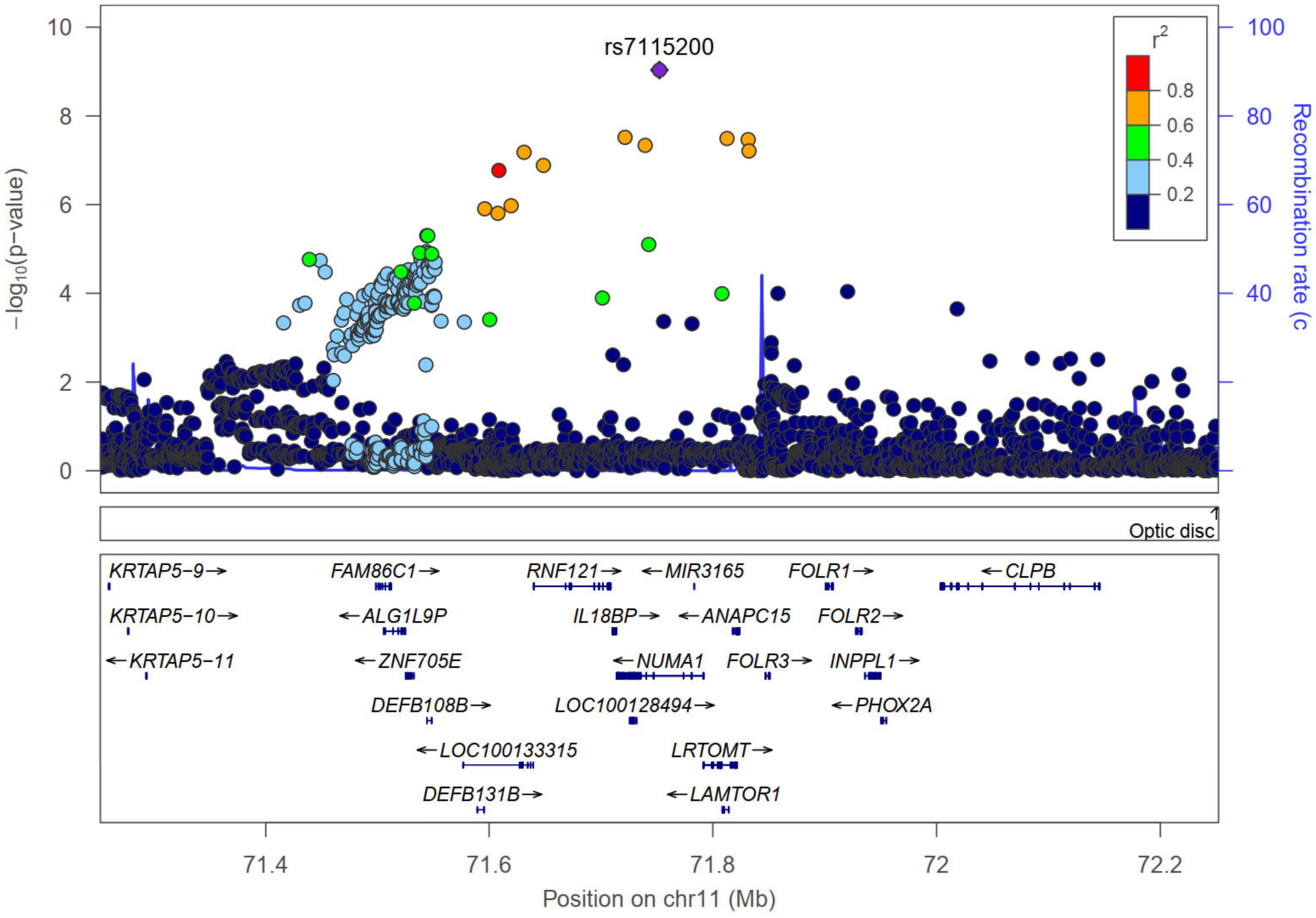

rs7115200
r2
0.8
0.6
0.4
0.2
−log10(p−value)
Recombination rate (c
Optic disc
KRTAP5−9→
KRTAP5−10→
←KRTAP5−11
FAM86C1→
←ALG1L9P
←ZNF705E
DEFB108B→
←LOC100133315
DEFB131B→
RNF121→
IL18BP→
←NUMA1
LOC100128494→
←MIR3165
←ANAPC15
FOLR3→
LRTOMT→
←LAMTOR1
FOLR1→
FOLR2→
INPPL1→
←PHOX2A
←CLPB
Position on chr11 (Mb)

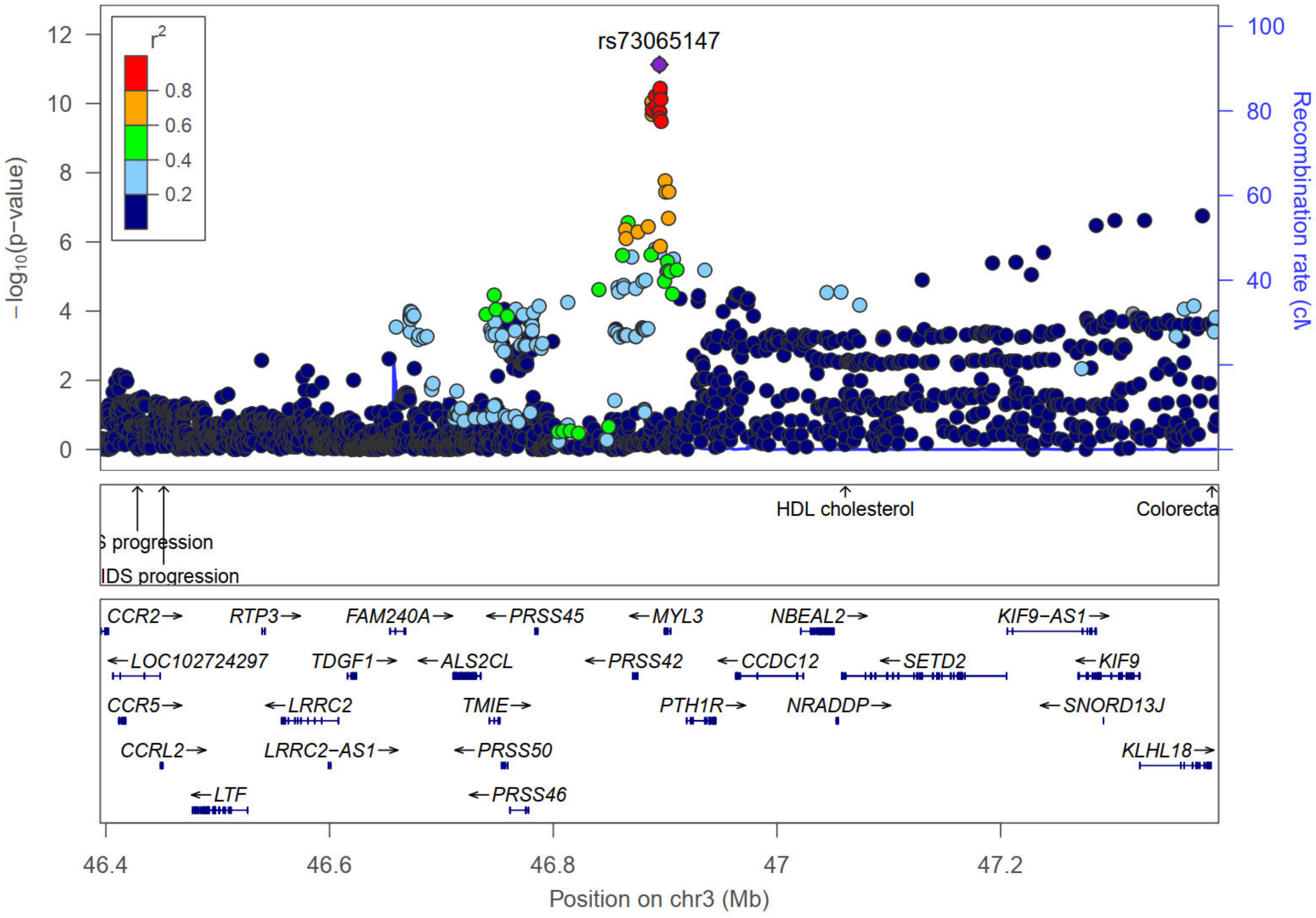

rs73065147
r2
0.8
0.6
0.4
0.2
−log10(p−value)
Recombination rate (cM
HDL cholesterol
Colorecta
S progression
IDS progression
CCR2→
RTP3→
FAM240A→
←PRSS45
←MYL3
NBEAL2→
KIF9−AS1→
←LOC102724297
TDGF1→
←ALS2CL
←PRSS42
←CCDC12
←SETD2
←KIF9
CCR5→
←LRRC2
TMIE→
PTH1R→
NRADDP→
←SNORD13J
CCRL2→
LRRC2−AS1→
←PRSS50
KLHL18→
←LTF
←PRSS46
Position on chr3 (Mb)

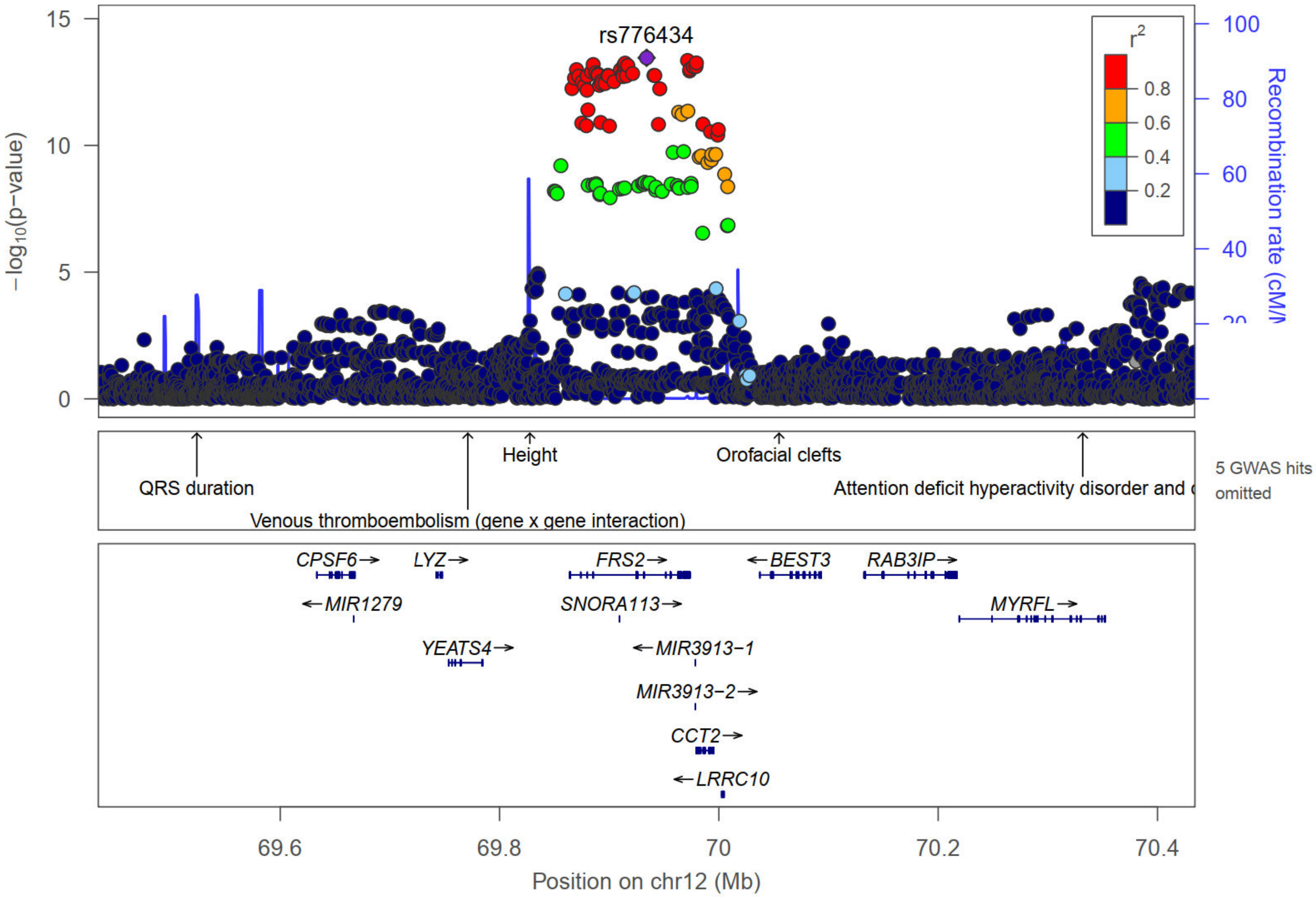

rs776434
−log10(p−value)
Recombination rate (cM/Mb)
r2
0.8
0.6
0.4
0.2
QRS duration
Venous thromboembolism (gene x gene interaction)
Height
Orofacial clefts
Attention deficit hyperactivity disorder and
5 GWAS hits omitted
CPSF6
LYZ
FRS2
BEST3
RAB3IP
MIR1279
SNORA113
MYRFL
YEATS4
MIR3913−1
MIR3913−2
CCT2
LRRC10
69.6
69.8
70
70.2
70.4
Position on chr12 (Mb)

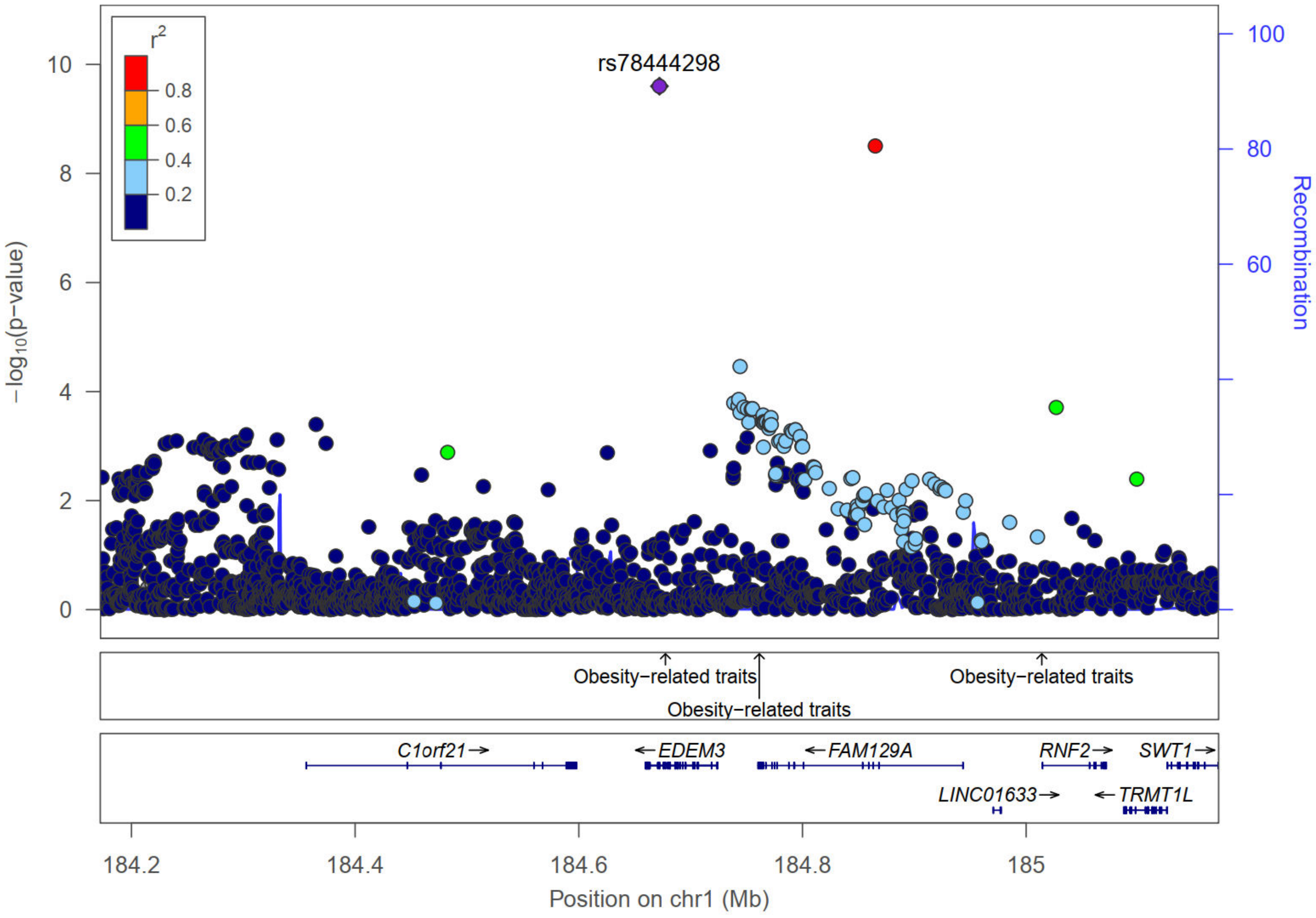

rs78444298
r²
0.8
0.6
0.4
0.2
−log10(p−value)
Recombination
Obesity−related traits
Obesity−related traits
Obesity−related traits
C1orf21→
←EDEM3
←FAM129A
RNF2→
SWT1→
LINC01633→
←TRMT1L
184.2
184.4
184.6
184.8
185
Position on chr1 (Mb)

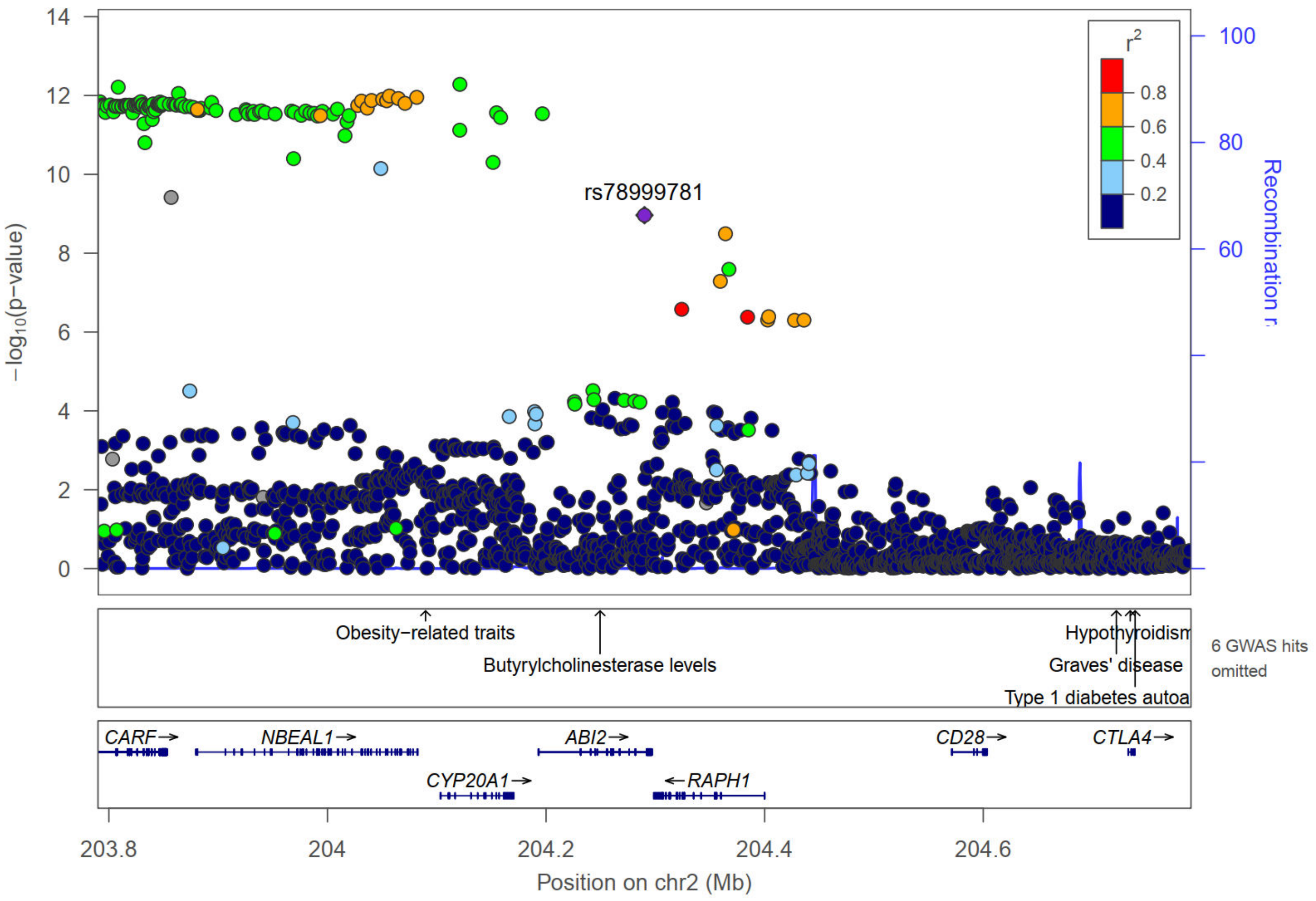

rs78999781
r²
0.8
0.6
0.4
0.2
−log10(p−value)
Recombination r
Obesity−related traits
Butyrylcholinesterase levels
Hypothyroidism
Graves' disease
Type 1 diabetes autoa
6 GWAS hits omitted
CARF
NBEAL1
ABI2
CD28
CTLA4
CYP20A1
RAPH1
203.8
204
204.2
204.4
204.6
Position on chr2 (Mb)

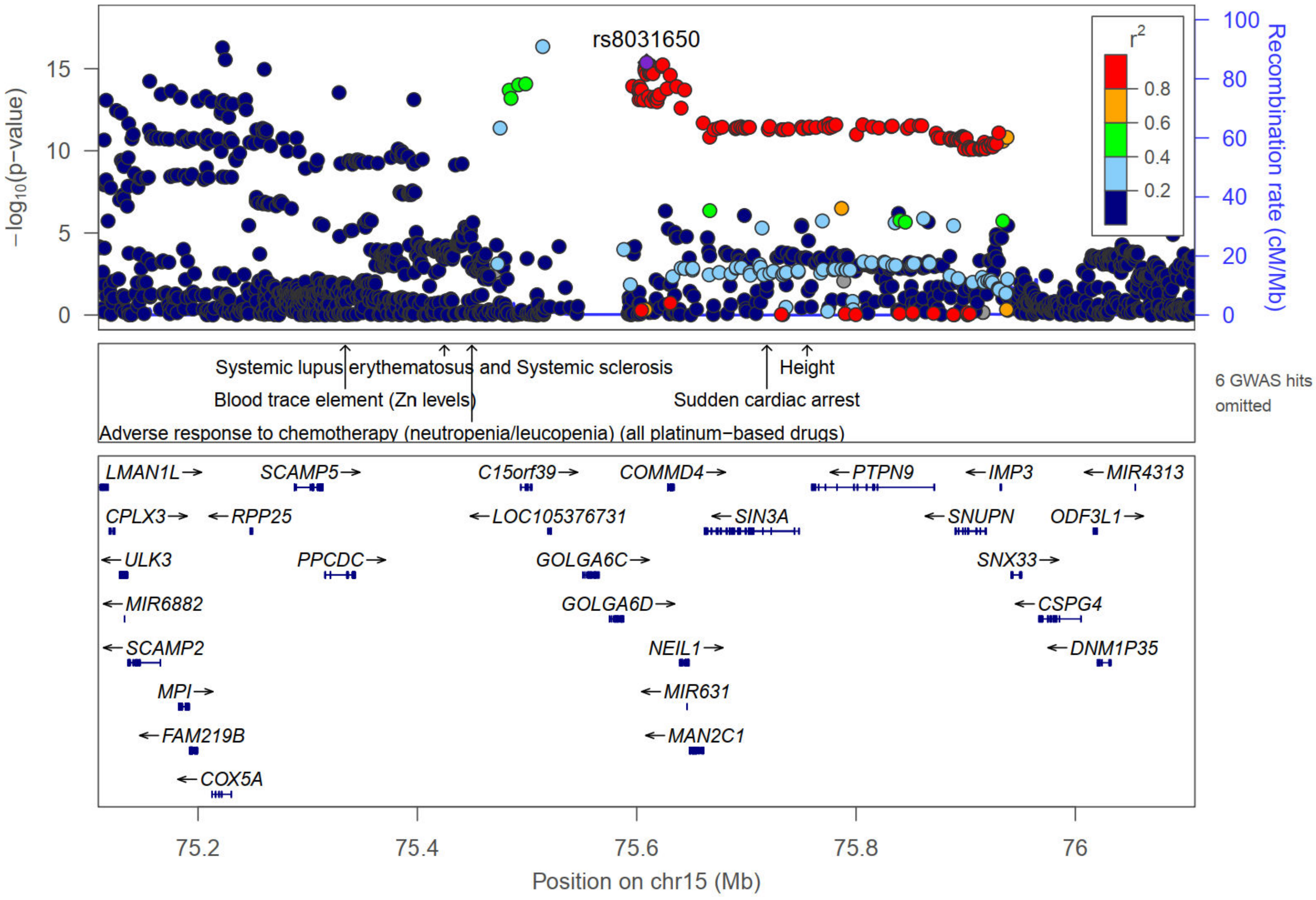

rs8031650
−log10(p−value)
Recombination rate (cM/Mb)
r2
0.8
0.6
0.4
0.2
Systemic lupus erythematosus and Systemic sclerosis
Height
Blood trace element (Zn levels)
Sudden cardiac arrest
Adverse response to chemotherapy (neutropenia/leucopenia) (all platinum−based drugs)
6 GWAS hits omitted
LMAN1L
SCAMP5
C15orf39
COMMD4
PTPN9
IMP3
MIR4313
CPLX3
RPP25
LOC105376731
SIN3A
SNUPN
ODF3L1
ULK3
PPCDC
GOLGA6C
SNX33
MIR6882
GOLGA6D
CSPG4
SCAMP2
NEIL1
DNM1P35
MPI
MIR631
FAM219B
MAN2C1
COX5A
75.2
75.4
75.6
75.8
76
Position on chr15 (Mb)

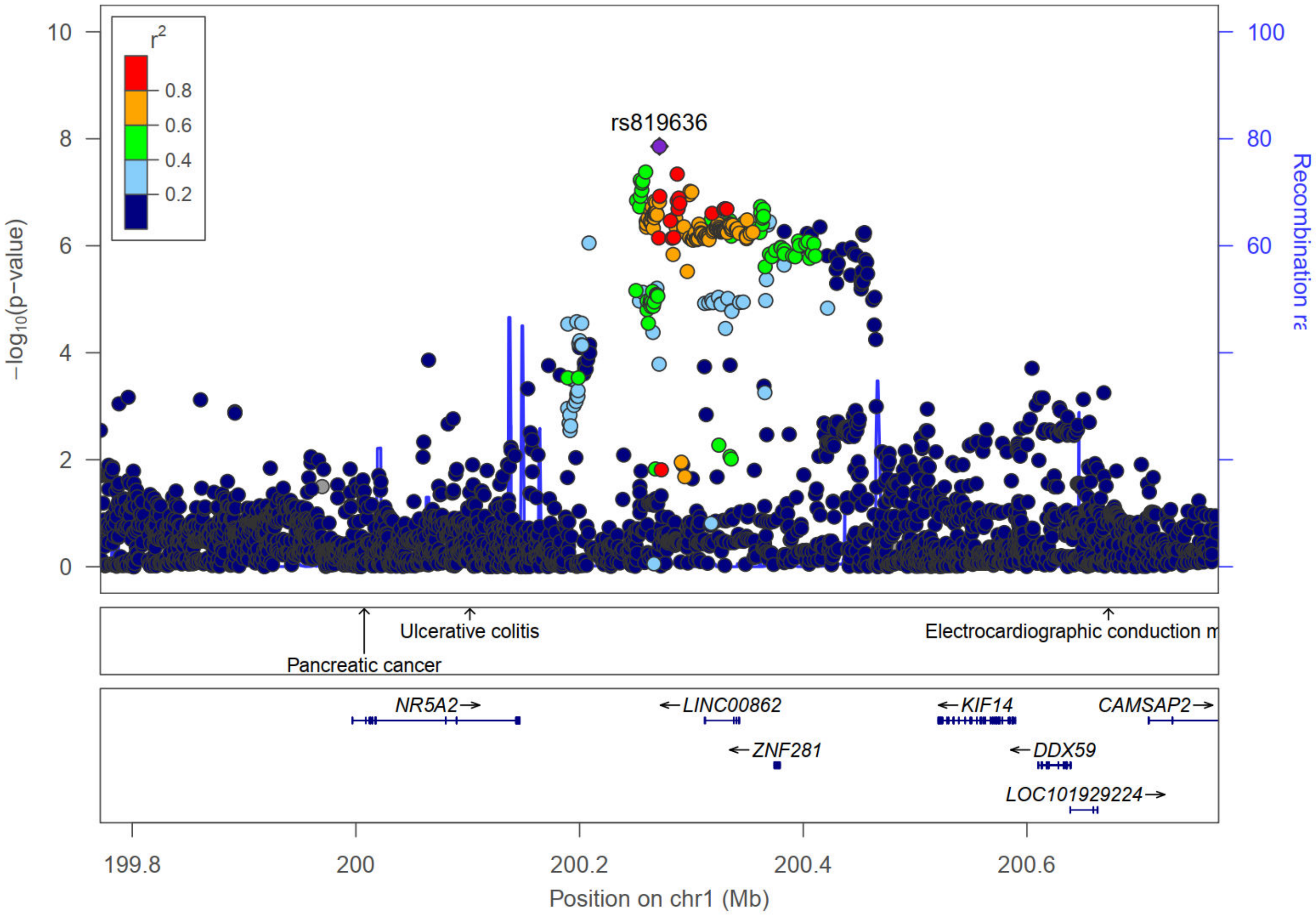

rs819636
r2
0.8
0.6
0.4
0.2
−log10(p−value)
Recombination ra
Ulcerative colitis
Pancreatic cancer
Electrocardiographic conduction m
NR5A2
LINC00862
ZNF281
KIF14
DDX59
CAMSAP2
LOC101929224
Position on chr1 (Mb)
199.8
200
200.2
200.4
200.6

Regional association plots are shown for all genome-wide significant index SNPs. The dots are plotted on the y-axis according to their meta-analysis association p-value, and colored according to their correlation r^2 with the index SNP estimated based on the 1000 Genomes EUR reference samples (gray for missing data). Plots were generated using the stand-alone version 1.3 of LocusZoom[2]. Genetic positions refer to GRCh37/hg19 coordinates.

Supplementary Figure 3: Expected number of discoveries at increasing GWAS discovery sample sizes.

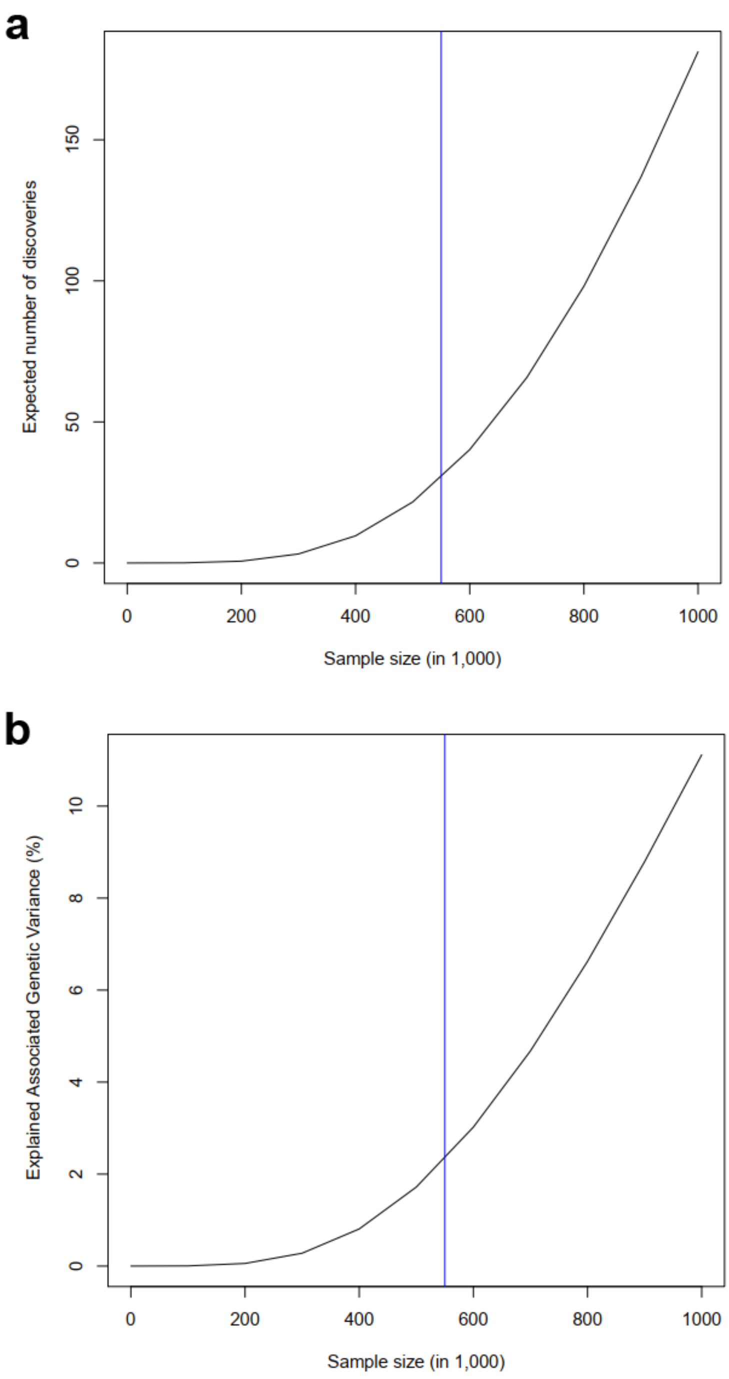

The panels show the estimate the number of expected discoveries (a) and the corresponding percentage of GWAS heritability explained (b) with increases in sample size using the summary statistics of our UACR trans-ethnic meta-analysis as input. The vertical blue line marks the number of predicted discoveries for UACR at the current sample size and may represent an underestimate because of many SNPs with small effects.

Supplementary Figure 4: Regional association plots for loci identified in trans-ethnic GWAS meta-analysis of UACR among individuals with diabetes

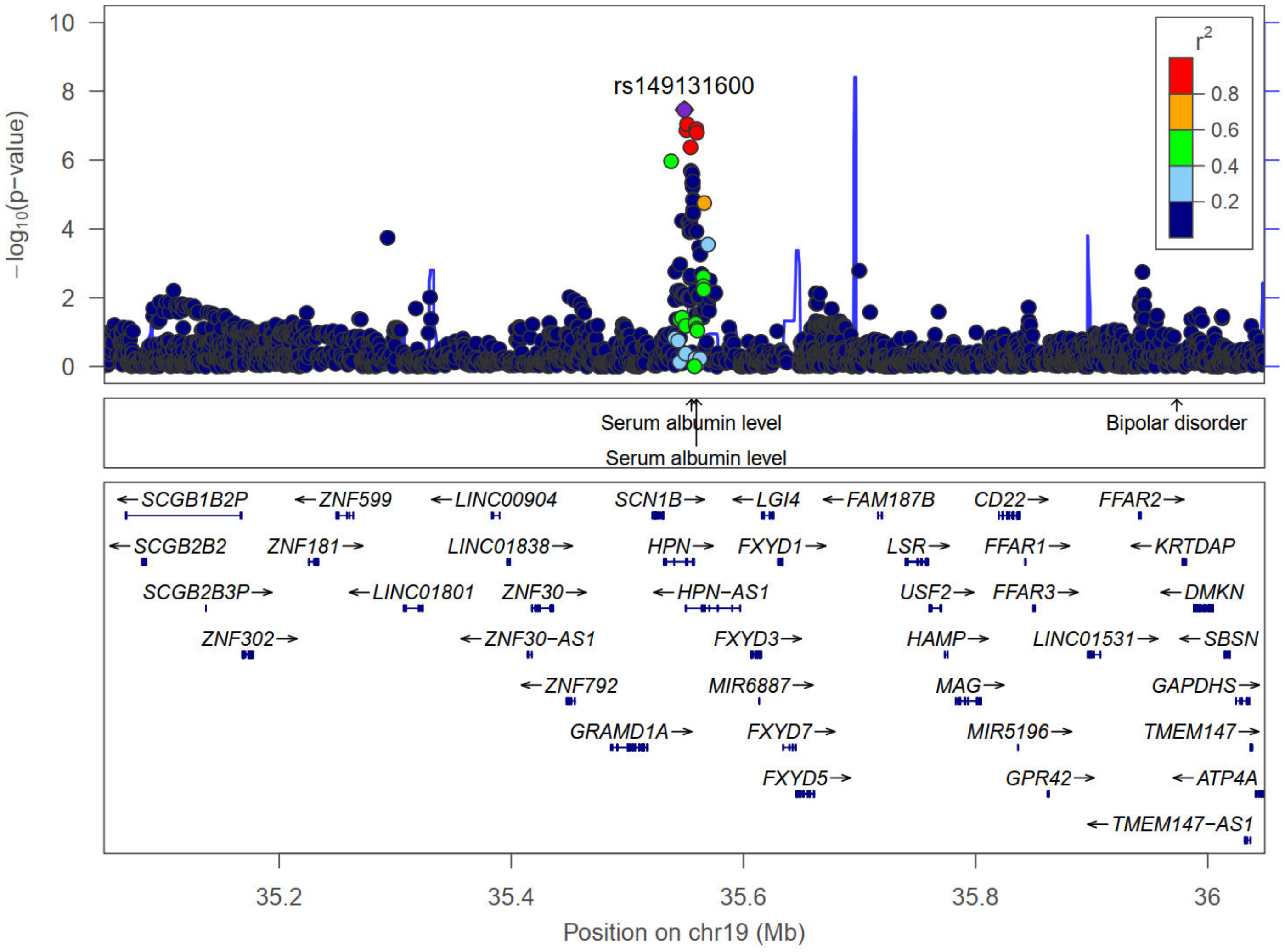

rs149131600
r2
0.8
0.6
0.4
0.2
−log10(p−value)
10
8
6
4
2
0
Serum albumin level
Serum albumin level
Bipolar disorder
←SCGB1B2P
←ZNF599
←LINC00904
SCN1B→
←LGI4
←FAM187B
CD22→
FFAR2→
←SCGB2B2
ZNF181→
LINC01838→
HPN→
FXYD1→
LSR→
FFAR1→
←KRTDAP
SCGB2B3P→
←LINC01801
ZNF30→
←HPN−AS1
USF2→
FFAR3→
←DMKN
ZNF302→
←ZNF30−AS1
FXYD3→
HAMP→
LINC01531→
←SBSN
←ZNF792
MIR6887→
MAG→
GAPDHS→
GRAMD1A→
FXYD7→
MIR5196→
TMEM147→
FXYD5→
GPR42→
←ATP4A
←TMEM147−AS1
35.2
35.4
35.6
35.8
36
Position on chr19 (Mb)

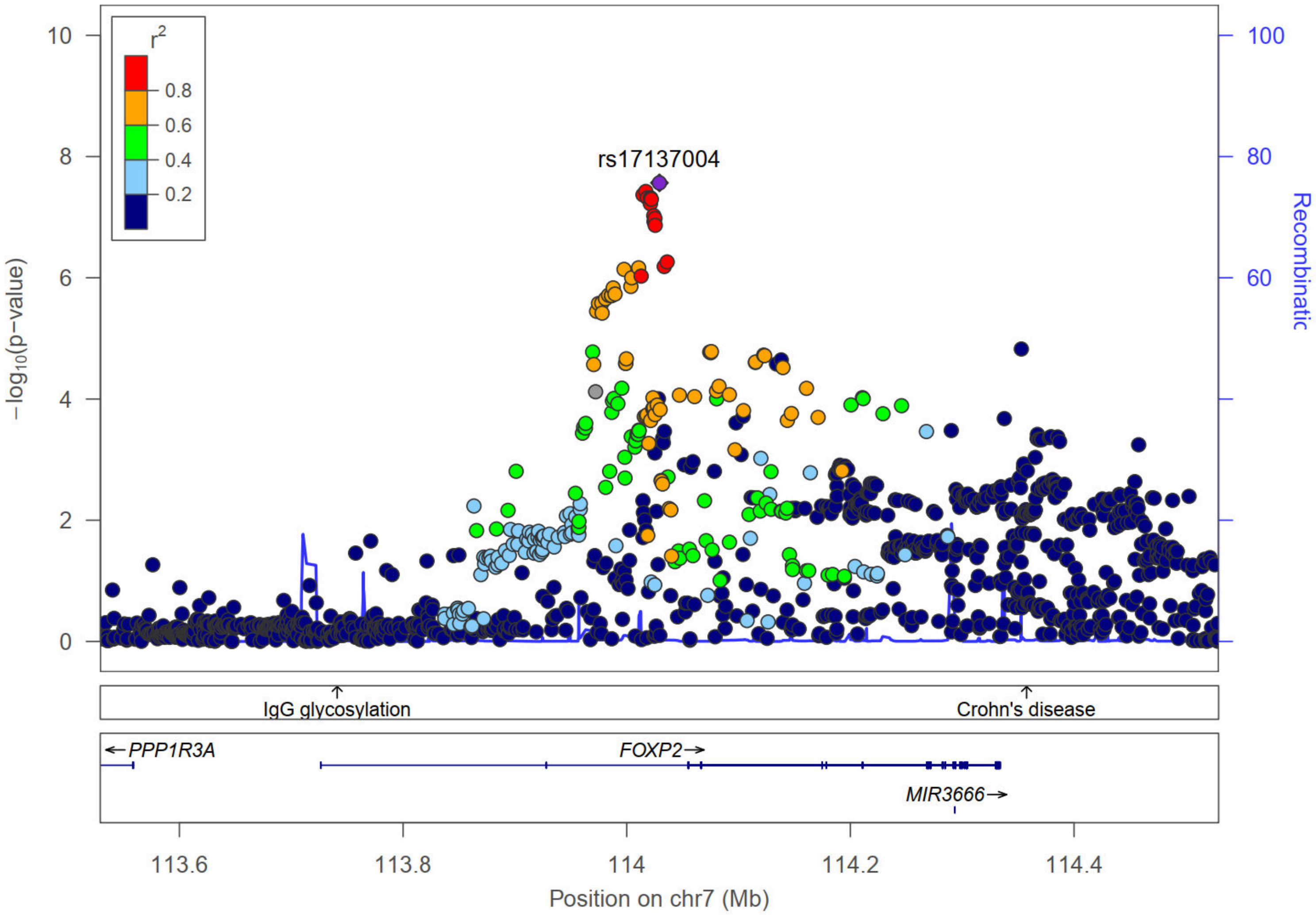

r2
0.8
0.6
0.4
0.2
rs17137004
−log10(p−value)
Recombinatic
IgG glycosylation
Crohn's disease
PPP1R3A
FOXP2
MIR3666
113.6
113.8
114
114.2
114.4
Position on chr7 (Mb)

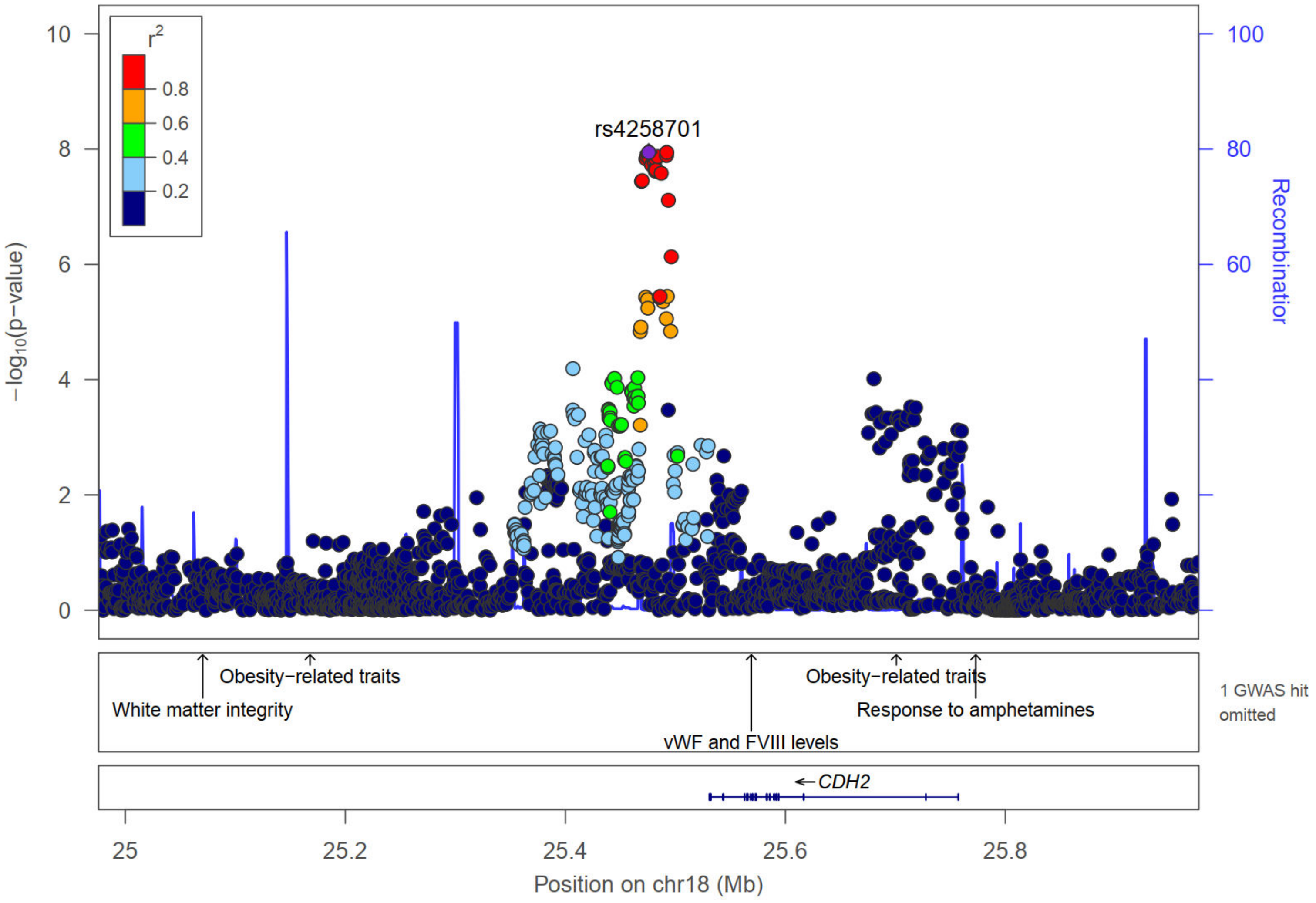

r2
0.8
0.6
0.4
0.2
rs4258701
10
8
6
4
2
0
100
80
60
Recombination
−log10(p−value)
White matter integrity
Obesity−related traits
vWF and FVIII levels
Obesity−related traits
Response to amphetamines
1 GWAS hit omitted
CDH2
25
25.2
25.4
25.6
25.8
Position on chr18 (Mb)

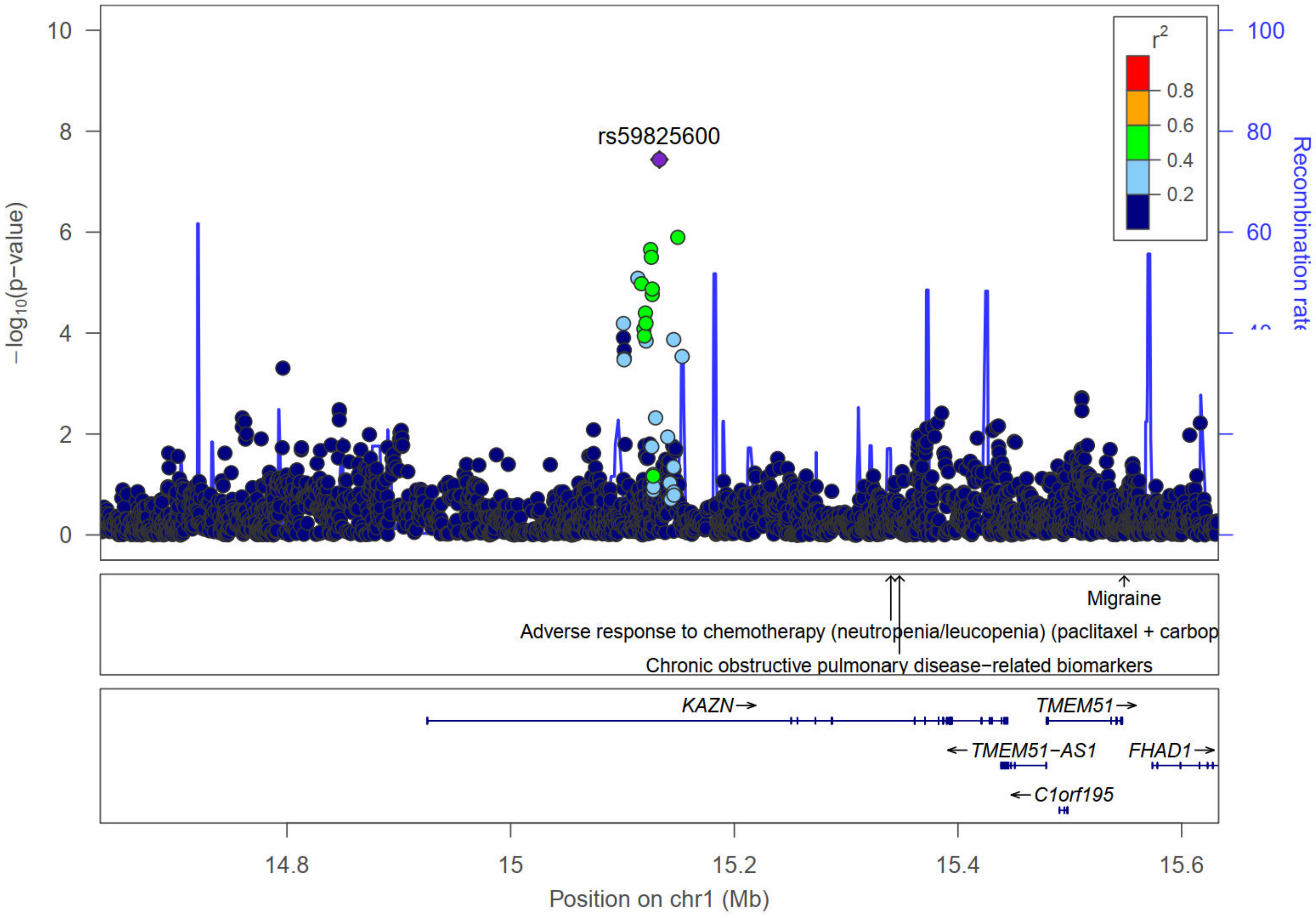
rs59825600
r2
0.8
0.6
0.4
0.2
−log10(p−value)
Recombination rate
10
8
6
4
2
0
100
80
60
Migraine
Adverse response to chemotherapy (neutropenia/leucopenia) (paclitaxel + carbop
Chronic obstructive pulmonary disease−related biomarkers
KAZN→
TMEM51→
←TMEM51−AS1
FHAD1→
←C1orf195
14.8
15
15.2
15.4
15.6
Position on chr1 (Mb)

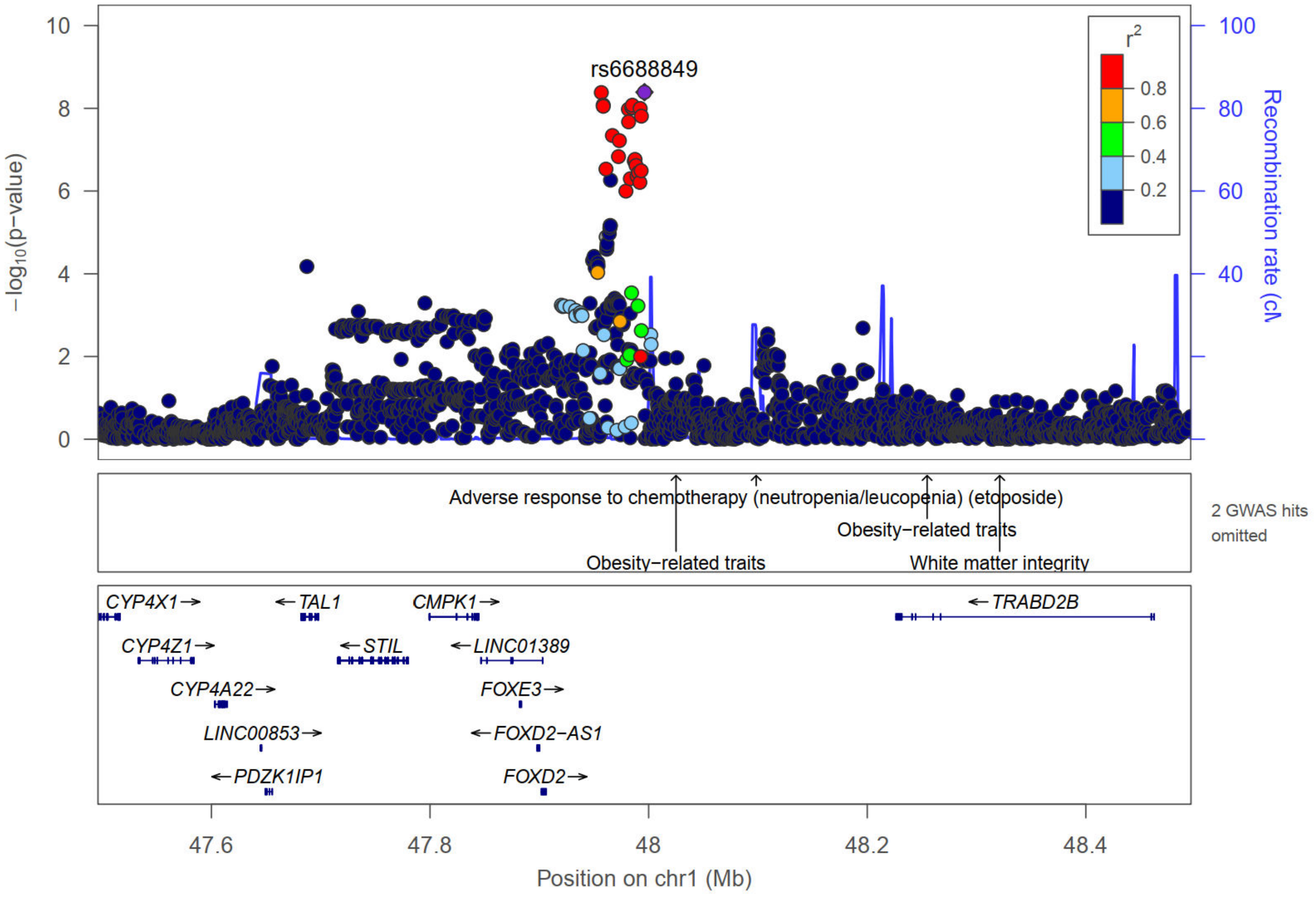

rs6688849
r2
0.8
0.6
0.4
0.2
−log10(p−value)
Recombination rate (cM
Adverse response to chemotherapy (neutropenia/leucopenia) (etoposide)
Obesity−related traits
Obesity−related traits
White matter integrity
2 GWAS hits omitted
CYP4X1→
←TAL1
CMPK1→
←TRABD2B
CYP4Z1→
←STIL
←LINC01389
CYP4A22→
FOXE3→
LINC00853→
←FOXD2−AS1
←PDZK1IP1
FOXD2→
47.6
47.8
48
48.2
48.4
Position on chr1 (Mb)

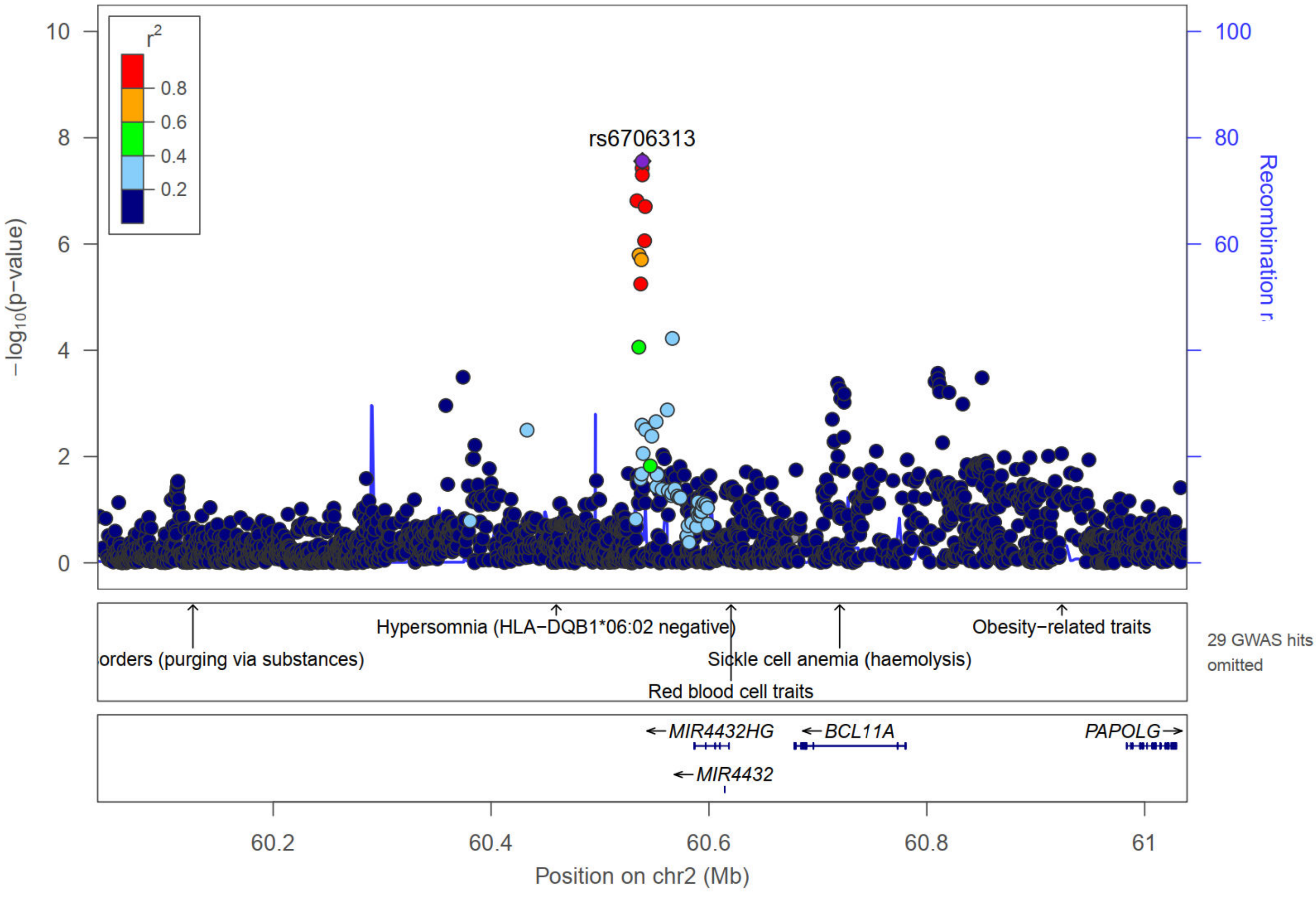

10
8
6
4
2
0
−log10(p−value)
r2
0.8
0.6
0.4
0.2
rs6706313
100
80
60
Recombination r.
orders (purging via substances)
Hypersomnia (HLA−DQB1*06:02 negative)
Sickle cell anemia (haemolysis)
Red blood cell traits
Obesity−related traits
29 GWAS hits omitted
MIR4432HG
BCL11A
PAPOLG
MIR4432
60.2
60.4
60.6
60.8
61
Position on chr2 (Mb)

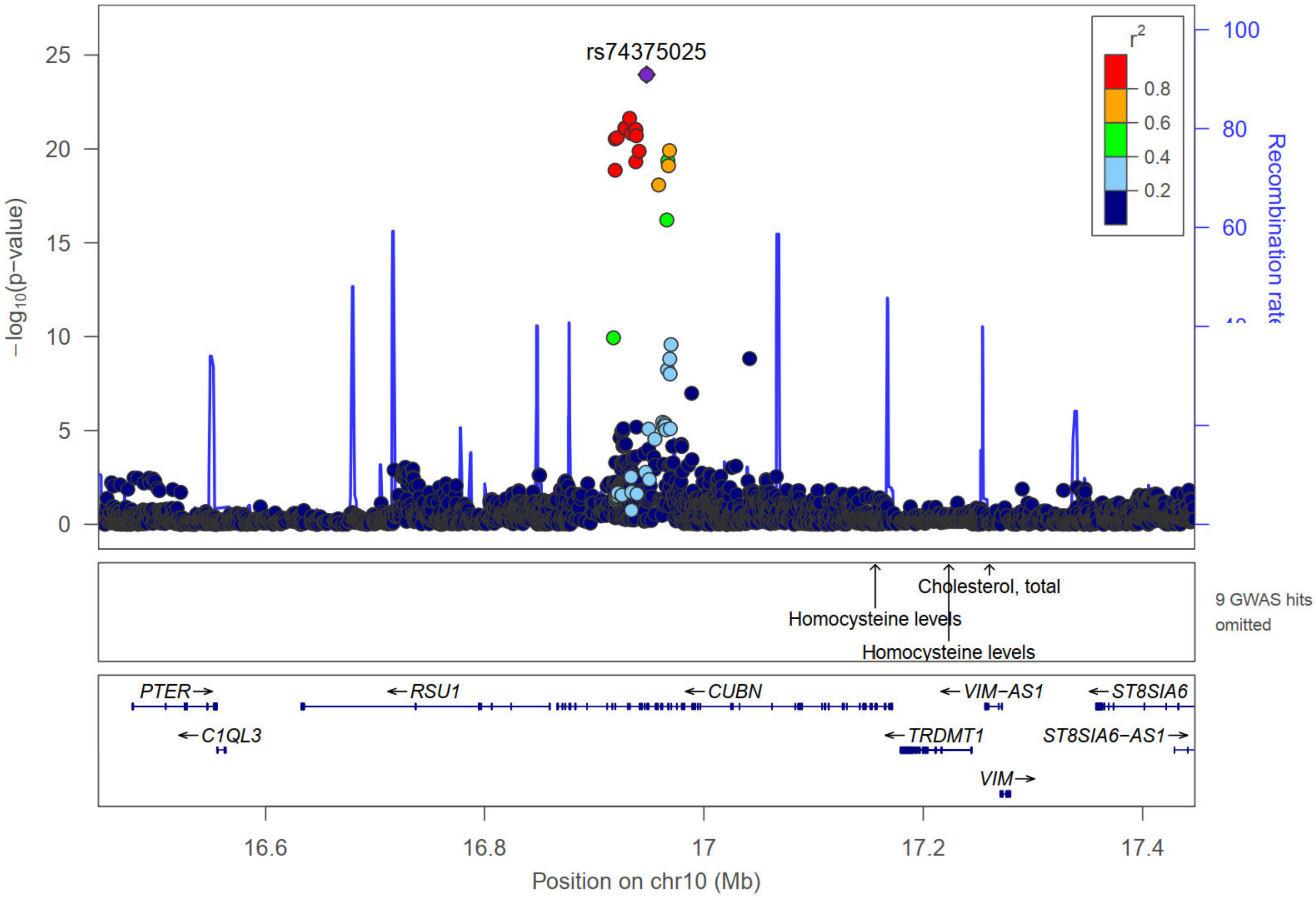
rs74375025
r2
0.8
0.6
0.4
0.2
100
80
60
Recombination rate
25
20
15
10
5
0
−log10(p−value)
Cholesterol, total
Homocysteine levels
Homocysteine levels
9 GWAS hits omitted
PTER
RSU1
CUBN
VIM−AS1
ST8SIA6
C1QL3
TRDMT1
ST8SIA6−AS1
VIM
16.6
16.8
17
17.2
17.4
Position on chr10 (Mb)

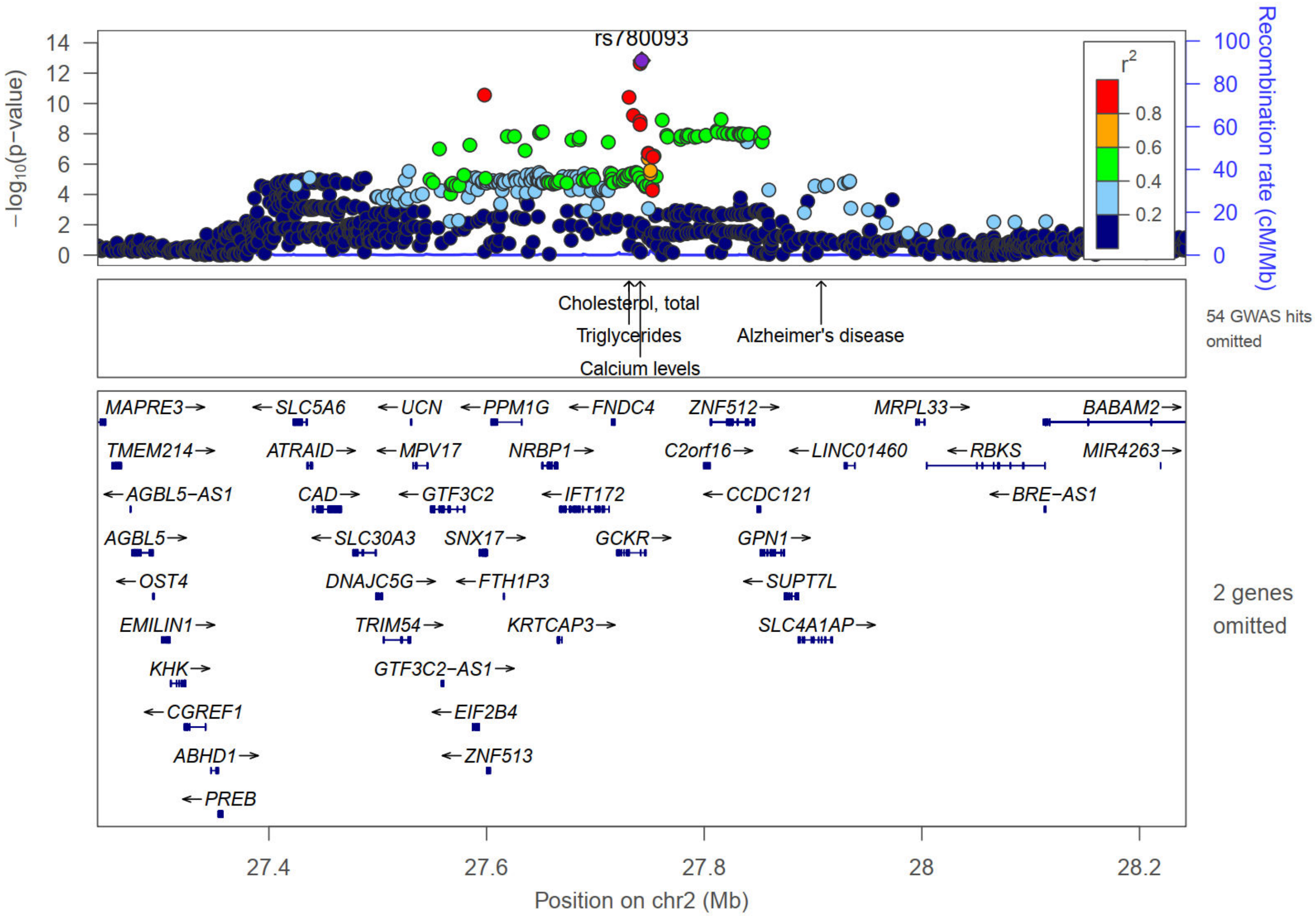

rs780093
−log10(p−value)
Recombination rate (cM/Mb)
r2
Cholesterol, total
Triglycerides
Calcium levels
Alzheimer's disease
54 GWAS hits omitted
MAPRE3→
←SLC5A6
←UCN
←PPM1G
←FNDC4
ZNF512→
MRPL33→
BABAM2→
TMEM214→
ATRAID→
←MPV17
NRBP1→
C2orf16→
←LINC01460
←RBKS
MIR4263→
←AGBL5−AS1
CAD→
←GTF3C2
←IFT172
←CCDC121
←BRE−AS1
AGBL5→
←SLC30A3
SNX17→
GCKR→
GPN1→
←OST4
DNAJC5G→
←FTH1P3
←SUPT7L
EMILIN1→
TRIM54→
KRTCAP3→
SLC4A1AP→
KHK→
GTF3C2−AS1→
←CGREF1
←EIF2B4
ABHD1→
←ZNF513
←PREB
2 genes omitted
Position on chr2 (Mb)

Regional association plots are shown for all genome-wide significant index SNPs. The dots are plotted on the y-axis according to their meta-analysis association p-value, and colored according to their correlation r^2 with the index SNP estimated based on the 1000 Genomes EUR reference samples (gray for missing data). Plots were generated using the stand-alone version 1.3 of LocusZoom[2]. Genetic positions refer to GRCh37/hg19 coordinates.

Supplementary Figure 5: Manhattan plots for all secondary GWAS meta-analyses

a

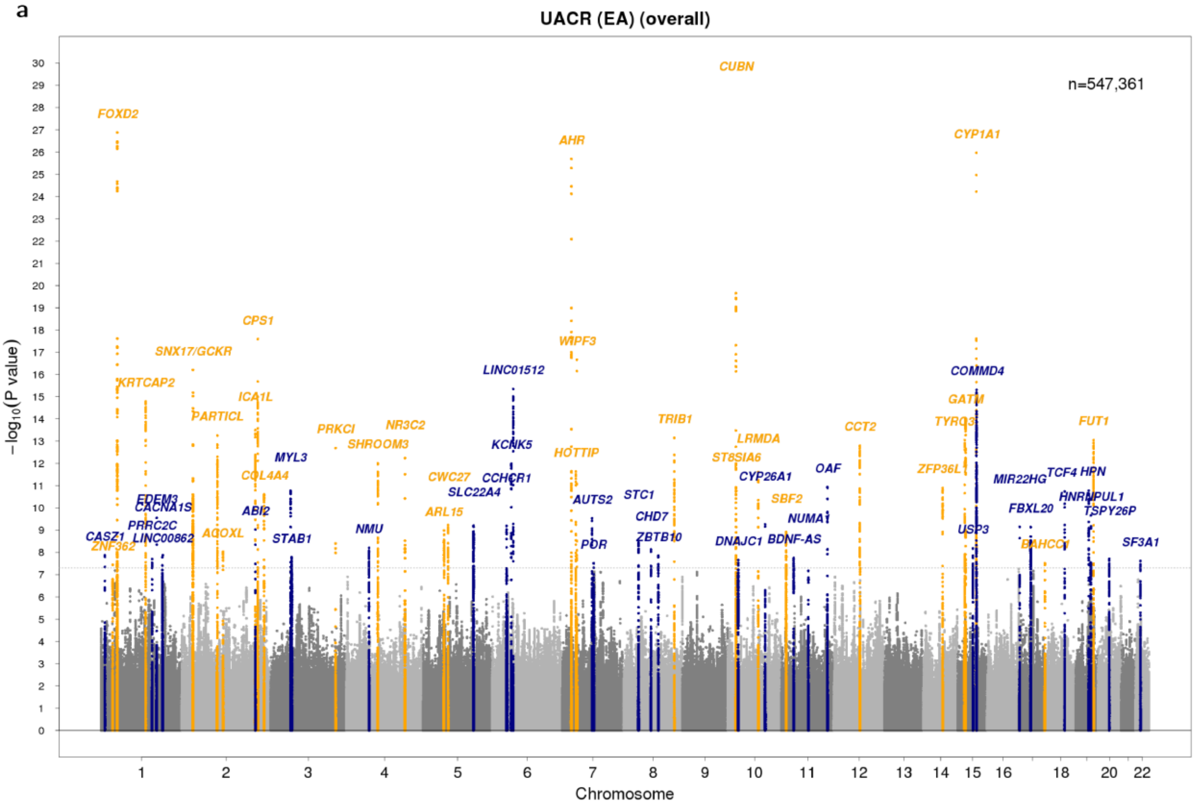

b

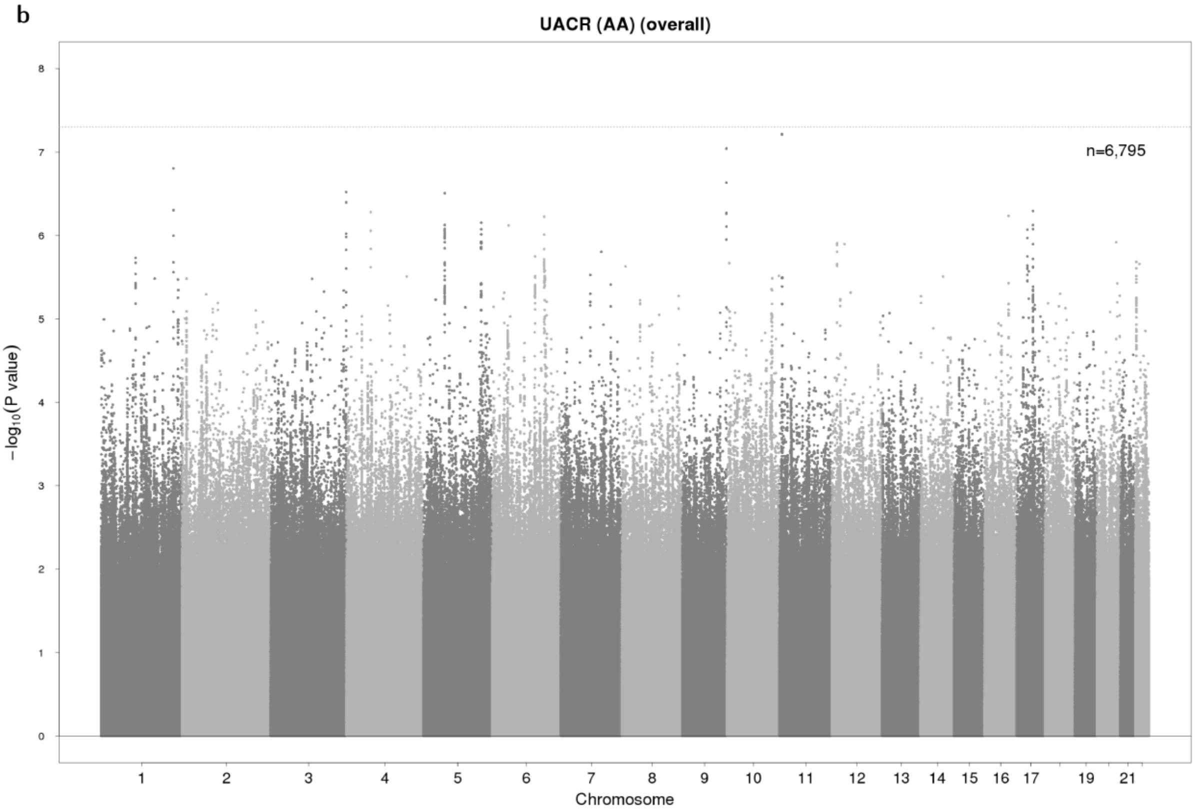

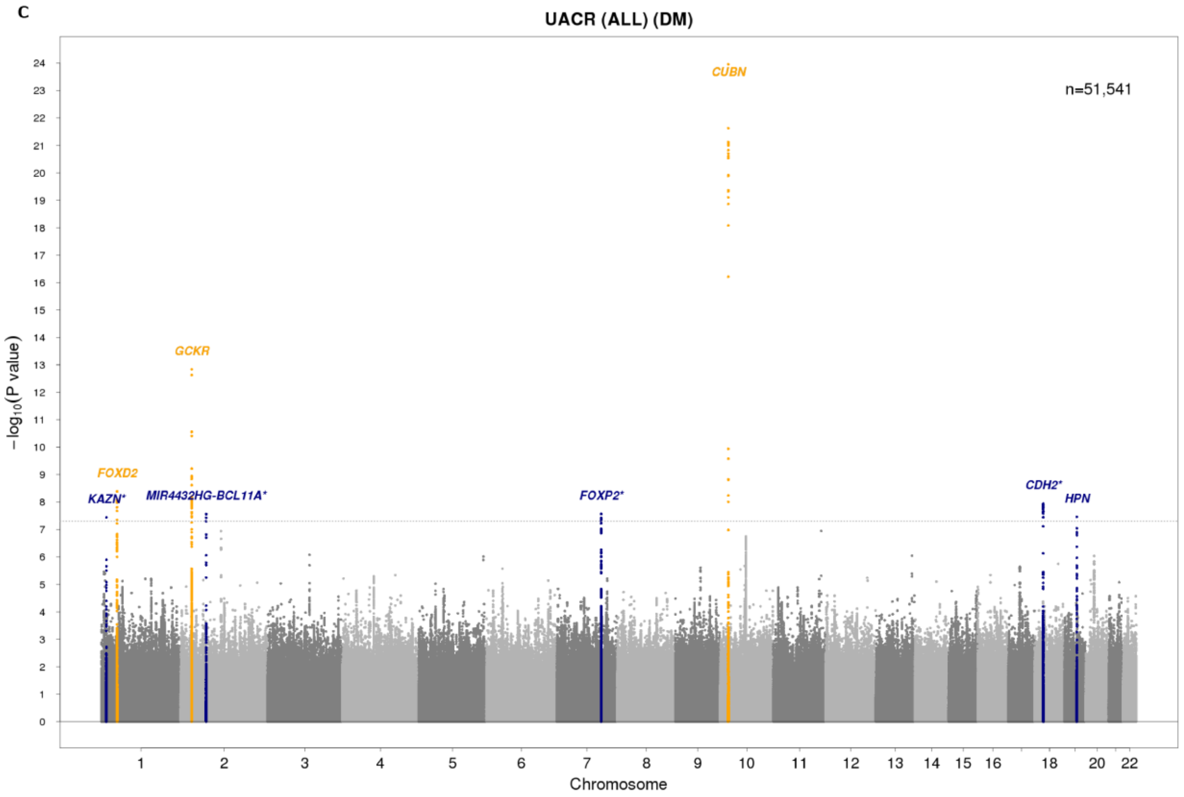

Manhattan plots of the GWAS meta-analysis results for UACR in individuals of European ancestry (a), in individuals of African ancestry (b), and among trans-ethnic individuals with diabetes (c). SNPs are plotted on the x-axis according to their position on each chromosome with the $-\log_{10}$(p-value) of the meta-analysis association test on the y-axis. The solid horizontal line indicates the threshold for genome-wide significance, $5x10^{-8}$. The labels show the closest gene. New loci are colored in blue, previously published UACR loci in orange. Genomic loci in panel (c) that are specific for individuals with diabetes are marked with an asterisk.

Supplementary Figure 6: QQ plots plots for all GWAS meta-analyses

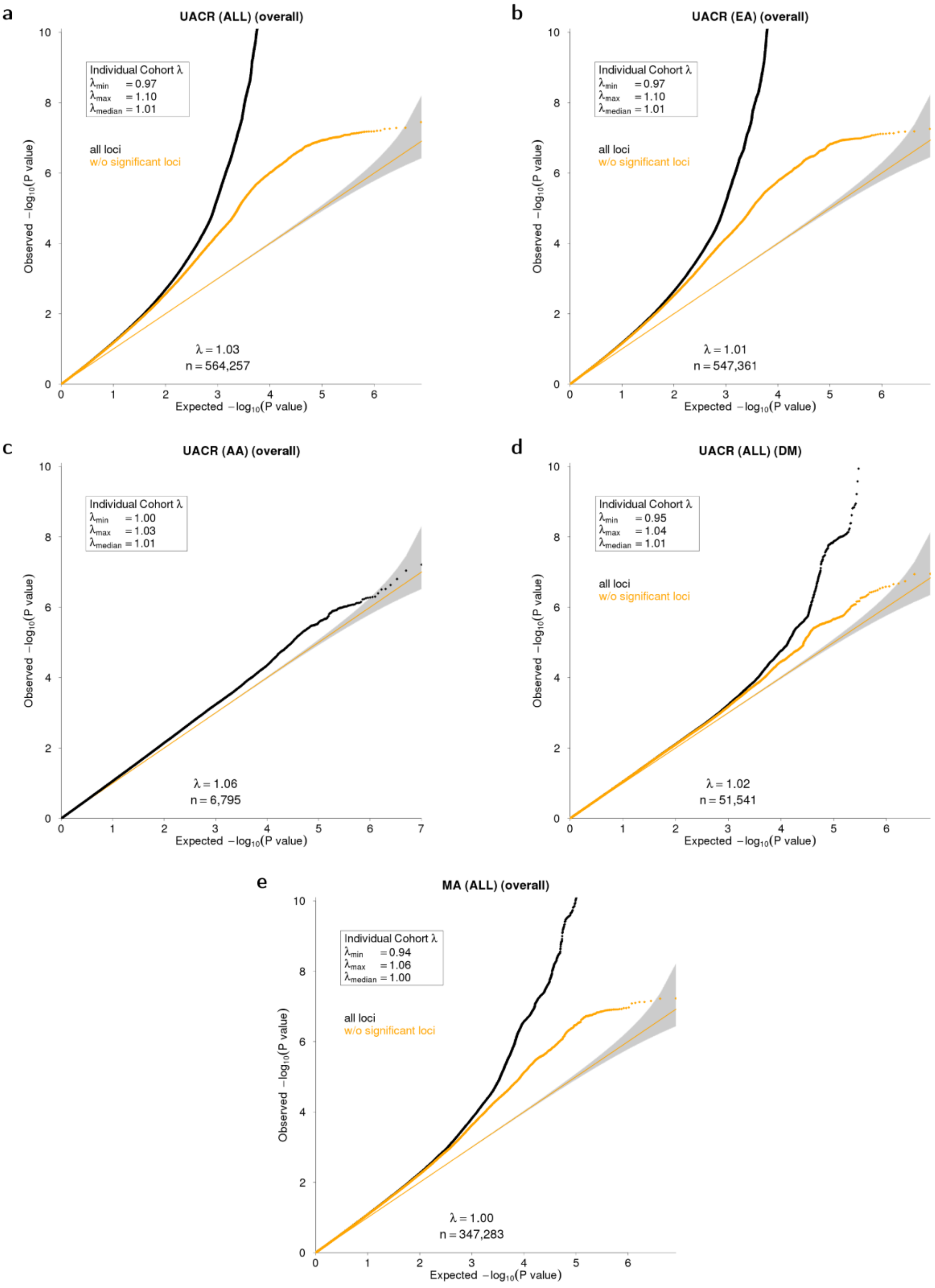

Quantile-quantile (QQ) plots of the GWAS meta-analysis results for UACR in the trans-ethnic sample (a), in individuals of European ancestry (b), in individuals of African ancestry (c), among trans-ethnic individuals with diabetes (d), and for trans-ethnic microalbuminuria meta-analysis (e). The observed p-values of the meta-analysis association test are plotted on the y-axis against their expected distribution under the null hypothesis of no association on the x-axis. Results for all SNPs are shown in black, and results after removal of loci (±500kb of index SNP) genome-wide significantly ($p<5x10^{-8}$) associated with the trait are shown in yellow (the African ancestry GWAS revealed no genome-wide significant associations). Gray bands represent 95% confidence intervals. λ: lambda, genomic control parameter; n: sample size.

Supplementary Figure 7: Enrichment analysis results for physiological system where UACR-associated genes are highly expressed

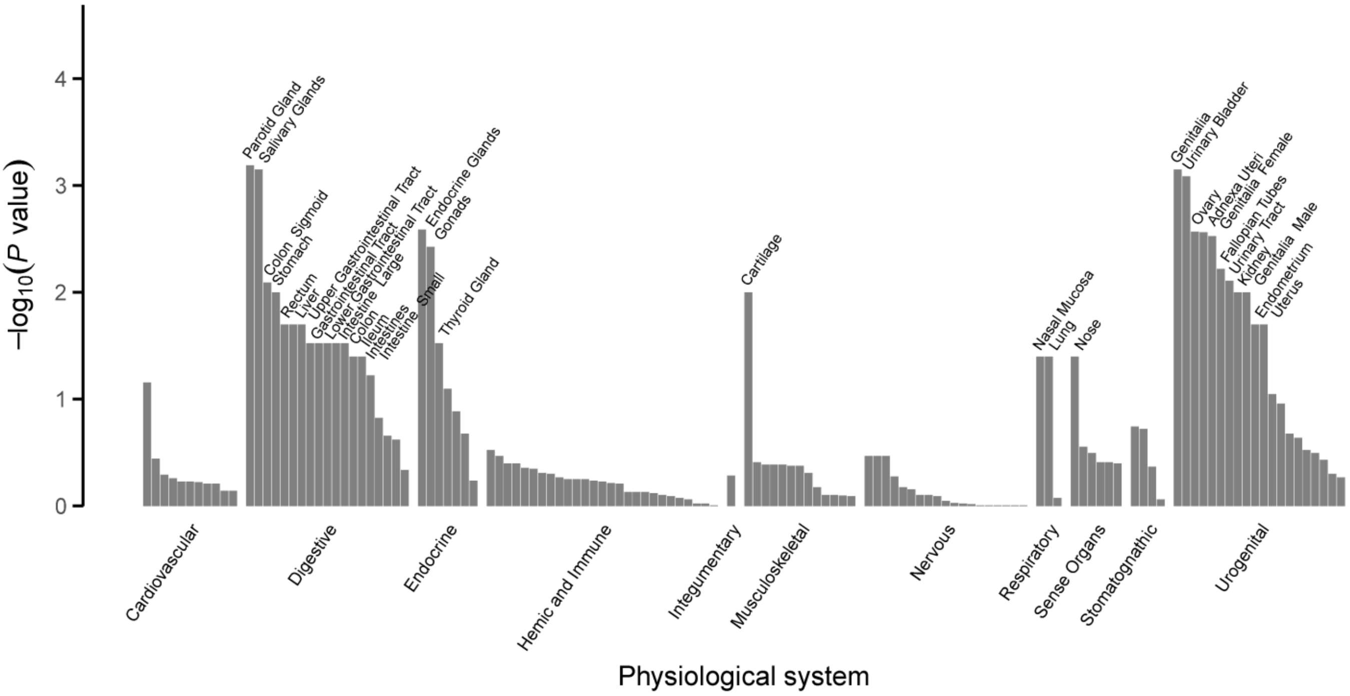

Results for enrichment of physiological systems based on the trans-ethnic UACR GWAS meta-analysis obtained by DEPICT software are shown. The x-axis is labeled by MeSH first level terms. Nominally significant associations ($p<0.05$) are labeled in the figure. The y-axis represents the $-\log_{10}$(p-value) of the enrichment test implemented in DEPICT.

Supplementary Figure 8: Co-localization of UACR-association signals with gene expression in *cis* across 47 human tissues

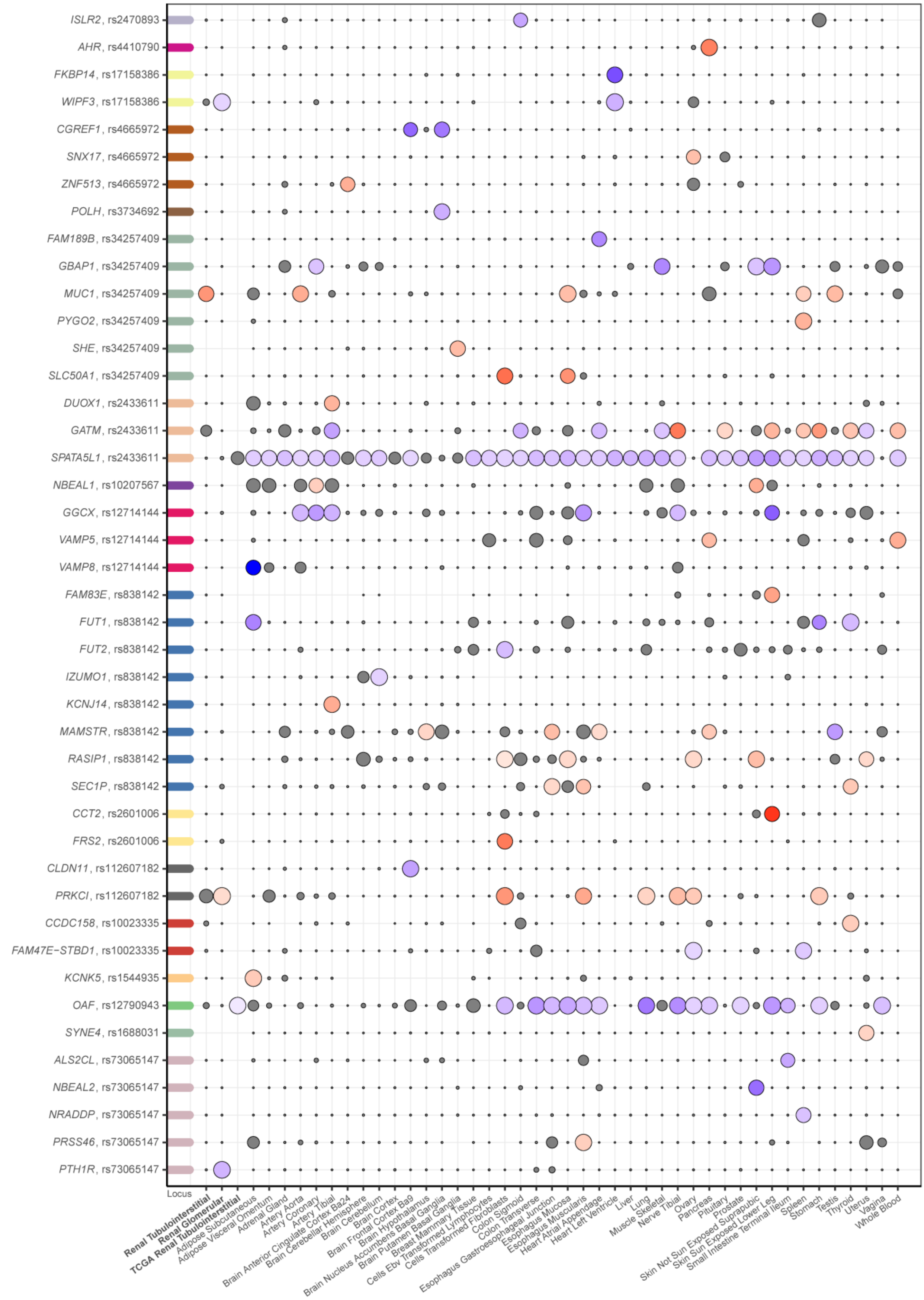

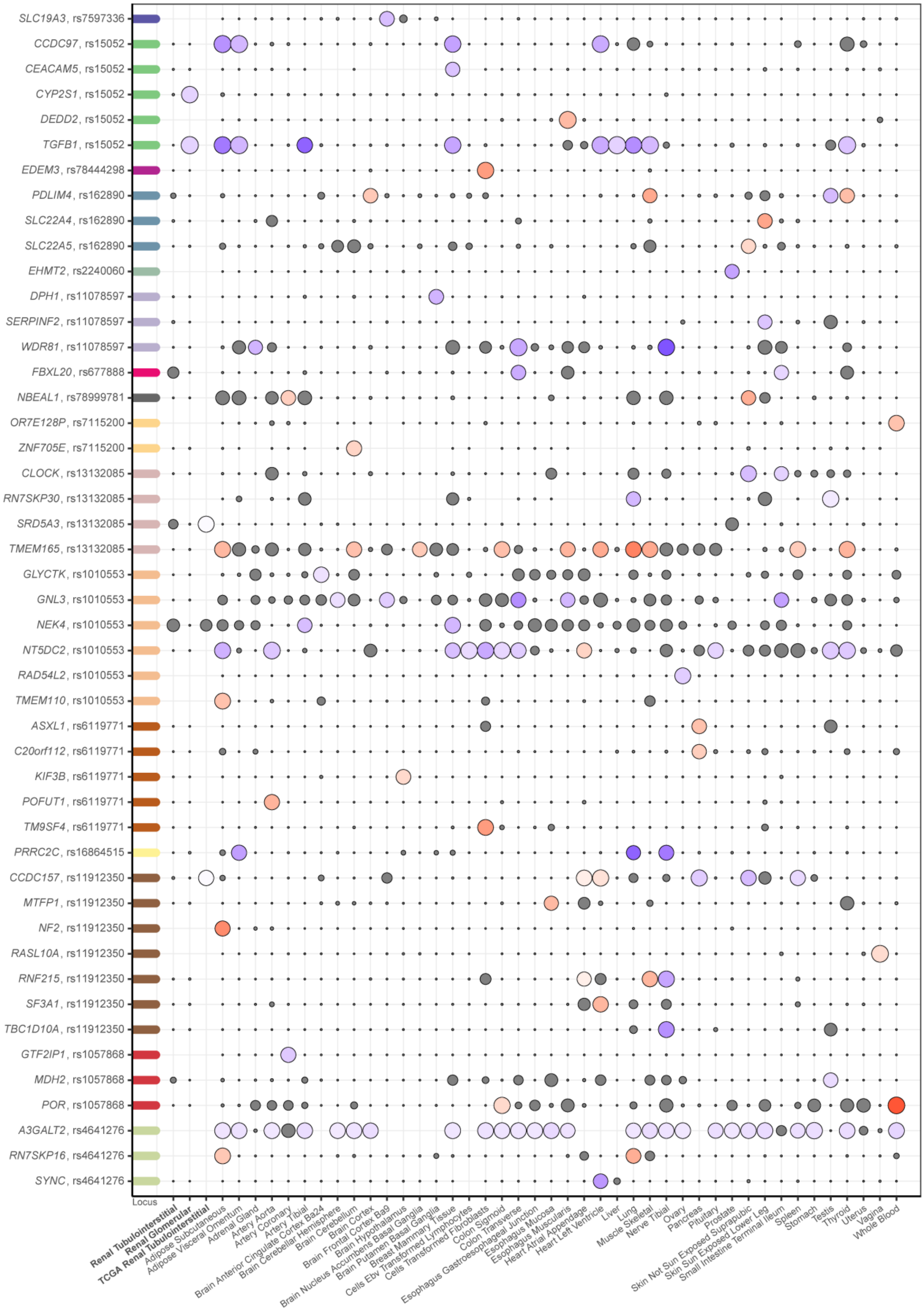

SLC19A3, rs7597336
CCDC97, rs15052
CEACAM5, rs15052
CYP2S1, rs15052
DEDD2, rs15052
TGFB1, rs15052
EDEM3, rs78444298
PDLIM4, rs162890
SLC22A4, rs162890
SLC22A5, rs162890
EHMT2, rs2240060
DPH1, rs11078597
SERPINF2, rs11078597
WDR81, rs11078597
FBXL20, rs677888
NBEAL1, rs78999781
OR7E128P, rs7115200
ZNF705E, rs7115200
CLOCK, rs13132085
RN7SKP30, rs13132085
SRD5A3, rs13132085
TMEM165, rs13132085
GLYCTK, rs1010553
GNL3, rs1010553
NEK4, rs1010553
NT5DC2, rs1010553
RAD54L2, rs1010553
TMEM110, rs1010553
ASXL1, rs6119771
C20orf112, rs6119771
KIF3B, rs6119771
POFUT1, rs6119771
TM9SF4, rs6119771
PRRC2C, rs16864515
CCDC157, rs11912350
MTFP1, rs11912350
NF2, rs11912350
RASL10A, rs11912350
RNF215, rs11912350
SF3A1, rs11912350
TBC1D10A, rs11912350
GTF2IP1, rs1057868
MDH2, rs1057868
POR, rs1057868
A3GALT2, rs4641276
RN7SKP16, rs4641276
SYNC, rs4641276
Locus
Renal Tubulointerstitial
Renal Glomerular
TCGA Renal Tubulointerstitial
Adipose Subcutaneous
Adipose Visceral Omentum
Adrenal Gland
Artery Aorta
Artery Coronary
Artery Tibial
Brain Anterior Cingulate Cortex Ba24
Brain Cerebellar Hemisphere
Brain Cerebellum
Brain Cortex
Brain Frontal Cortex Ba9
Brain Hypothalamus
Brain Nucleus Accumbens Basal Ganglia
Brain Putamen Basal Ganglia
Breast Mammary Tissue
Cells Ebv Transformed Lymphocytes
Cells Transformed Fibroblasts
Colon Sigmoid
Colon Transverse
Esophagus Gastroesophageal Junction
Esophagus Mucosa
Esophagus Muscularis
Heart Atrial Appendage
Heart Left Ventricle
Liver
Lung
Muscle Skeletal
Nerve Tibial
Ovary
Pancreas
Pituitary
Prostate
Skin Not Sun Exposed Suprapubic
Skin Sun Exposed Lower Leg
Small Intestine Terminal Ileum
Spleen
Stomach
Testis
Thyroid
Uterus
Vagina
Whole Blood

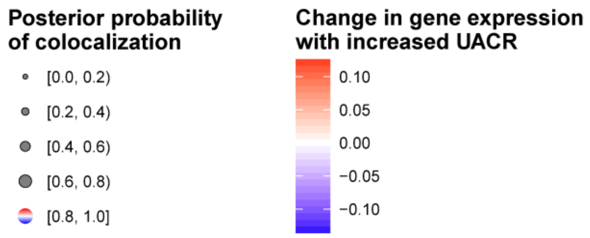

The plot shows genes whose expression levels in any tested tissues co-localized with the UACR association signal with a posterior probability of ≥80%. Genes and their respective index SNP are shown on the y-axis. Co-localization across tissues (x-axis) is illustrated as dots, where the size of the dots indicates the posterior probability of the co-localization. The change in UACR is color-coded relative to the change in gene expression, or gray in case of a posterior probability <0.80. Kidney tissues are labeled in bold.

Supplementary Figure 9: The *Drosophila OAF* and *PRKCI* are both required for nephrocyte function and PRKCI-RNAi affects slit diaphragm formation

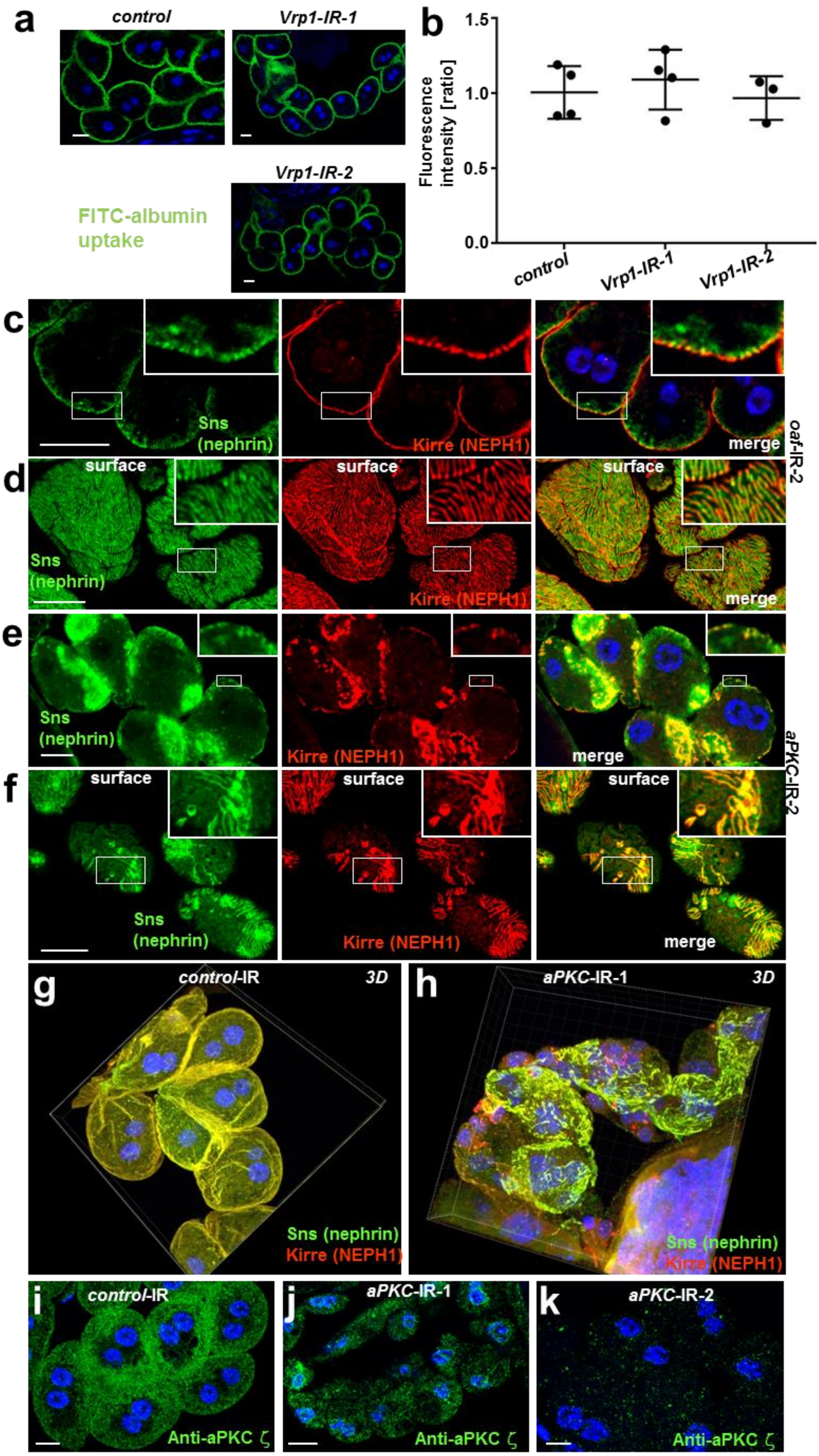

(a) Garland cell nephrocytes were exposed to FITC-albumin. Nephrocytes expressing control-RNAi exhibit intense endocytosis of FITC-albumin. Expression of *Vrp1*-RNAi has no significant effect on FITC-albumin uptake. (b) Quantitation of fluorescence intensity from FITC-albumin uptake is shown for the indicated genotypes. Values are presented as mean±standard deviation of the ratio to a control experiment. Statistical significance was calculated using ANOVA and Dunnett's *post hoc* analysis. A statistically significant difference (defined as $p<0.05$) was not observed (N=4 for *Vrp1*-RNAi-1, N=3 for *Vrp1*-RNAi-2).
(c) Staining the slit diaphragm proteins Sns (ortholog of nephrin) and Kirre (ortholog of NEPH1) in nephrocytes expressing *oaf*-RNAi-2 shows regular formation of slit diaphragms on the entire nephrocyte surface. (d) Tangential sections through the surface of control nephrocytes reveals the regular fingerprint-like pattern of slit diaphragm proteins.
(e-f) Expression of *aPKC*-RNAi-2 results in an clustered and irregular pattern of slit diaphragm proteins (inset in e) and a complete loss of slit diaphragm protein from distinct areas on the cell surface (insets f).
3D reconstructions from sequential confocal sections of nephrocytes expressing (g) control-RNAi and (h) *aPKC*-RNAi are shown. The surface is completely covered but slit diaphragm protein in the control but upon silencing aPKC large gaps appear.
(i-k) Staining control garland cell nephrocytes using anti-aPKC ζ, an antibody that is known to detect Drosophila aPKC, reveals a punctate staining pattern that is concentrated at the periphery of the cell including the membrane (i). Expression of *aPKC*-RNAi-1 (j) and *aPKC*-RNAi-2 (k) results in a distinct reduction of the antibody signal from the cell surface and periphery. This indicates a successful knockdown of aPKC on the protein level. Images were recorded at identical settings of the confocal microscope.
All scale bars represent 10 μm.

Supplementary Table 1: Association results of the UACR genetic risk score quartiles with microalbuminuria in the UKBB

Quartile	Effect	SE	Odds ratio (95% CI)	P-value	N cases	N controls
I			reference		6,281	54,096
II	0.159	0.019	1.17 (1.13-1.22)	2.24E-17	7,001	51,618
III	0.282	0.018	1.33 (1.28-1.38)	7.45E-53	7,583	49,799
IV	0.527	0.018	1.69 (1.64-1.75)	3.04E-191	9,149	47,224

Supplementary Table 2: Association results of the genetic risk score (GRS) analyses of UACR and it's risk factors

Exposure	Outcome	N SNPs	Effect	SE	OR (95% CI)	P-value	N cases	N total	Interpretation of effect
UACR	CAD	57	0.003	0.085	1.00 (0.85-1.18)	0.975	60,801	184,305	change in log(OR) of CAD per SD unit increase of log(UACR)
UACR	HF	59	0.398	0.158	1.49 (1.09-2.03)	0.012	6,065	408,313	change in log(OR) of HF per SD unit increase of log(UACR)
UACR	HTN	59	0.386	0.041	1.47 (1.36-1.59)	**2.38E-21**	226,011	408,539	change in log(OR) of HTN per SD unit increase of log(UACR)
UACR	Stroke	57	0.145	0.074	1.16 (1.00-1.34)	0.049	67,162	521,612	change in log(OR) of Stroke per SD unit increase of log(UACR)
DBP	UACR	319	0.007	0.001	-	**1.23E-24**	-	757,601	change of SD unit of log(UACR) per mm Hg DBP increase
SBP	UACR	244	0.008	0.001	-	**3.52E-63**	-	757,601	change of SD unit of log(UACR) per mm Hg SBP increase
T2DM	UACR	138	0.018	0.003	-	**1.01E-10**	62,892	659,316	change of SD unit of log(UACR) per unit increase in T2DM GRS

N SNPs gives the number of SNPs included in the genetic risk score. Significant P-values are marked bold.
N cases: number of cases; N total: total sample size of the non-UACR trait; OR [95% CI]: Odds ratio with 95% confidence interval; SD: standard deviation

CAD: coronary artery disease[3]

HF: heart failure (assessed in the UK BioBank)

HTN: hypertention (assessed in the UK BioBank)

Stroke: any stroke[4]

DBP, SBP: diastolic and systolic blood pressure[5]

T2DM: type 2 diabetes[6]

Supplementary Table 3: Evidence for additional candidate causal genes at UACR-associated variants

Gene	SNP	credible set size	variant PP	functional consequence	CADD	DHS	Brief summary of literature and gene function
EDEM3	rs78444298	2	0.92	p.Pro746Ser (NP_079467.3)	24.6	-	The protein encoded by the ER Degradation Enhancing Alpha-Mannosidase Like Protein 3 (EDEM3) gene belongs to a group of proteins that accelerate degradation of misfolded glycoproteins in the ER. It catalyzes mannose trimming of N-glycans. This variant has been reported in an exome chip study of eGFR (PMID: 27920155).
CUBN	rs45551835	1	1.00	p.Ala2914Val (NP_001072.2)	34.0	-	The cubilin gene (*CUBN*) has been reported in the first GWAS of UACR (PMID: 21355061) and has been replicated many times since. The encoded cubilin protein is important for reabsorption of filtered albumin in the proximal tubule. *CUBN* KO mice lose albumin in the urine (PMID: 21926402). Rare mutations cause aut-rec megaloblastic anemia-1, Finnish type (#261100). Patients show low-molecular weight proteinuria (PMID: 11717447).
CPS1	rs1047891	15	0.96	p.Thr1412Asn (NP_001116105.1)	22.1	-	Carbamoyl-Phosphate Synthase 1 (*CPS1*) encodes a key enzyme of the urea cycle that is important to remove excess urea. Rare mutations cause aut-rec carbamoylphosphate synthetase I deficiency (#237300). GWAS locus for eGFR (PMID: 20383146) and many other quantitative biomarkers. This variant has been reported to associate with hyperammonemia after valproate therapy (PMID: 23997965).
GCKR	rs1260326	5	0.09	p.Leu446Pro (NP_001477.2)	0.1	1*	The Glucokinase Regulator (*GCKR*) gene encodes a regulatory protein inhibiting glucokinase in pancreatic islet cells and liver. Has been identified in previous GWAS of serum albumin (PMID: 23022100), glycated albumin (PMID: 29844224) and multiple other cardiometabolic traits including diabetes.

This table includes all genes supported by missense variants with high posterior probability (>0.5) of association, or missense variants mapping into small credible sets of size ≤5.
1*: ENCODE kidney

Supplementary Table 4: Results of the trans-eQTL analysis

SNP meta-analysis	SNP Chr:Position (b37)	SNP p-value meta-analysis	Max. Posterior Probability meta-analysis	LD between SNP and eQTL-SNP (r^2)	eQTL-SNP	eQTL-SNP Chr:Position (b37)	eQTL-Gene	Chr eQTL Gene	Tissue source of eQTL study	p-value eQTL	inter-chromo-somal trans-eQTL
rs12714144	2:85754578	5.5E-14	0.194	0.97	chr2:85753464:D	2:85753464	*DPEP3*	16	*1	1.29E-14	yes
rs12714144	2:85754578	5.5E-14	0.194	0.97	2:85753464:TTTG_	2:85753464	*DPEP3*	16	*2	5.51E-11	yes
rs2240060	6:31114900	6.4E-10	0.183	1	rs115017941	6:31115604	*BTN3A2*	6	*1	3.08E-08	no
rs2240060	6:31114900	6.4E-10	0.183	1	6:31108485	6:31108485	*BTN3A2*	6	*2	2.70E-11	no
rs2240060	6:31114900	6.4E-10	0.183	1	rs115017941	6:31115604	*HLA-DRB1*	6	*1	3.21E-126	no
rs2240060	6:31114900	6.4E-10	0.183	1	6:31108485	6:31108485	*HLA-DRB1*	6	*2	1.82E-63	no

*1: PBMC and Whole Blood, LIFE A1&B3 study[7–9]

*2: Whole Blood, Framingham Heart Study[10]

Supplementary Table 5: Co-localization results of UACR association signals with those from associations with plasma protein levels

UACR SNP	UACR SNP chr	UACR SNP position	protein name	protein SNP	protein SNP chr	protein SNP position	protein SNP type	LD (r^2)	No. SNPs	PP H4 abf
rs12790943	11	120058623	OAF.6414.8.3	rs508205	11	120057343	conditional_hit	0.90	1526	0.99
rs1010553	3	52540773	AP4M1.10076.1.3	rs12493107	3	52706724	top_sentinel	0.50	1163	0.68
rs162890	5	131623658	C1QTNF5.7810.20.3	rs11955347	5	131567924	top_sentinel	0.41	1398	0.62
rs838142	19	49252151	CCL25.14068.29.3	rs35106244	19	49203829	top_sentinel	0.22	1492	0.55
rs838142	19	49252151	ALPI.10463.23.3	rs679574	19	49206108	top_sentinel	0.23	1492	0.53
rs12790943	11	120058623	OAF.6414.8.3	rs117554512	11	120098329	top_sentinel	0.00	1527	0.42
rs838142	19	49252151	FUT3.4548.4.2	rs679574	19	49206108	top_sentinel	0.23	1492	0.35
rs838142	19	49252151	FGF19.2762.30.2	rs601338	19	49206674	top_sentinel	0.24	1492	0.31
rs838142	19	49252151	GOLM1.8983.7.3	rs601338	19	49206674	top_sentinel	0.24	1492	0.27
rs838142	19	49252151	FAM177A1.8039.41.3	rs679574	19	49206108	top_sentinel	0.23	1492	0.26
rs838142	19	49252151	CCL15.14109.15.3	rs681343	19	49206462	top_sentinel	0.24	1492	0.25
rs838142	19	49252151	FAM3D.13102.1.3	rs601338	19	49206674	top_sentinel	0.24	1492	0.22
rs6119771	20	30770375	POFUT1.5634.39.3	rs35968884	20	30818823	conditional_hit	0.00	814	0.06
rs2240060	6	31114900	CREB3L4.11308.8.3	rs41558819	6	31324166	conditional_hit	0.00	6890	0.016
rs2240060	6	31114900	LRPAP1.3640.14.3	rs28572463	6	31209854	conditional_hit	0.01	6890	0.002
rs56043834	19	41331149	MIA.2687.2.1	rs2604877	19	41275048	top_sentinel	0.01	1672	0.0005
rs1010553	3	52540773	SEMA3G.5628.21.3	rs13091025	3	52467324	conditional_hit	0.11	1162	0.0003
rs35572189	17	79419025	ENTHD2.7947.19.3	rs61745945	17	79205421	top_sentinel	0.00	1858	0.0003
rs2240060	6	31114900	LRPAP1.8829.4.3	6:31149622	6	31149622	conditional_hit	0.01	6890	0.0002
rs56043834	19	41331149	MIA.2687.2.1	rs34532358	19	41288723	conditional_hit	0.08	1660	7.0E-05
rs2240060	6	31114900	GNLY.3195.50.2	rs558163186	6	31253305	top_sentinel	0.06	6891	2.1E-05
rs6119771	20	30770375	POFUT1.5634.39.3	rs76143353	20	30815755	top_sentinel	0.04	815	2.0E-05
rs15052	19	41813375	B3GNT8.9297.12.3	rs557663478	19	41933790	top_sentinel	0.00	1303	1.7E-05
rs12714144	2	85754578	GNLY.3195.50.2	rs10169536	2	85912860	conditional_hit	0.02	1503	1.6E-05
rs15052	19	41813375	B3GNT2.7980.72.3	rs67047091	19	41938684	top_sentinel	0.00	1303	1.5E-05
rs2240060	6	31114900	GZMA.3440.7.2	rs10569394	6	31240246	top_sentinel	0.06	6891	1.4E-05
rs7115200	11	71752160	STAB1.14599.18.3	rs139130389	11	71850156	top_sentinel	0.00	1417	1.4E-05
rs1010553	3	52540773	SEMA3G.5628.21.3	rs2016575	3	52477080	top_sentinel	0.06	1163	1.3E-05
rs7115200	11	71752160	CST8.10572.65.3	rs139130389	11	71850156	top_sentinel	0.00	1417	1.2E-05
rs2240060	6	31114900	LRPAP1.3640.14.3	rs41552714	6	31324756	top_sentinel	0.01	6891	2.1E-06
rs2240060	6	31114900	LRPAP1.8829.4.3	rs41552714	6	31324756	top_sentinel	0.01	6891	2.1E-06
rs2240060	6	31114900	CREB3L4.11308.8.3	rs9295987	6	31349844	top_sentinel	0.02	6891	1.6E-06
rs2240060	6	31114900	LILRB1.5090.49.2	rs141232332	6	31075601	top_sentinel	0.14	6891	1.5E-06
rs12790943	11	120058623	IL25.4137.57.2	rs2508490	11	120099679	top_sentinel	0.01	1527	4.1E-08
rs12714144	2	85754578	GNLY.3195.50.2	rs17430897	2	85912962	conditional_hit	0.01	1504	2.6E-10
rs12714144	2	85754578	GNLY.3195.50.2	rs12151621	2	85934499	top_sentinel	0.00	1505	1.3E-10
rs3734692	6	43817791	VEGFA.14032.2.3	rs6921438	6	43925607	top_sentinel	0.00	1507	1.2E-12
rs3734692	6	43817791	VEGFA.2597.8.3	rs6921438	6	43925607	top_sentinel	0.00	1507	1.2E-12

PP: posterior probability; LD: linkage disequilibrium; PP H4 abf: posterior probability of the H4 test (one common causal variant of trait and pQTL association)

References

1. GTEx Consortium *et al.* Genetic effects on gene expression across human tissues. *Nature* **550**, 204–213 (2017).
2. Pruim, R. J. *et al.* LocusZoom: regional visualization of genome-wide association scan results. *Bioinformatics* **26**, 2336–7 (2010).
3. Nikpay, M. *et al.* A comprehensive 1,000 Genomes-based genome-wide association meta-analysis of coronary artery disease. *Nat. Genet.* **47**, 1121–1130 (2015).
4. Malik, R. *et al.* Multiancestry genome-wide association study of 520,000 subjects identifies 32 loci associated with stroke and stroke subtypes. *Nat. Genet.* **50**, 524–537 (2018).
5. Evangelou, E. *et al.* Genetic analysis of over 1 million people identifies 535 new loci associated with blood pressure traits. *Nat. Genet.* **50**, 1412–1425 (2018).
6. Xue, A. *et al.* Genome-wide association analyses identify 143 risk variants and putative regulatory mechanisms for type 2 diabetes. *Nat. Commun.* **9**, 2941 (2018).
7. Kirsten, H. *et al.* Dissecting the genetics of the human transcriptome identifies novel trait-related trans-eQTLs and corroborates the regulatory relevance of non-protein coding loci†. *Hum. Mol. Genet.* **24**, 4746–63 (2015).
8. Beutner, F. *et al.* Rationale and design of the Leipzig (LIFE) Heart Study: phenotyping and cardiovascular characteristics of patients with coronary artery disease. *PLoS One* **6**, e29070 (2011).
9. Loeffler, M. *et al.* The LIFE-Adult-Study: objectives and design of a population-based cohort study with 10,000 deeply phenotyped adults in Germany. *BMC Public Health* **15**, 691 (2015).
10. Joehanes, R. *et al.* Integrated genome-wide analysis of expression quantitative trait loci aids interpretation of genomic association studies. *Genome Biol.* **18**, 16 (2017).
