# Supplementary material for: Genome-wide association meta-analyses and fine-mapping elucidate pathways influencing albuminuria
Source: Nat Commun. 2019 Sep 11;10:4130. doi: 10.1038/s41467-019-11576-0 (PMC6739370; doi:10.1038/s41467-019-11576-0)
Supplement: Supplementary file 17 — Description of Additional Supplementary Files [file 41467_2019_11576_MOESM17_ESM.pdf]

File Name: Supplementary Data 1.xlsx
Description: Study design and phenotype distribution of participating studies

File Name: Supplementary Data 2.xlsx
Description: Details on genotyping and imputation of participating studies

File Name: Supplementary Data 3.xlsx
Description: Index SNP characteristics at 59 loci associated with UACR in trans-ethnic meta-analysis

File Name: Supplementary Data 4.xlsx
Description: Associations of 59 trans-ethnic index SNPs for UACR in a meta-analysis of 53 CKDGen cohorts without UK Biobank and in UK Biobank only

File Name: Supplementary Data 5.xlsx
Description: Index SNP characteristics at 61 loci associated with UACR in meta-analysis of European-ancestry participants

File Name: Supplementary Data 6.xlsx
Description: Index SNP characteristics at 8 loci associated with UACR in meta-analysis of individuals with diabetes: CKDGen (including UK Biobank) as well as in CKDGen cohorts without UK Biobank and UK Biobank only, and compared to the overall sample of CKDGen

File Name: Supplementary Data 7.xlsx
Description: Enrichment analysis results for tissues/cell types where UACR-associated genes are highly expressed (DEPICT)

File Name: Supplementary Data 8.xlsx
Description: All gene sets with evidence of enrichment (at FDR<0.05) for genes mapping into UACR-associated loci (DEPICT)

File Name: Supplementary Data 9.xlsx
Description: Results of phenome-wide association study using a genetic risk score for UACR

File Name: Supplementary Data 10.xlsx
Description: Genome-wide genetic correlations between UACR and GWAS traits based on UK Biobank data

File Name: Supplementary Data 11.xlsx
Description: Characteristics at 63 conditionally independent SNPs associated with UACR in meta-analysis of European-ancestry individuals and their credible sets

File Name: Supplementary Data 12.xlsx
Description: Functional annotation of variants mapping into 99% credible sets with ≤5 variants or with a SNP with posterior probability of association (PP) >50%

File Name: Supplementary Data 13.xlsx
Description: Results of pQTL lookup
